# Annotated type catalogue of the Chrysididae (Insecta, Hymenoptera) deposited in the collection of Maximilian Spinola (1780–1857), Turin

**DOI:** 10.3897/zookeys.471.6558

**Published:** 2015-01-14

**Authors:** Paolo Rosa, Zai-fu Xu

**Affiliations:** 1 Via Belvedere 8/d, I-20881 Bernareggio (MB), Italy; 2 Department of Entomology, College of Natural Resources and Environment, South China Agricultural University, Guangzhou 510640, P. R. China

**Keywords:** Chrysididae, neotype, lectotype, new synonymy, *nomen dubium*, *nomen oblitum*, *nomen protectum*, Spinola collection

## Abstract

A critical and annotated catalogue of the ninety-six type specimens of Chrysididae (Hymenoptera), belonging to sixty-seven species, housed in the insect collection of Maximilian Spinola is given. The neotypes of six species are designated: *Chrysis bicolor* Lepeletier, 1806; *Chrysis comparata* Lepeletier, 1806; *Chrysis dives* Dahlbom, 1854; *Chrysis pumila* Klug, 1845; *Chrysis succincta* Linnaeus, 1767; *Hedychrum bidentulum* Lepeletier, 1806. The lectotypes of twenty-four species are designated: *Chrysis aequinoctialis* Dahlbom, 1854; *Chrysis analis* Spinola, 1808; *Chrysis assimilis* Dahlbom, 1854; *Chrysis bihamata* Spinola, 1838; *Chrysis chilensis* Spinola, 1851; *Chrysis dichroa* Dahlbom, 1854; *Chrysis distinguenda* Dahlbom, 1854; *Chrysis episcopalis* Spinola, 1838; *Chrysis grohmanni* Dahlbom, 1854; *Chrysis incrassata* Spinola, 1838; *Chrysis pallidicornis* Spinola, 1838; *Chrysis pulchella* Spinola, 1808; *Chrysis ramburi* Dahlbom, 1854; *Chrysis refulgens* Spinola, 1806; *Chrysis splendens* Dahlbom, 1854; *Chrysis succinctula* Dahlbom, 1854; *Chrysis versicolor* Spinola, 1808; *Elampus gayi* Spinola, 1851; *Hedychrum caerulescens* Lepeletier, 1806; *Hedychrum chloroideum* Dahlbom, 1854; *Hedychrum difficile* Spinola, 1851; *Hedychrum virens* Dahlbom, 1854; *Holopyga janthina* Dahlbom, 1854; *Holopyga luzulina* Dahlbom, 1854. Previous lectotype designations of five species are set aside: *Chrysis bicolor* Lepeletier, 1806 (designated by Morgan 1984); *Chrysis calimorpha* Mocsáry, 1882 (designated by Móczár 1965); *Chrysis elegans* Lepeletier, 1806 (designated by Bohart (in Kimsey and Bohart 1991)); *Hedychrum chloroideum* Dahlbom, 1854 (designated by Kimsey 1986); *Hedychrum rutilans* Dahlbom, 1854 (designated by Morgan 1984). Three new synonymies are proposed: *Hedychrum intermedium* Dahlbom, 1845, **syn. n.** of *Holopyga fervida* (Fabricius, 1781); *Chrysis sicula* Dahlbom, 1854, **syn. n.** of *Chrysis elegans* Lepeletier, 1806; *Chrysis succinctula* Dahlbom, 1854, **syn. n.** of *Chrysis germari* Wesmael, 1839. *Chrysis distinguenda* Spinola, 1838, and *Chrysis coronata* Spinola, 1808, are considered **nomina dubia.** *Hedychrum alterum* Lepeletier, 1806, and *Hedychrum aulicum* Spinola, 1843, are considered **nomina oblita.** *Hedychrum rutilans* Dahlbom, 1854, and *Hedychrum niemelai* Linsenmaier, 1959, are retained as **nomina protecta.** The first available name for *Chrysis succinctula* *sensu* Linsenmaier is *Chrysis tristicula* Linsenmaier, 1959, (**stat. n.**) The current status and validity of some types in the Spinola collection are discussed. Photographs of fifty-three types are given.

## Introduction

Maximilian Spinola was a very active entomologist and described hundreds of species in different families of Coleoptera, Hymenoptera, and Hemiptera (Gestro 1915). Spinola was also a famous collector, whose collection grew particularly with insects received from his properties in Spain and South America and in exchange with European entomologists. Additionally, he made expensive purchases of beetles and wasps from all over the world (Passerin d’Entréves 1980) and of partial or entire collections by other famous entomologists of his times, such as Lepelletier de Saint Fargeau (later simply written: Lepeletier) (e.g. Spinola letter 00576) and Serville (e.g. Spinola letter 01566). The history of this collection was fully reconstructed only in the 1980s thanks to the analysis of his impressive bulk of correspondence (Casolari and Casolari Moreno 1980, Passerin d'Entréves 1980). Maximilian Spinola was born in France, at Pézenas, Hérault, on July 10, 1780 and died in Italy, at Tassarolo, Piedmont, on November 12, 1857.

The hymenopteran collection of Maximilian Spinola is currently housed in the Museo Regionale di Scienze Naturali (MRSN) in Turin. However, it belongs to the Museo di Zoologia Sistematica dell’Università di Torino. The collection was organized following an old standard method: every species includes one to several specimens whose collecting data were removed from the insects and written on a main label at the bottom of the specimen-series. Every main label had a different colour depending on the provenances of the species: white (Europe), yellow (Asia), blue (Africa), green (Americas), rose (Australia). Every main label bears the generic and specific name, author of the species, the collectors, and the localities. Main references on Chrysididae related to this collection are: Spinola (1805, 1806–1808, 1838, 1840, 1851), Lepeletier (1806), Lepeletier and Serville (1825), Brullé (1846), Dahlbom (1854), and Kimsey and Bohart (1991). Many books and papers have been written on types of this collection, except on those referring to the hymenopteran family Chrysididae (e.g. Bradley 1957; Vidano and Arzone 1978; Casolari and Casolari 1980; Passerin d’Entréves 1980, 1983; Casale 1982; Giachino 1982; Olmi 1983; Baker 1999; Generani and Scaramozzino 2000).

**Figure 1.**
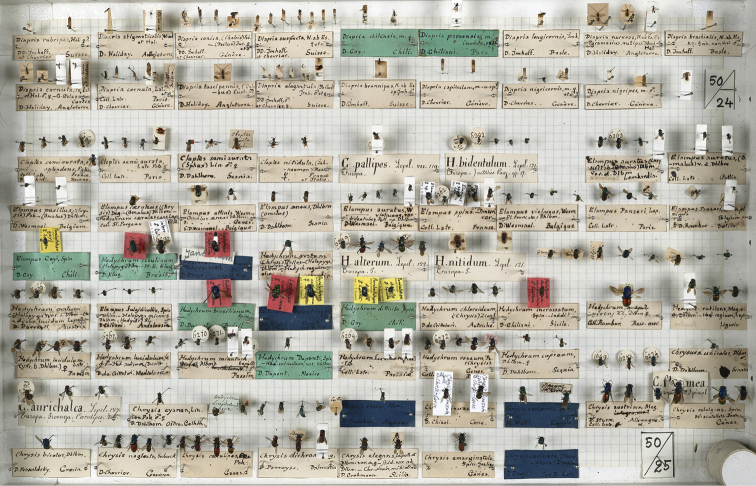
Spinola’s Hymenoptera collection, box 50 (photo courtesy of MRSN).

**Figure 2.**
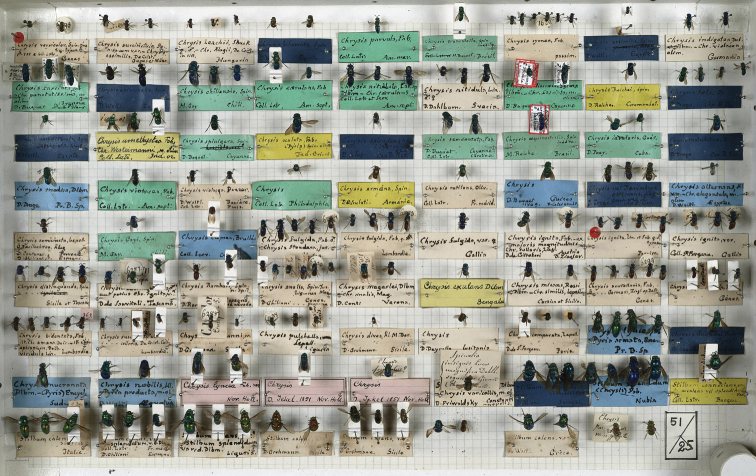
Spinola’s Hymenoptera collection, box 51 (photo courtesy of MRSN).

**Figure 3.**
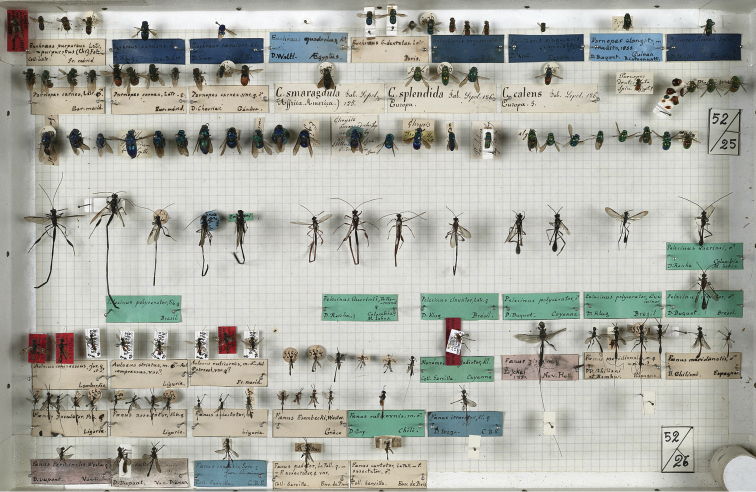
Spinola’s Hymenoptera collection, box 52 (photo courtesy of MRSN).

The collection of Chrysididae by Spinola includes 399 specimens housed in three boxes numbered 50, 51, and 52 (Figs 1–3). The collection consists of specimens collected by some of the most important entomologists of their time as well as insect traders: Dahlbom, Draege, Fischer, Gay, Ghiliani, Klug, Latreille, Lepeletier, Megerle, Rambur, Serville, Waltl, Wesmael, and Westermann (Passerin d’Entréves 1980, pers. comm.). The collection is still roughly in the same order as it was left by Spinola, but it is not in good condition. Some type specimens have been destroyed by dermestids, and only pins and labels are left in the respective boxes. The first dermestid attack dated back to the first years after the death of Spinola (in 1857) and before Abeille de Perrin (1879), who already commented on the bad status of the collection, was able to study Spinola’s collection. Also mechanical, accidental crushes and a layer of mould damaged the type material. The original Hymenoptera collection consisted of 69 boxes, later moved into new boxes preserving original labels and order left by Spinola (Casolari and Casolari Moreno (1980))].

The collection of Chrysididae by Spinola is very important, and it was fully studied and published by Spinola (1805, 1806–1808, 1838, 1840, 1851) and Dahlbom (1854). Spinola described many new species and even some new genera of Chrysididae (i.e., *Elampus* and *Stilbum*) that are still considered valid. Dahlbom, the first reviser of the family Chrysididae, described many new species based on Spinola’s chrysidid collection. However, only a few types from the Spinola collection are housed in Dahlbom’s collection in Lund (LZM); most of them are in Turin. The study of the type material preserved in this collection was fundamental to clarify some doubtful and incorrect identifications of the species in the current sense.

In the present paper, we identified ninety-six types, belonging to sixty-seven species (six neotypes, thirty-two holotypes, twenty-four lectotypes, twenty-seven paralectotypes, and thirteen syntypes) deposited in the Spinola collection. Three neotypes are designated in NMLS (*Chrysis bicolor* Lepeletier, 1806, *Chrysis succincta* Linnaeus, 1767 and *Hedychrum bidentulum* Lepeletier, 1806), and one neotype is designated in HNHM (*Chrysis calimorpha* Mocsáry, 1882) to ensure stability in the nomenclature of the Palaearctic Chrysididae.

## Material and methods

Terminology and classification of genera and species groups follow Kimsey and Bohart (1991), classification of the European species follows Linsenmaier (1959, 1968, 1987, 1997a, 1997b, 1999), Rosa (2006), and Rosa and Soon (2012). Abbreviations used in the text are as follows: F-I, F-II, F-III, etc. = flagellum I, flagellum II, flagellum III and so on; S-II = second metasomal sternum; S-III = third metasomal sternum. We report the codes of the catalogue Casolari and Casolari Moreno (1980), according to the same system already used for Spinola's catalogues (Pagliano 2005).

Since there are no published photographs of the types in Spinola’s collection and because some type species in this collection had been misinterpreted in the past, the present catalogue is illustrated with images taken from some types in the collection to facilitate future identifications.

Photographs of the types were taken with Nikon D-80 connected to the stereomicroscope Togal SCZ and stacked with the software Combine ZP; the white calibration of the photocamera was applied to reduce the blue effect of the neon light of the Togal microscope.

The definitions of holotype, neotype, lectotype etc. are used according to the International Code for Zoological Nomenclature (ICZN), fourth edition, later called "the Code". Types and other specimens have been examined from the following institutions:

HNHM Hungarian Natural History Museum, Budapest, Hungary.

IRSN Institut Royal des Sciences Naturelles, Bruxelles, Belgium.

ISEA–PAS Invertebrate collections of the Institute of Systematics and Evolution of Animals, Polish Academy of Sciences in Krakow, Poland.

LMU Linnémuseet, Uppsala, Sweden.

LSL Linnean Society of London, England.

LZM Lund Zoological Museum, University of Lund, Sweden.

MCZ Museo Civico di Zoologia, Roma, Italy.

MHNG Muséum d’Histoire Naturelle, Genève, Switzerland.

MNHN Muséum National d’Histoire Naturelle, Paris, France.

MNHU Museum für Naturkunde der Humboldt-Universität, Berlin, Germany.

MRSN Museo Regionale di Scienze Naturali, Turin, Italy.

MSNG Museo Civico di Storia Naturale "G. Doria", Genoa, Italy.

NHML Natural History Museum, London.

NHMW Naturhistorisches Museum Wien, Vienna, Austria.

NHRS Swedish Museum of Natural History, Stockholm, Sweden.

NMLS Natur Museum Luzern, Switzerland.

ZMUC Zoological Museum, University of Copenhagen, Denmark.

## Results and discussion

### Types housed in the Spinola collection

The types of the following sixty-seven species listed are housed in the Spinola collection.

#### Chrysis aequinoctialis

Taxon classificationAnimaliaHymenopteraChrysididae

Dahlbom, 1854

Plate 1

Chrysis aequinoctialis : Dahlbom 1854: 330.

##### Type locality.

"Habitat in Brasilia, Dom. Reiche; Mus. Dom. Spinola".

##### Material.

**Lectotype** (here designated) ♂: *Chrysis aequinoctialis* Spin. inédite D. Reiche, Bresil.

**Catalogue Casolari & Casolari Moreno.** *Chrysis aequinottialis* (sic!), 1, 34, 75, 1 (box 51).

##### Remarks.

Dahlbom (1854) described *Chrysis aequinoctialis* based on at least a series of specimens. In the Spinola collection, only a male of this species is currently housed. Bohart pinned a lectotype label under this specimen, but the lectotype designation has not been published. Kimsey and Bohart (1991: 425) placed *Chrysis aequinoctialis* in synonymy with *Chrysis intricata* Brullé, 1846 and reported "Syntype male, female; Brazil (Turin)". However, the female syntype is not preserved in the collection. Since the female is possibly lost and the above male of *Chrysis aequinoctialis* corresponds to Dahlbom’s species description, we here designate the male specimen as lectotype of *Chrysis aequinoctialis*. We deem Spinola’s collection as the right place for this lectotype designation, since the collection is cited in the original description and the choice of the male is to be preferred since the genitalia dissection can help in a future study of *Chrysis aequinoctialis*.

*Chrysis aequinoctialis* is currently considered as synonym of *Chrysis intricata* Brullé, 1846 (Kimsey and Bohart 1991). Yet the interocellar distance measured between lateral ocelli (IOD), the length of the malar space and the transversal frontal carina (TFC) are distinctly different between the lectotype of *Chrysis aequinoctialis* and those specimens described by Bohart and Kimsey (1982: 135). It belongs to the *smaragdula* group and a worldwide revision this group is needed.

##### Current status.

*Chrysis intricata* Brullé, 1846 (synonymised by Kimsey and Bohart 1991: 425).

**Plate 1.**
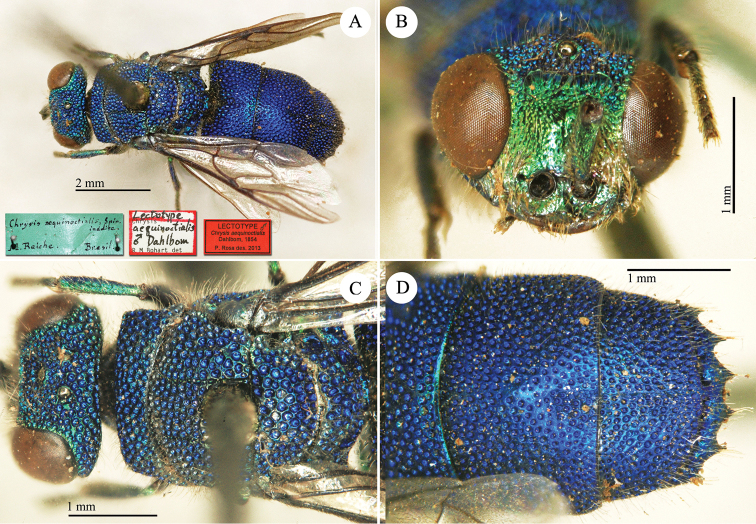
*Chrysis aequinoctialis* Dahlbom, lectotype. **A** Habitus, dorsal view **B** head, frontal view **C** head and mesosoma, dorsal view **D** metasoma, dorsal view.

#### Chrysis alternans

Taxon classificationAnimaliaHymenopteraChrysididae

Dahlbom, 1854

Chrysis alternans : Dahlbom 1854: 236.

##### Type locality.

"Habitat in Aegypto et in Promontorio bonae spei, Museis DD. Drewsen, Spinola et Westermann".

##### Material.

**Paralectotype** 1 ♂. *Chrysis* vel *Poecilochroa alternans* Klug. cum var. *β* D. Draege Cap. B. Esp.

**Paralectotypes** 2 ♀♀. Idem.

**Catalogue Casolari & Casolari Moreno.** *Chrysis alternans*, 13, 53, 21, 3 (box 51).

**Paralectotype** 1 ♂. *Poecilochroa alternans*, Klug.

**Catalogue Casolari & Casolari Moreno.** *Poecilochroa bifasciata*, 132, 53, 0, 1 (box 52).

##### Remarks.

Dahlbom (1854) described three variations (*a*, *b*, *c*) of *Chrysis alternans*. Bohart (in Kimsey and Bohart 1991: 381) designated a lectotype of *Chrysis alternans* at ZMUC. Dahlbom’s three variations of *Chrysis alternans* are nowadays considered distinct species, referring to two different species groups. In Spinola’s box 51, there are three specimens placed under the label *Chrysis alternans* (Casolari and Casolari Moreno 1980: 79). Two of them belong to var. *a*, whereas the third refers to var. *b* and belongs to a different species with a similar colouration. A further specimen of "*Chrysis alternans*" was found in box 52, under the name *Poecilochroa alternans* and should be considered as another paralectotype referring to var. *b*. The examination of the lectotype in ZMUC revealed that it belongs to var. *a* (Madl and Rosa 2012: 16). It belongs to the *alternans* group.

##### Current status.

*Chrysis alternans* Dahlbom, 1854.

#### Chrysis analis

Taxon classificationAnimaliaHymenopteraChrysididae

Spinola, 1808

Plate 2

Chrysis analis : Spinola 1808: 26.

##### Type locality.

"Liguria".

##### Material.

**Lectotype** (here designated) ♂. *Chrysis analis* Spin. Ins. Lig. - non Meg.[erle] D. Ghiliani Genes [Genova] Espagne.

**Paralectotype** 1 ♂. Idem.

**Catalogue Casolari & Casolari Moreno.** *Chrysis analis*, 1, 110/101, 33, 5 (box 51).

##### Remarks.

Spinola (1808) described *Chrysis analis* based on a series of specimens: "*Habitat passim in Liguria, haud infrequens*". Today there are six specimens in the Spinola collection that refer to *Chrysis analis*, even though Casolari and Casolari (1980: 79) listed only five. One specimen bears a rounded label that was likely acquired after the description of the species. Two other specimens bear handwritten labels. However, the handwriting is not by Spinola and thus the specimens cannot be considered as type material; they were very likely collected by Ghiliani in Spain and later sent to Spinola, as reported on the white label at the base of the series. One specimen is a male of *Chrysis splendidula* Rossi. The remaining two specimens were collected at Genoa, as reported on the main underlying white label. Since there are different species found in the type series, we designate as lectotype of *Chrysis analis* Spinola an undamaged male that matches Spinola’s description of this species and the current interpretation of the species by most currently active authors. It belongs to the *comparata*-*scutellaris* group.

##### Current status.

*Chrysis analis* Spinola, 1808.

**Plate 2.**
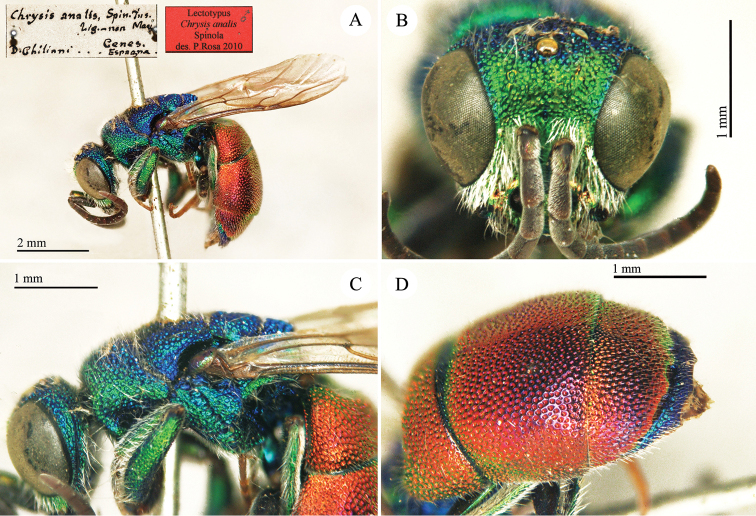
*Chrysis analis* Spinola, lectotype. **A** Habitus, lateral view **B** head, frontal view **C** head and mesosoma, lateral view **D** metasoma, dorso-lateral view.

#### Chrysis armena

Taxon classificationAnimaliaHymenopteraChrysididae

Dahlbom, 1854

Chrysis armena : Dahlom 1854: 274.

##### Type locality.

"Habitat in Armenia, a D. Osculati detecta, Mus. D. Spinola".

##### Material.

**Holotype** ♂. *Chrysis armena* Spin. D. Osculati Arménie.

**Catalogue Casolari & Casolari Moreno.** *Chrysis armena*, 1, 22, 65, 1 (box 51).

##### Remarks.

It belongs to the *pallidicornis* group.

##### Current status.

*Chrysis pallidicornis* Spinola, 1838 (synonymised by Mocsáry 1887: 13).

#### Chrysis assimilis

Taxon classificationAnimaliaHymenopteraChrysididae

Dahlbom, 1854

Plate 3

Chrysis assimilis : Dahlbom 1854: 201.

##### Type locality.

"Habitat in Sicilia et Aegypto. Tria specimina lustravi: unum a D. Grohmann in Sicilia lectum, Mus. Vienn. teste D. Kollar; alterum e Stansnio a D. Loew communicatum, tertium ex Aegypto D. Walt, Mus. D. Spinola".

##### Material.

**Lectotype** (here designated) ♀. *Chrysis assimilis* Spin. D. Waltl. Égypte.

**Catalogue Casolari & Casolari Moreno.** *Chrysis assimilis*, 1, 23, 95, 1 (box 51).

##### Remarks.

Dahlbom (1854) described *Chrysis assimilis* based on three syntypes. The Egyptian syntype is still in Spinola’s collection. The second syntype is apparently lost. The third syntype from Sicily is housed in NHMW. *Chrysis assimilis* was considered as a valid species by different authors: Abeille (1879: 41), Gogorza (1887: 51), De Stefani (1888: 138), du Buysson (in André), 1893: 233). However, after publication of Mocsáry’s seminal monograph on cuckoo wasps (1889: 183), *Chrysis assimilis* was considered by most authors as a synonym of *Chrysis pumila* Klug, 1845.

Mocsáry (1889) placed all the known species relating to the genus *Chrysidea* Bischoff in synonym with *Chrysis pumila* (*Chrysis assimilis* Dahlbom, *Chrysis virgo* Abeille, *Chrysis tarsata* Tournier, and *Chrysis persica* Radoszkowski), and most authors followed this interpretation (Dalla Torre 1892: 37; Bischoff 1913: 35; Trautmann 1927: 102; Berland and Bernard 1938: 70; Linsenmaier 1951: 62; Balthasar 1953: 170).

Linsenmaier (1959: 171) recognized different species and subspecies of *Chrysidea* in Europe and the Mediterranean region and he listed characteristics to identify them. He treated *Chrysidea* Bischoff as a subgenus of *Chrysis* Linnaeus and considered *Chrysis pumila* Klug and *Chrysis persica* Radoszkowski as valid species, speculated about *Chrysis assimilis* Dahlbom, 1854, possibly being a synonym of *Chrysis persica*, and he described a new subspecies Chrysis pumila: ssp. disclusa. A few years later, Linsenmaier (1987: 155) stated that *Chrysis pumila* *sensu* auctorum does not occur at the species’ typical locality and consequently suggested synonymizing *Chrysis persica* and *Chrysis pumila*. He described the species previously named *Chrysis pumila* as Chrysis (Trichrysis) pumilionis n. sp. Linsenmaier (1987: 155) and also assigned it the subspecies *disclusa* Linsenmaier, 1959. This combination is erroneous for nomenclatural reasons, and the name *Chrysis disclusa* obviously has date priority over *Chrysis pumilionis*, as already stated by Niehuis (2001: 122). Currently, the species belonging to the *Chrysis pumila* group are included in the genus *Chrysidea* Bischoff, 1910 (Kimsey and Bohart 1991).

We examined both available syntypes of *Chrysis assimilis* Dahlbom. They belong to two different species: the Egyptian specimen housed in MRSN is a female of *Chrysis persica* (*sensu* Linsenmaier 1959 = *Chrysis pumila* *sensu* Linsenmaier 1987); the Sicilian specimen housed in the NHMW is a male of *Chrysis pumila disclusa* (*sensu* Linsenmaier 1959 = *Chrysis pumilionis disclusa* *sensu* Linsenmaier 1987).

Since there is no stability in the species names of the genus *Chrysidea*, we propose to designate a neotype of *Chrysis pumila* Klug, 1845 and a lectotype of *Chrysis assimilis* Dahlbom, 1854.

As neotype of *Chrysis pumila* Klug, 1845, we designate a male housed in Linsenmaier’s collection. The type locality of *Chrysis pumila* is Ambukhol, once being in Egypt, nowadays in North Sudan. Since there are no available specimens from the type locality in any visited European museum, the specimen selected as neotype was collected in the closest locality to Ambukhol know to us. Specifically, it was collected in Egypt and bears the following locality label: Aegypten Fayoum H. Suster 9.1948 Coll. Linsenmaier. The designation of this specimen as neotype of *Chrysis pumila* retains Linsenmaier’s interpretation (1987 and following papers) of the species and that most European Hymenopterists adopted. We agree with Linsenmaier’s interpretation of the species, since *Chrysis pumila* *sensu* Linsenmaier (1959) seems to be restricted in its occurrence to central and southern Europe. Mocsáry (1909: 406) was the last author to examine Klug’s type of *Chrysis pumila* in MNHU. We searched for this type together the curator, Dr. Frank Koch, and his assistant, Viola Richter, at the MNHU, but in vain. All taxa deposited at MNHU are registered with an index card that includes information on the type status of each specimen. Klug’s types are registered with a red "T" [Type], except in the case of *pumila*, which is marked with a "T" written with drawing pencil. The type possibly had been lost already during compilation of the index cards.

As lectotype of *Chrysis assimilis* Dahlbom, 1854, we designate a specimen housed in the Spinola collection labelled: *Chrysis assimilis* Spin. D. Waltl Égypte. This designation retains the synonymy of *Chrysis assimilis* Dahlbom, 1854 and *Chrysis pumila* Klug, 1845. In fact, a lectotype designation based on the Sicilian type specimen housed at NHMW would have caused nomenclatorial stability in the genus *Chrysidea* since *Chrysis assimilis* is the second available name described.

In Europe and in the Mediterranean region, there are currently four known species and one subspecies of *Chrysidea*: *Chrysis asensioi* Mingo, 1985 (distribution: Spain, south France, north Italy, Greece); *Chrysis disclusa* Linsenmaier, 1959 (Spain, south France, Italy, Sicily, north Africa); *Chrysis pumila* Klug, 1845 (Transpalaearctic: from the Iberian peninsula and northern Africa to China; Afrotropical); *Chrysis disclusa pumilionis* Linsenmaier, 1987 (Austria, Croatia, Czech Republic, France, Germany, Greece, Hungary, Macedonia, Morocco, Spain, Switzerland); *Chrysis rebecca* Morice, 1909 (Palestine, Syria).

##### Current status.

*Chrysidea pumila* (Klug, 1845) (synonymised by Mocsary 1887: 13; and transferred by Kimsey and Bohart 1991: 314).

**Plate 3.**
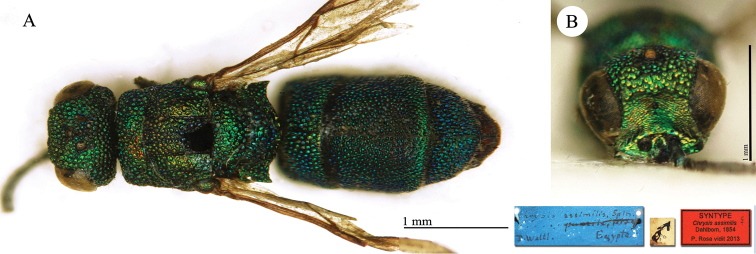
*Chrysis assimilis* Dahlbom, lectotype **A** Habitus, dorsal view **B** head, frontal view.

#### Chrysis basalis

Taxon classificationAnimaliaHymenopteraChrysididae

Dahlbom, 1854

Plate 4

Chrysis basalis : Dahlbom 1854: 106.

##### Type locality.

"Habitat in Algeria; D. Rambur, Mus. Spinolae".

##### Material.

**Holotype** ♂. *Chrysis basalis* Dlbm. D. Rambur Algérie.

**Catalogue Casolari & Casolari Moreno.** *Chrysis basalis*, 1, 154, 23, 1 (box 50).

##### Remarks.

It belongs to the *Chrysis millenaris* group.

##### Current status.

*Chrysis basalis* Dahlbom, 1854.

**Plate 4.**
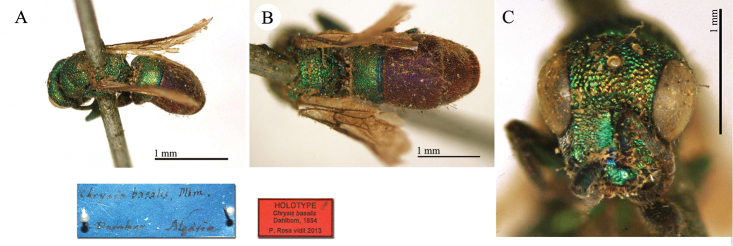
*Chrysis basalis* Dahlbom, holotype **A** Habitus, dorso-lateral view **B** mesosoma and metasoma, dorsal view **C** head, frontal view.

#### Chrysis bihamata

Taxon classificationAnimaliaHymenopteraChrysididae

Spinola, 1838

Plate 5

Chrysis bihamata : Spinola 1838: 450.

##### Type locality.

"Egypte".

##### Material.

**Lectotype** (here designated) ♀. *Chrysis bihamata* Spin. D. Waltl Égypte.

**Paralectotype** 1 ♀. idem.

**Catalogue Casolari & Casolari Moreno.** *Chrysis bihamata*, 1, 23, 95, 2 (box 51).

##### Remarks.

There are two syntypes of *Chrysis bihamata* in the Spinola collection. The first syntype was heavily damaged by a dermestid attack and it is almost unrecognizable. Therefore, we designate the second specimen, currently being in good condition, as lectotype. It is only partially covered by an old mould layer. *Chrysis bihamata* is the first species described in the *Chrysis bihamata* group.

##### Current status.

*Chrysis bihamata* Spinola, 1838.

**Plate 5.**
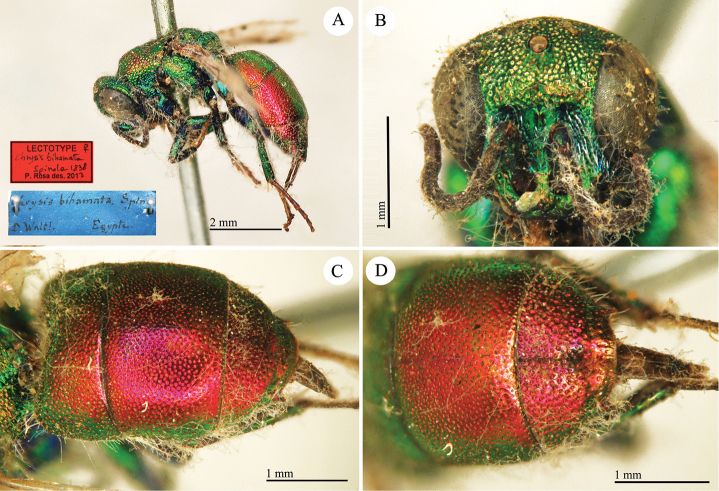
*Chrysis bihamata* Spinola, lectotype. **A** Habitus, lateral view **B** head, frontal view **C** metasoma, dorso-lateral view **D** second to third metasomal tergites, dorsal view.

#### Chrysis carinata

Taxon classificationAnimaliaHymenopteraChrysididae

Dahlbom, 1854

Chrysis carinata : Dahlbom 1854: 167 *nec* Block, 1799.

##### Type locality.

"Habitat in Africa, ad Cap. bon. sp. a D. Draege detecta; Mus. Spinolae".

##### Material.

**Holotype** (sex unknown): *Chrysis carinata* / (*Nemophora*) Draege / D. Draege Cap. B. Esp.

**Catalogue Casolari & Casolari Moreno.** *Chrysis carinata*, 45, 53, 21, 1 (box 50).

##### Remarks.

In the original description, Dahlbom (1854: 168) listed also the name *Nemophora carinata* Draege, the same name found on the label pinned with the specimen in the Spinola collection. The type is seriously damaged, only its mesosoma exists. It belongs to the *Chrysis oxygona* group.

##### Current status.

*Chrysis capensis* Mocsáry (1887: 14), replacement name for *Chrysis carinata* Dahlbom, 1854 (*nec* Block, 1799).

#### Chrysis chilensis

Taxon classificationAnimaliaHymenopteraChrysididae

Spinola, 1851

Plate 6

Chrysis chilensis : Spinola 1851: 404.

##### Type locality.

Chile "Esta especie es bastante comun en Chile y principalmente en las cercanias de Coquimbo, Illapel, etc.".

##### Material.

**Lectotype** (here designated) ♂. *Chrysis chiliensis* (sic!), Spin. / M. Gay / Chili.

**Paralectotypes** 2♀♀. idem.

**Catalogue Casolari & Casolari Moreno.** *Chrysis chiliensis*, 1, 52, 32, 3 (box 51).

##### Remarks.

*Chrysis chilensis* was described based on a syntype series that included males and females. Other syntypes are housed in LZM (Dahlbom’s collection) and MNHN (du Buysson 1898: 166).

Gay (1851: 404) erroneously assumed that *Chrysis chilensis* Spinola was described in 1845 in Atlas Zoologico, Entomologia, Himenópteros, tav. 4, fig. 6. The same author therefore also considered *Chrysis chilensis* a senior subjective synonym of *Chrysis grandis* Brullé, 1846. Currently, *Chrysis chilensis* Spinola, 1851 is considered a junior subjective synonym of *Chrysis grandis* Brullé, 1846. Since we could not find the reference "Atlas Zoologico" cited by Gay, we consider *Chrysis chilensis* Spinola, 1851 as the junior synonym of *Chrysis grandis* Brullé, 1846. We designate the male specimen, still being in good condition, as the lectotype. It belongs to the *Chrysis grandis* group.

##### Current status.

*Chrysis grandis* Brullé, 1846 (synonymised by Mocsáry 1889: 404).

**Plate 6.**
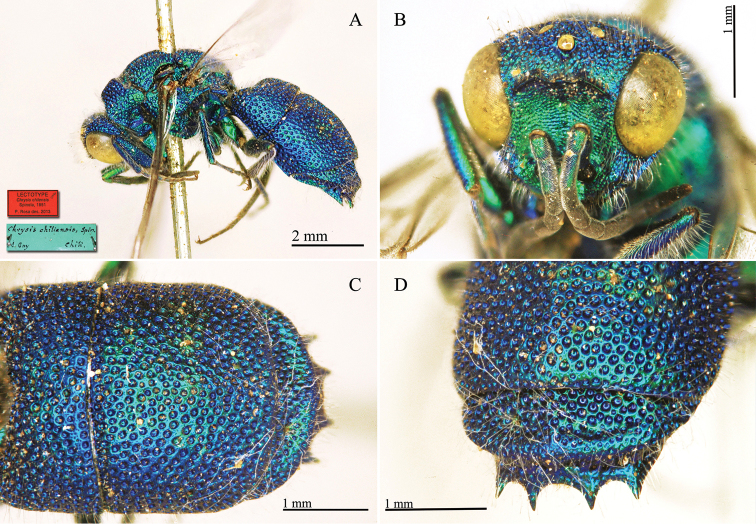
*Chrysis chilensis* Spinola, lectotype. **A** Habitus, lateral view **B** head, frontal view **C** metasoma, dorsal view **D** second and third metasomal tergites, dorso-lateral view.

#### Chrysis comparata

Taxon classificationAnimaliaHymenopteraChrysididae

Lepeletier, 1806

Plates 7 , 8

Chrysis comparata : Lepeletier 1806: 127.

##### Type locality.

France "Meudon".

##### Material.

**Syntypes** 2♂♂. *Chrysis comparata*, Lepell. / D. De St. Fargeau, Paris.

**Neotype** (here designated) ♀. Sicile et Toscane / **Lectotype** ♀ *Chrysis distinguenda* Dhlb. P. Rosa des. 2012 / **Neotypus** *Chrysis comparata* Lepeletier des. P. Rosa 2013.

**Catalogue Casolari & Casolari Moreno.** *Chrysis comparata*, 148, 185, 53, 3 (box 51).

##### Remarks.

In the Spinola collection, there are two identical specimens of *Chrysis comparata* according to their general habitus, preparation and state of conservation. The type is likely housed in the Spinola collection, given that both Dahlbom (1854: 284) and Abeille (1879: 69) examined it there. Kimsey and Bohart (1991: 399) listed syntype males and they assumed to be housed in MNHN. However, they refer to some specimens cited by du Buysson (1898: 166) not included to the type series. In any case, the type of *Chrysis comparata* is not housed in MNHN (du Buysson 1899) and was not found during our research.

It is evident that Lepeletier’s description and drawing (Tav.1, fig.12) of *Chrysis comparata* does not match the current interpretation of the species. It rather perfectly matches the description of the male of *Chrysis pyrophana* Dahlbom, 1854. No other European species, in comparison, has such small body length, colour of head, mesosoma and the anterior part of T-I green-blue combined with the anal margin with four blunt and weakly developed teeth. The interpretation of *Chrysis comparata* (= male of *Chrysis pyrophana*) was clear to Dahlbom (1854), Mocsáry (1889: 431) and Dalla Torre (1892: 52). Dahlbom (1854: 284) described the female under the name of *Chrysis pyrophana* Dahlbom, 1854, because of the remarkable sexual dimorphism.

Dahlbom (1854: 282) described *Chrysis comparata* in the modern sense under the name *Chrysis distinguenda* Dahlbom (*nec* Spinola, 1838). Mocsáry (1879: 10) recognized the homonymy with Spinola’s *Chrysis distinguenda* and replaced the name *Chrysis distinguenda* Dahlbom with the name *Chrysis chevrieri* Mocsáry (*nec* Abeille, 1877). This name remained in use for a long time in European collections.

The first wrong interpretation of this taxon dates back to Abeille (1879). His incorrect interpretation was already recognized by Mocsáry (1887: 14; 1889: 479), who included *Chrysis comparata* *sensu* Abeille in the synonymic list of *Chrysis chevrieri* Mocsáry, 1879. Please note that Abeille (1879) additionally independently described the male of *Chrysis pyrophana* Dahlbom, 1854 under the name *Chrysis insoluta*.

Mocsáry (1889: 431) correctly recognized the synonymy *Chrysis insoluta* Abeille = *Chrysis comparata* Lepeletier. Later, Bischoff (1913: 49) interpreted the name *comparata* Lepeletier as referring to females of *Chrysis distinguenda* Dahlbom and *Chrysis chevrieri* Mocsáry as well as the male of *pyrophana* (= *insoluta* Abeille).

The current (mis-) interpretation of the species *Chrysis comparata* was anchored in the taxonomic literature by Trautmann (1927: 154). His *Chrysis comparata* is a large and robust species, with the entire metasoma golden, without the typical green-blue. Berland and Bernard (1938: 113) followed this interpretation, and so did Linsenmaier (1959: 148).

According to the Principle of Priority, *Chrysis pyrophana* Dahlbom, 1854 should be named *Chrysis comparata* Lepeletier, 1806 and the species currently identified as *Chrysis comparata* Lepeletier should be named *Chrysis miegii* Guérin, 1842, the first available name for this species. However, this change would compromise the nomenclatural stability, since the name *Chrysis comparata* was recognized as a valid name for one of the most common European species by nearly all the authors in the last fifty years.

As already observed by Abeille (1879), the Spinola collection houses the type of *Chrysis distinguenda* Dahlbom. This name was considered as a synonym of *Chrysis comparata* *sensu* auctorum. To preserve the stability of nomenclature, we designate the neotype of *Chrysis comparata* Lepeletier (Plate 8) based on a syntype ♀ of *Chrysis distinguenda* Dahlbom (labels: Sicile et Toscane / **Lectotype** ♀ *Chrysis distinguenda* Dhlb. P. Rosa des. 2012 / **Neotypus** *Chrysis comparata* Lepeletier des. P. Rosa 2013), in accordance with Art. 75.6 of the Code: Conservation of prevailing usage by a neotype. The Article states that when an author discovers that the existing name-bearing type of a nominal species-group taxon is not in taxonomic accord with the prevailing usage of names and stability or universality is threatened thereby, he should maintain prevailing usage [Art. 82] and request the Commission to set aside under its plenary power [Art. 81] the existing name-bearing type and designate a neotype. It belongs to the *comparata*-*scutellaris* group.

##### Current status.

*Chrysis comparata* Lepeletier, 1806.

**Plate 7.**
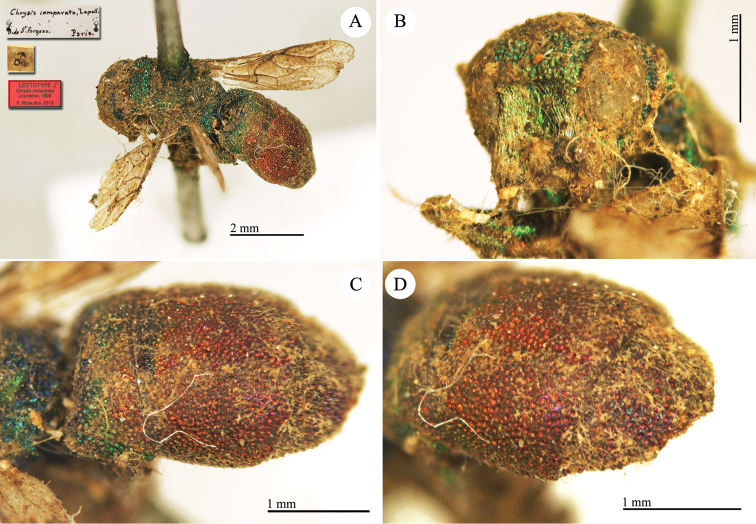
*Chrysis comparata* Lepeletier, lectotype. **A** Habitus, dorsal view **B** head, frontal view **C** metasoma, dorso-lateral view **D** second and third metasomal tergites, dorso-lateral view.

**Plate 8.**
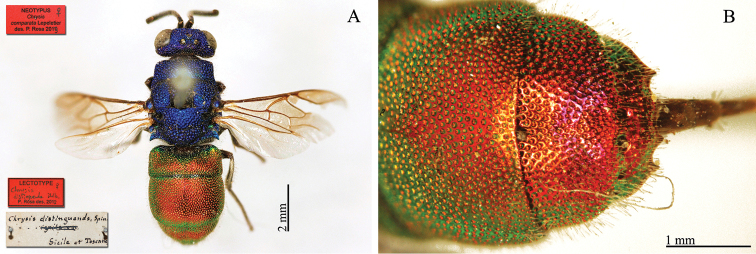
*Chrysis distinguenda* Dahlbom, lectotype; *Chrysis comparata* Lepeletier, neotype **A** Habitus, dorsal view **B** second and third metasomal tergites, dorsal view.

#### Chrysis cuprea

Taxon classificationAnimaliaHymenopteraChrysididae

Brullé, 1846

Chrysis cuprea : Brullé 1846: 40 *nec* Rossi, 1790.

##### Type locality.

"Hab.[itat] le Cap de Bonne-Espérance. Collect. de M. Serville".

##### Material.

**Holotype** ♀. *Chrysis cuprea* Brullé / Coll. Serv.[ille], Cap B.[one] Esp.[érance].

**Catalogue Casolari & Casolari Moreno.** *Chrysis cuprea*, 13, 53, 79, 1 (box 51).

##### Remarks.

The type is badly damaged, only the mesosoma remains.

##### Current status.

*Chrysis mutata* Mocsáry, 1882 (Mocsáry 1882: 50), replacement name for *Chrysis cuprea* Brullé 1846.

#### Chrysis dichroa

Taxon classificationAnimaliaHymenopteraChrysididae

Dahlbom, 1854

Plate 9

Chrysis dichroa : Dahlbom 1854: 146.

##### Type locality.

"Habitat in Austria D. D. Klug, Kollar, Parreys et Spinola; in Italia ad Buda, D. Zeller; in Asia minori ad Ephesum mense Aprili 1842, D. Loew".

##### Material.

**Lectotype** (here designated) ♀. *Chrysis dichroa*, Klug / D. Parreyss, Dalmatia // Buda.

**Catalogue Casolari & Casolari Moreno.** *Chrysis dichroa* 132, 81, 68, 3 (box 50).

##### Remarks.

Dahlbom (1854) described *Chrysis dichroa* based on a syntypic series of specimens collected in Austria, Hungary (Buda, not Italy) and Turkey (Ephesus) lent by Klug, Kollár, Parreys, Spinola, Zeller, and Loew. The female syntype housed in the Spinola collection matches the current interpretation of the species and we designate it as a lectotype. The designation is necessary because during the last five decades, Linsenmaier (1959, 1968) and Arens (2001) described numerous species of the *Chrysis dichroa* group collected in the Eastern Mediterranean region. Therefore, there is the possibility that syntypes of *Chrysis dichroa* from Turkey could now belong to a different species. It belongs to the *Chrysis dichroa* group.

##### Current status.

*Chrysura dichroa* (Dahlbom, 1854) (transferred by Kimsey and Bohart 1991: 488).

**Plate 9.**
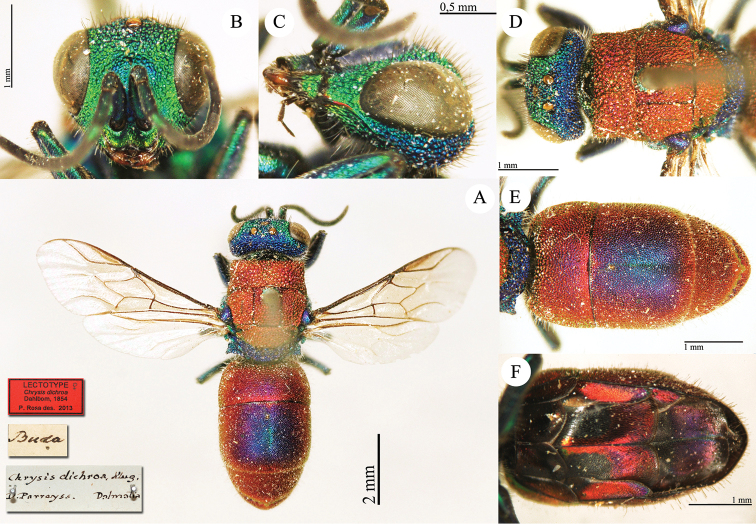
*Chrysis dichroa* Dahlbom, lectotype. **A** Habitus, dorsal view **B** head, frontal view **C** head, lateral view **D** head and mesosoma, dorsal view **E** metasoma, dorsal view **F** metasoma, ventral view.

#### Chrysis distinctissima

Taxon classificationAnimaliaHymenopteraChrysididae

Dahlbom, 1854

Chrysis distinctissima : Dahlbom 1854: 211.

##### Type locality.

"Habitat in America meridionali".

##### Material.

**Syntype** 1 ♂. *Chrysis distinctissima* / Dlbm. - *Chrysis fasciata*, m. / D. Buquet, Cayenna // **Holotype** ♂. *Chrysis intricans* Spinola, R. M. Bohart det. // **Lectotype** ♂. *Chrysis distinctissima* Dahlbom, R. M. Bohart det.

**Catalogue Casolari & Casolari Moreno.** *Chrysis distinctissima*, 27, 56, 1, 2 (box 51).

##### Remarks.

Spinola (1840: 202) gave the diagnosis of a male belonging to *Chrysis fasciata* Fabricius, 1804 (*nec* Olivier, 1790), collected at Cayenna in French Guyana by Leprieur and received via Buquet. In the same diagnosis, Spinola (1840: 203) described *Chrysis intricans* Spinola, 1840 ("*n. sp.*?", giving a complete description), based on a second specimen: "*Celle-ci ne fait pas partie des récoltes de M. Leprieur, et elle est étrangère au sujet de ce Mémoire; je me contenterai d’en constater l’existance, et de signaler les traits qui la distinguent de notre n°50*". Therefore, there should be theoretically both a specimen of *Chrysis fasciata* Fabricius *sensu* Spinola as well as a holotype of *Chrysis intricans* Spinola in the Spinola collection.

Dahlbom (1854: 211) described *Chrysis distinctissima* based on one specimen examined in 1847 at LZM and on a second specimen loaned by Spinola under the name *Chrysis intricans*. He considered as *Chrysis distinctissima* also *Chrysis fasciata* Spinola (*nec* Fabricius). In particular, Dahlbom listed in the type series: "*Chrysis fasciata* Spinol. Annal. Ent. 1840. 202: 50"; then the specimen from Lund; lastly the specimen known as *Chrysis intricans*" Mus. Spinolae", without further remarks. Two pages after, Dahlbom (1854: 213) listed under the name *Chrysis coerulans* Fabricius a second specimen from the Spinola collection named *Chrysis intricans* collected at Cayenne and received by Buquet. This specimen should correspond to the holotype of *Chrysis fasciata* Spinola (*nec* Fabricius). The Spinola collection does not house any sample labelled "*Chrysis intricans*", but it houses two specimens label "*Chrysis distinctissima* Dlbm – *Chrysis fasciata* m.[ihi]". The first is severly damaged, having no metasoma. The second is in good condition. The first was labelled by Bohart as holotype of *Chrysis intricans* and lectotype of *Chrysis distinctissima*; the second specimen was labelled by Bohart as *Chrysis excavata* Brullé, 1846. The lectotype designation of *Chrysis distinctissima* has not been published. In Dahlbom’s collection in LZM, there are no labels referring to *Chrysis distinctissima* Dahlbom and there is only one specimen lablled *Chrysis fasciata* Fabricius. Unfortunately, we currently cannot confidently infer which specimen could be the type of one species and which one could be the type of the second species, since Spinola’s descriptions are ambiguous. According to Kimsey and Boahrt (1991) it belongs to the *Chrysis intricans* group.

##### Current status.

*Chrysis distinctissima* Dahlbom, 1854.

#### Chrysis distinguenda

Taxon classificationAnimaliaHymenopteraChrysididae

Dahlbom, 1854

Plate 8

Chrysis distinguenda : Dahlbom 1854: 282 *nec* Spinola, 1838.

##### Material.

**Lectotype** (here designated) ♀. *Chrysis distinguenda*, Spin.; Sicile et Toscane.

**Paralectotypes** 3 ♀♀. idem.

**Catalogue Casolari & Casolari Moreno.** *Chrysis distinguenda*, 1, 204/96, 0, 4 e 0, 96, 85, 3 (box 51).

**Paralectotype** 1 ♀. *Chrysis distinguenda*, 1 ♀ aut potius *Chrysis ignita*, var. *a* ?; D. de Sanvitali. Toscana.

**Catalogue Casolari & Casolari Moreno.** *Chrysis distinguenda* 0, 96, 85, 3 (box 51).

##### Remarks.

Dahlbom (1854: 282) described *Chrysis distinguenda* Dahlbom (*nec* Spinola, 1838) based on a unspecified number of specimens housed in the Spinola collection. He knew that Spinola did not include these specimens in his description of *Chrysis distinguenda* Spinola, 1838. In fact, Dahlbom explicitely wrote: "*Chrysis distinguenda Mus. Spinolae (non Annales Entomol. 1838. 450: VII, quae species est toto corpore cyaneo-viridis)".* Dahlbom (1854) gave a new accurate description of *Chrysis distinguenda* Dahlbom, more than two pages with some drawings. Mocsáry (1879, 1889) realized that *Chrysis distinguenda* Dahlbom a synonym of *comparata* *sensu* auctorum, and that *Chrysis distinguenda* Spinola most likely represented a separate and unknown species, the type of which apparently has been lost. Mocsáry (1879) replaced the name *Chrysis distinguenda* Dahlbom, 1854 (*nec* Spinola, 1838) with *Chrysis chevrieri* nec Abeille, 1879. Finally, Linsenmaier (1999: 221) considered *Chrysis distinguenda* Spinola as a separate species belonging to the *Chrysis ignita* group, although he seemingly never studied type specimens in Spinola’s collection.

We consider five specimens in the Spinola collection as syntypes of *Chrysis distinguenda* Dahlbom, 1854. In box 51, there are two main labels bearing the name *Chrysis distinguenda*. Under the second label, there are three specimens: the last two are males of *Chrysis comta* Förster, 1853 and are likely the specimens listed by Spinola as *Chrysis ignita* var. *a* on the label. We select one female syntype of *Chrysis distinguenda* Dahlbom as neotype of *Chrysis comparata* Lepeletier (Plate 8) (labels: Sicile et Toscane / **Lectotype** ♀ *Chrysis distinguenda* Dhlb. P. Rosa des. 2012 / **Neotypus** *Chrysis comparata* Lepeletier des. P. Rosa 2013). For further notes see remarks under *Chrysis comparata* Lepeletier. The type of *Chrysis distinguenda* Spinola, 1838 is currently lost (see the chapter II Types not housed in the Spinola collection).

##### Current status.

*Chrysis comparata* Lepeletier, 1806 (synonymised by Trautmann 1927: 154).

#### Chrysis dives

Taxon classificationAnimaliaHymenopteraChrysididae

Dahlbom, 1854

Plates 10 , 11

Chrysis dives : Dahlbom 1854: 301 *necChrysis dives* Lucas, 1849.

##### Type locality.

"Habitat in Sicilia, D. Grohmann; Mus. D. Spinola."

##### Material.

**Holotype** ♀. *Chrysis dives* Kl. M. Ber. / D. Grohmann, Sicile.

**Neotype** (here designated): ♀ Rákospalota [= Budapest] 23.V.1879 leg. Biró.

**Catalogue Casolari & Casolari Moreno.** *Chrysis dives*, 141, 204, 34, 1 (box 51).

##### Remarks.

The type of *Chrysis dives* is badly damaged, lacking its head and metasoma. Even if only the mesosoma had remained, it is clear that the current interpretation of the species does not match the type. The mesosoma clearly refers to a species that is well known under the name *Chrysis varidens* Abeille, 1878. Dahlbom (1854: 300) considered *Chrysis dives* as the female (var. *b*) of *Chrysis pulchella* Spinola, 1808, till then known only from a single male. But on the following page of his description of *Chrysis dives* (1854: 301), Dahlbom described *Chrysis dives* as a valid species, not as a variation of *Chrysis pulchella*. The description does not match the current interpretation of the species. The females of *Chrysis pulchella* and of *Chrysis calimorpha* Mocsáry, 1882 *sensu* auctorum (replacement name for *Chrysis dives* Dahlbom (*nec* Lucas, 1849)) have the same size and the shape as conspecific males; they differ from each other by their punctuation on the head and mesosoma, (Rosa 2005, 2006).

Mocsáry (1882: 71, 90) observed that the name *Chrysis dives* Dahlbom, 1854 is a junior homonym of *Chrysis dives* Lucas, 1849. Therefore, he replaced the name *Chrysis dives* Dahlbom with *Chrysis calimorpha* Mocsáry. In the same article, Mocsáry (1882: 71) listed two specimens of *Chrysis calimorpha* collected in central Hungary. Later, Móczár (1965: 179) designated these two specimens as lecto- and paralectotype of *Chrysis calimorpha*. These two Hungarian specimens do not belong to the type series, because *Chrysis calimorpha* is the replacement name of *Chrysis dives*. The only holotype by monotypy of this species is the Sicilian specimen housed in the Spinola collection. According to Art. 74.2 of the Code when it is demonstrated that a specimen is not a syntype, it automatically loses its status of lectotype. Móczár's lectotype is consequently set aside, since the selected specimen is not a syntype.

Given the holotype of *Chrysis dives* Dahlbom is almost destroyed, the mesosoma clearly belongs to a different species relative to the current interpretation of *Chrysis calimorpha* *sensu* auctorum, and because the history of the name *calimorpha* is complex, we will ask for the suppression of the type of *Chrysis dives* to the Commission on the ICZN. We designate as neotype of *Chrysis dives* the female of *Chrysis calimorpha* Mocsáry, selected by Móczár as lectotype and bearing the following labels: Rákospalota [= Budapest] 23.V.1879 leg. Biró (Plate 11). It belongs to the *Chrysis pulchella* group.

##### Current status.

*Chrysis calimorpha* Mocsáry, 1882 (Mocsáry 1882: 71), replacement name for *Chrysis dives* Dahlbom 1854.

**Plate 10.**
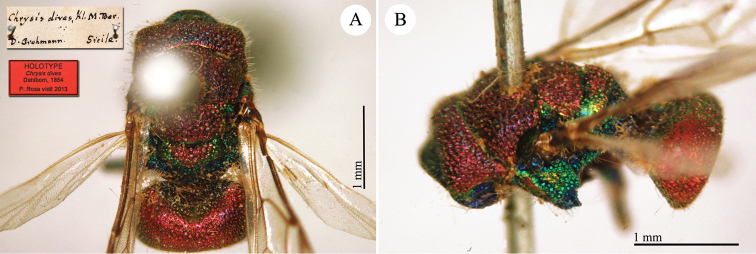
*Chrysis dives* Dahlbom, holotype **A** Mesosoma and metasoma, dorsal view **B** mesosoma and metasoma, dorso-lateral view.

**Plate 11.**
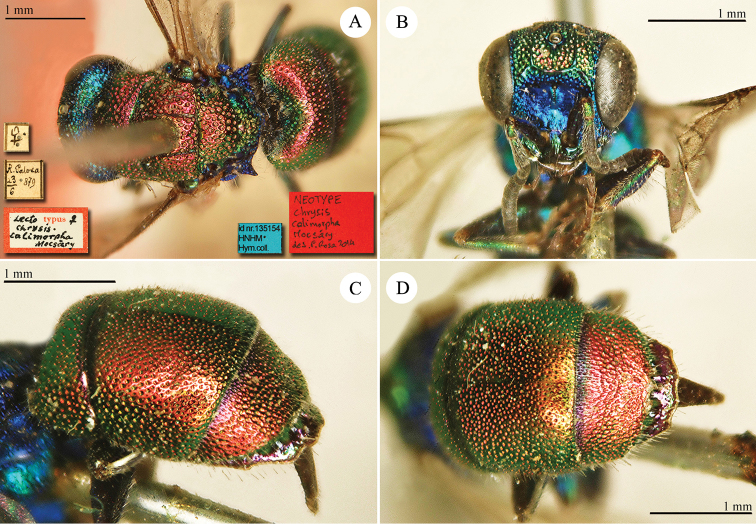
*Chrysis calimorpha* Mocsáry, neotype. **A** Habitus, dorsal view **B** head, frontal view **C** metasoma, lateral view **D** second and third metasomal tergites, dorsal view.

#### Chrysis elegans

Taxon classificationAnimaliaHymenopteraChrysididae

Lepeletier, 1806

Chrysis elegans : Lepeletier 1806: 128.

##### Type locality.

unknown.

##### Material.

**Holotype** (?) ♀. *Chrysis elegans*, Lepell. et Dlbm. var. *a*, ♀. - *β*. id. var. *ab* / Dlbm. - *Chrysis sicula*, mihi olim / D. Grohmann. Sicilia.

**Catalogue Casolari & Casolari Moreno.** *Chrysis elegans*, 164, 204, 34, 2 (box 50).

##### Remarks.

Two specimens are found under the label *Chrysis elegans* in the Spinola collection. One bears a single label (*β*) and likely refers to *Chrysis sicula* Dahlbom (see under the name *sicula*). The second specimen could be the holotype of *Chrysis elegans* Lepeletier, 1806. The type of *Chrysis elegans* is not housed in MNHN (du Buysson 1898). Because the second specimen above matches the sex (*femelle*) and the colour drawing (pl. 6: fig. 20) given by Lepeletier 1806 it could be the holotype that arrived in the Spinola collection together with other types (e.g. *Chrysis comparata*).

Bohart (in Kimsey and Bohart 1991: 407) designated the lectotype of *Chrysis elegans* Lepeletier in MNHN. This designation has to be set aside because it is based on a male (and not female as given in the description), and it was collected in Greece (the typical locality was unknown: "*Je ne sais de quel pays elle est.*"). Hence it cannot be a syntype (or a holotype, to be more accurate). The specimen selected by Bohart is not a syntype and it cannot be considered as a lectotype according to Art. 74.2. It belongs to the *Chrysis elegans* group.

##### Current status.

*Chrysis elegans* Lepeletier, 1806.

#### Chrysis elegantula

Taxon classificationAnimaliaHymenopteraChrysididae

Spinola, 1838

Plate 12

Chrysis elegantula : Spinola 1838: 451.

##### Type locality.

Egypt.

##### Material.

**Holotype** ♀. *Chrysis alternans* Kl. // var. - *Chrysis elegantula*, n. // olim // D. Waltl, Egypt.

**Catalogue Casolari & Casolari Moreno.** *Chrysis alternans*, 132, 23, 95, 1 (box 51).

##### Remarks.

In Casolari and Casolari Moreno (1980: 79) under the name *Chrysis alternans*, the specimen is surely the type of *Chrysis elegantula*. Dahlbom (1854: 236) reported exactly the same names: "*Chrysis alternans* var. *c* ♂ - *Chrysis elegantula Mus. Spinola*". The type is a female and not a male. It belongs to the *comparata*-*scutellaris* group.

##### Current status.

*Chrysis elegantula* Spinola, 1838.

**Plate 12.**
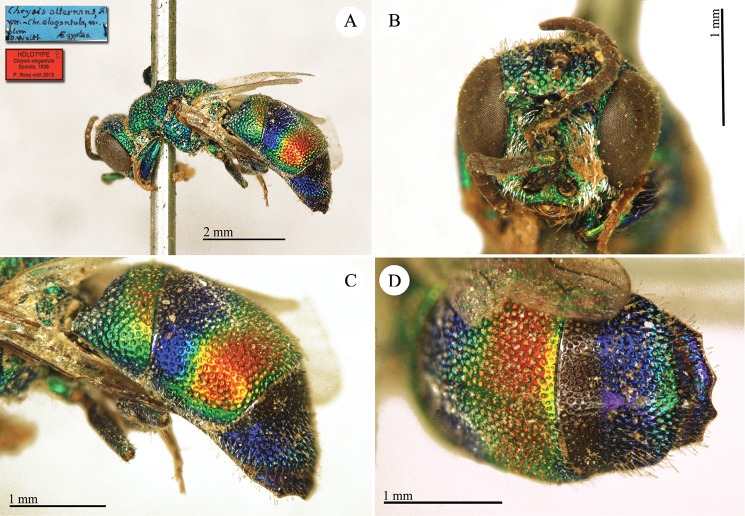
*Chrysis elegantula* Spinola, holotype. **A** Habitus, lateral view **B** head, frontal view **C** metasoma, lateral view **D** second and third metasomal tergites, dorsal view.

#### Chrysis emarginatula

Taxon classificationAnimaliaHymenopteraChrysididae

Spinola, 1808

Plate 13

Chrysis emarginatula : Spinola 1808: 239.

##### Type locality.

"Habitat prope Novas [Novara], rarissima".

##### Material.

**Holotype** ♂. *Chrysis emarginatula* / Spin. Ins. Lig. / Génes [= Genoa].

**Catalogue Casolari & Casolari Moreno.** *Chrysis emarginatula*, 1, 110, 0, 1 (box 50).

##### Remarks.

The type locality in the description does not match the locality written on the label. At Spinola's time Novara was under the dominion of Genoa, and this is the reason for the different localities. There are no known records of *Chrysis emarginatula* around Genoa. It belongs to the *Chrysis elegans* group.

##### Current status.

*Chrysis emarginatula* Spinola, 1808.

**Plate 13.**
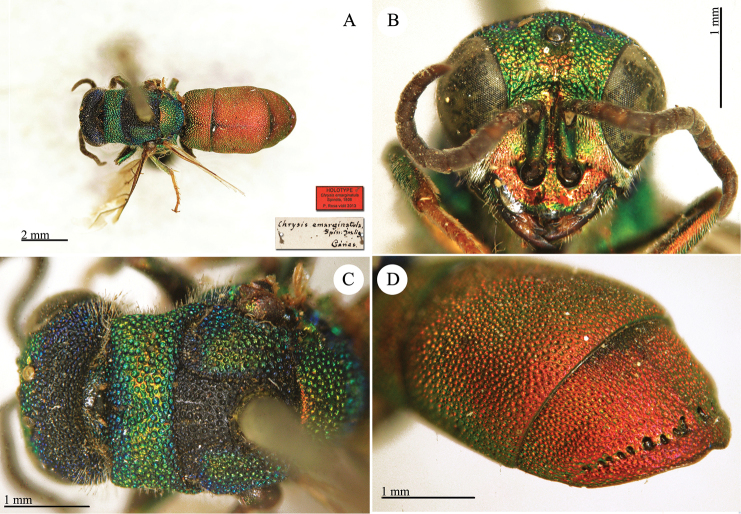
*Chrysis emarginatula* Spinola, holotype. **A** Habitus, dorsal view **B** head, frontal view **C** head and mesosoma, dorsal view **D** second and third metasomal tergites, dorso-lateral view.

#### Chrysis episcopalis

Taxon classificationAnimaliaHymenopteraChrysididae

Spinola, 1838

Chrysis episcopalis : Spinola 1838: 449 *nec* Bloch, 1799.

##### Type locality.

Egypt.

##### Material.

**Lectotype** (here designated) ♀. *Chrysis episcopalis*, Spin.; D. Waltl, Égypte.

**Paralectotype** 1♀. idem.

**Catalogue Casolari & Casolari Moreno.** *Chrysis episcopalis*, 1, 23, 95, 2 (box 51).

##### Remarks.

We designate a lectotype of *Chrysis episcopalis* Spinola using one of two syntypes, the one less severely damaged. The second available syntype is badly damaged, with its metasoma glued on the mesosoma. The name *Chrysis episcopalis* Spinola was used as a valid name until recently, when Kimsey and Bohart (1991: 469) observed that it was a junior homonym of *Chrysis episcopalis* Block, 1799. They suggested to use the name *Chrysis syriaca* Guérin, 1842. However, *Chrysis episcopalis* Block is no longer congeneric with *Chrysis* and no author has used the name *episcopalis* Block after the year 1899. Therefore, according to Article 23.9.5 of the Code, the name *Chrysis episcopalis* Spinola is a valid and available name in the genus *Chrysis*. It belongs to the *Chrysis viridissima* group.

##### Current status.

*Chrysis syriaca* Guérin, 1842 (synonymised by Kimsey and Bohart 1991: 469).

#### Chrysis exsulans

Taxon classificationAnimaliaHymenopteraChrysididae

Dahlbom, 1854

Plate 14

Chrysis exsulans : Dahlbom 1854: 247.

##### Type locality.

"Patria ignota, forte Bengalia; specimen unicum ut. "Chr. fulgidae varietas" amice communicavit D. Spinola".

##### Material.

**Holotype** ♂. *Chrysis exsulans*, Dlbm. // Bengala.

**Catalogue Casolari & Casolari Moreno.** *Chrysis exsulans*, 27, 42, 0, 1 (box 51).

##### Remarks.

Dahlbom realized that this specimen does not have an "exotic" habitus (Bengala was the old name of a district between NW India and Bangladesh) and cited the type locality as "forte Bengalia" [accidentally Bengalia for Bengala]. The species is distributed in North Africa and Near East (Linsenmaier 1999: 218). The type is missing the ventral surface of the metasoma, due to a dermestid attack. It belongs to the *Chrysis ignita* group.

##### Current status.

*Chrysis exsulans* Dahlbom, 1854.

**Plate 14.**
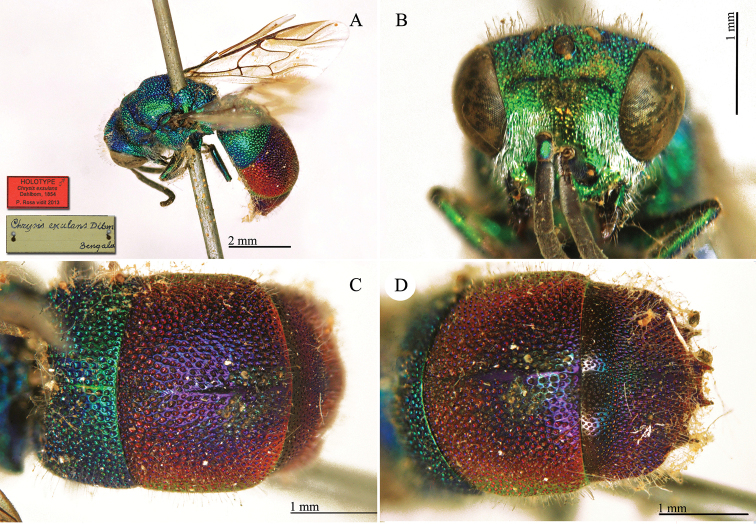
*Chrysis exsulans* Dahlbom, holotype. **A** Habitus, dorso-lateral view **B** head, frontal view **C** first and second metasomal tergites, dorsal view **D** second and third metasomal tergites, dorsal view.

#### Chrysis fasciata

Taxon classificationAnimaliaHymenopteraChrysididae

Spinola, 1840

Chrysis fasciata : Spinola 1840: 202 *nec* Olivier, 1790.

##### Type locality.

Cayenne (French Guyana).

##### Material.

**Holotype** ♂ (?): *Chrysis distinctissima* Dlbm. - *Chrysis fasciata*, m.[ihi] D. Buquet, Cayenna // **Holotype** ♂. *Chrysis intricans* Spinola, R. M. Bohart det. // **Lectotype** ♂. *Chrysis distinctissima* Dahlbom, R.M. Bohart det.

**Catalogue Casolari & Casolari Moreno.** *Chrysis distinctissima*, 27, 56, 1, 2 (box 51).

##### Remarks.

See the remarks under *Chrysis distinctissima* Dahlbom, 1854. It belongs to the *Chrysis intricans* group.

##### Current status.

*Chrysis intricans* Spinola, 1840.

#### Chrysis gayi

Taxon classificationAnimaliaHymenopteraChrysididae

Spinola, 1851

Plate 15

Chrysis gayi : Spinola 1851: 406.

##### Type locality.

Chile.

##### Material.

**Holotype** ♀. *Chrysis Gayi*, Spin. // M. Gay // Chili.

**Catalogue Casolari & Casolari Moreno.** *Chrysis gayi*, 1, 52, 32, 1 (box 51).

##### Remarks.

It belongs to the *Chrysis gibba* group.

##### Current status.

*Chrysis gibba* Brullé, 1846 (synonymised by Mocsáry 1889: 403).

**Plate 15.**
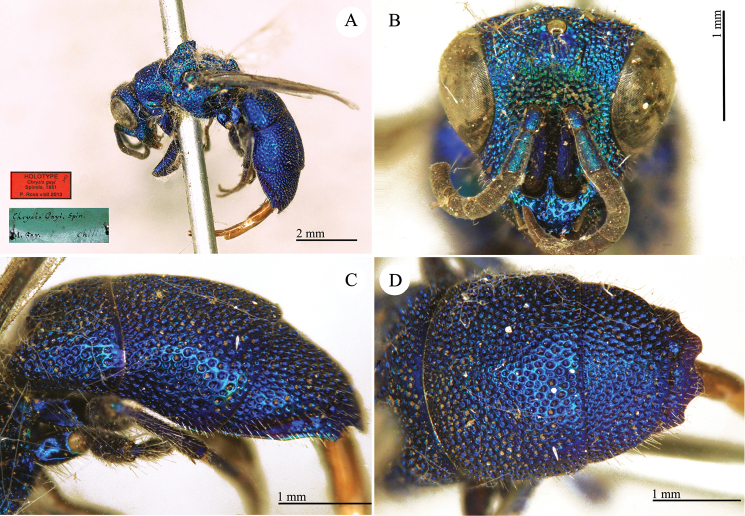
*Chrysis gayi* Spinola, holotype. **A** Habitus, lateral view **B** head, frontal view **C** metasoma, lateral view **D** second and third metasomal tergites, dorsal view.

#### Chrysis grohmanni

Taxon classificationAnimaliaHymenopteraChrysididae

Dahlbom, 1854

Plate 16

Chrysis grohmanni : Dahlbom 1854: 271.

##### Type locality.

"Habitat in Sicilia, a D. Grohmann olim detecta, Mus. D. Spinola".

##### Material.

**Lectotype** (here designated) ♀. *Chrysis grohmanni*, n. / D. Grohmann, Sicilia.

**Catalogue Casolari & Casolari Moreno.** *Chrysis grohmanni*, 1, 204, 34, 3 (box 51).

##### Remarks.

Dahlbom (1854) described *Chrysis grohmanni* based on two specimens: one from the Spinola collection, the other from the Paykull collection (= *Chrysis gloriosa* Dahlbom, 1845). We found three specimens under the name *Chrysis grohmanni* in the Spinola collection, but only one belongs to *Chrysis grohmanni* in its current taxonomic interpretation. The other two specimens are a female of *Chrysis gracillima* Förster, 1853 and a female of *Chrysis bicolor* Lepeletier, 1806, bearing the labels "6288" and "*Chrysis bicolor* Lepel. 127", respectively.

Since there are different species in the series, we designate a lectotype of *Chrysis grohmanni* using the only female matching the original description of the species. The specimen is missing the last seven flagellomeres of the left antenna, and the right wings is glued to the metasoma. It belongs to the *Chrysis succincta* group.

##### Current status.

*Chrysis grohmanni* Dahlbom, 1854.

**Plate 16.**
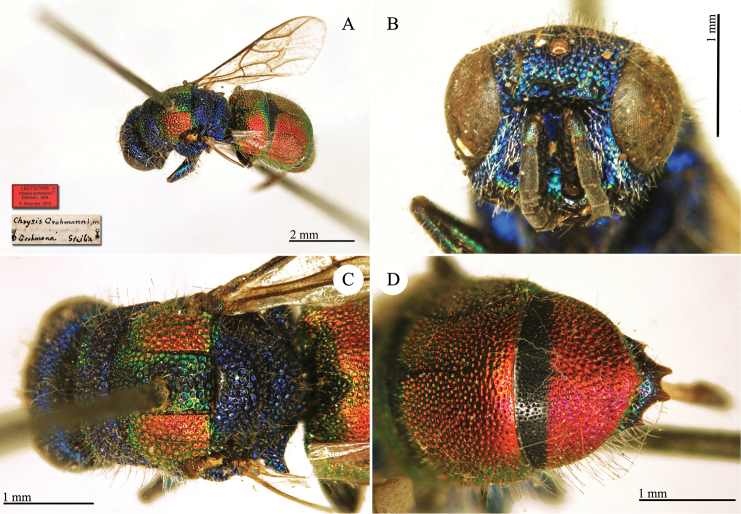
*Chrysis grohmanni* Dahlbom, lectotype. **A** Habitus, dorso-lateral view **B** head, frontal view **C** mesosoma, dorsal view **D** second and third metasomal tergites, dorsal view.

#### Chrysis incrassata

Taxon classificationAnimaliaHymenopteraChrysididae

Spinola, 1838

Plate 17

Chrysis incrassata : Spinola 1838: 454.

##### Type locality.

Corse. "Cette espèce nous a été rapportée de la Corse par M. le docteur Chiesi, de Pise".

##### Material.

**Lectotype** (here designated) ♀. *Chrysis incrassata*, Spin.; D. Chiesi, Corse.

**Paralectotype** 1 ♀. idem.

**Catalogue Casolari & Casolari Moreno.** *Chrysis incrassata*, 1, 51, 12, 2 (box 50).

##### Remarks.

Spinola (1838) did not write how many specimens the type series consisted of. In the Spinola collection, there are two specimens prepared in the same way under one locality written on the main label. These two specimens likely represent syntypes. One of the syntypes is badly damaged by a dermestid attack. We designate a lectotype of *Chrysis incrassata* Spinola using the less damaged of the above two specimens. The lectotype lacks the last flagellomeres of the right antenna and the last tarsi of the right hind leg. The type locality is Corsica and not Egypt, even though *Chrysis incrassata* was described by Spinola in the paper on Egyptian Hymenoptera.

##### Current status.

*Pseudospinolia incrassata* (Spinola, 1838) (transferred by Kimsey and Bohart 1991: 547).

**Plate 17.**
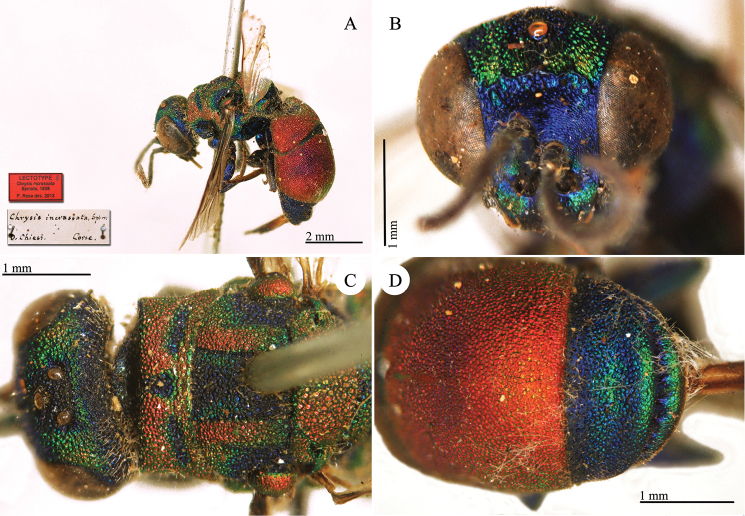
*Chrysis incrassata* Spinola, lectotype. **A** Habitus, lateral view **B** head, frontal view **C** head and mesosoma, dorsal view **D** second and third metasomal tergites, dorsal view.

#### Chrysis intricans

Taxon classificationAnimaliaHymenopteraChrysididae

Spinola, 1840

Chrysis intricans : Spinola 1840: 203.

##### Type locality.

unkown.

##### Material.

**Holotype** ♂. *Chrysis distinctissima* / Dlbm. - *Chrysis fasciata*, m. / D. Buquet, Cayenna // **Holotype** ♂. *Chrysis intricans* Spinola, R. M. Bohart det. // **Lectotype** ♂. *Chrysis distinctissima* Dahlbom, R.M. Bohart det.

**Catalogue Casolari & Casolari Moreno.** *Chrysis distinctissima*, 27, 56, 1, 2 (box 51).

##### Remarks.

See the remarks under the name *Chrysis distinctissima* Dahlbom, 1854. It belongs to the *Chrysis intricans* group.

##### Current status.

*Chrysis intricans* Spinola, 1840.

#### Chrysis laeta

Taxon classificationAnimaliaHymenopteraChrysididae

Dahlbom, 1854

Plate 18

Chrysis laeta : Dahlbom 1854: 223.

##### Type locality.

"Habitat in promontorio bonae spei, D. Draege, Mus. DD. Drewsen et Spinola; in Guinea, D. Westermann".

##### Material.

**Paralectotypes** 1 ♂ and 3♀♀. *Chrysis laeta*, Dr.; D. Draege, Cap. B. Esp.

**Catalogue Casolari & Casolari Moreno.** *Chrysis laeta*, 45, 53, 21, 4 (box 51).

##### Remarks.

Bohart (in Kimsey and Bohart 1991: 430) designated a lectotype using a specimen housed in ZMUC. The interpretation of the species given by Linsenmaier (1999: 177) is not correct, since it belongs to the *Chrysis splendidula-senegalensis* group and not to the *comparata*-*scutellaris* group.

##### Current status.

*Chrysis laeta* Dahlbom, 1854.

**Plate 18.**
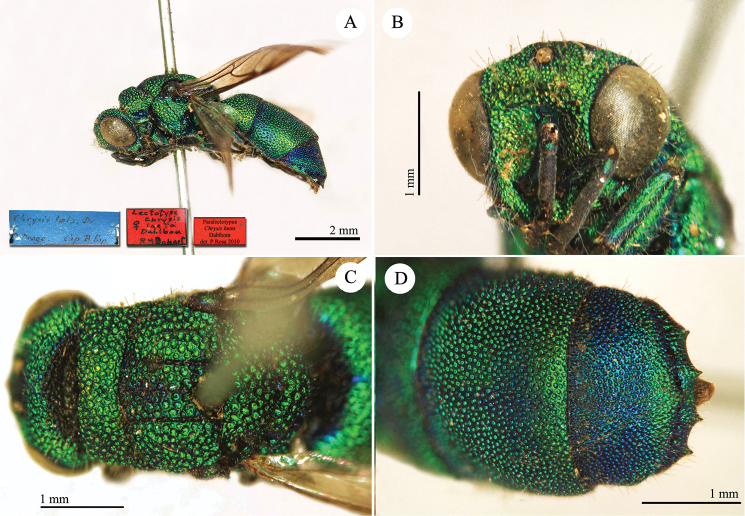
*Chrysis laeta* Dahlbom, paralectotype. **A** Habitus, lateral view **B** head, frontal view **C** mesosoma, dorsal view **D** second and third metasomal tergites, dorsal view.

#### Chrysis malachitica

Taxon classificationAnimaliaHymenopteraChrysididae

Dahlbom, 1854

Plate 19

Chrysis malachitica : Dahlbom 1854: 335.

##### Type locality.

"Habitat in Africa meridionali, ad promontorium bonae spei a Dom. Draege detecta. Specimina tantum 2 vidi, unum a Dom. Spinola et alterum a Dom. Drewsen communicata".

##### Material.

**Paralectotype** 1 ♀. *Chrysis malachitica*, Dr..; inédite; D. Drage, Cap. B. Esp.

**Catalogue Casolari & Casolari Moreno.** *Chrysis malachitica*, 45, 53, 21, 1 (box 51).

##### Remarks.

Bohart (in Kimsey and Bohart 1991: 435) designated a lectotype at ZMUC. It belongs to the *Chrysis smaragdula* group.

##### Current status.

*Chrysis malachitica* Dahlbom, 1854.

**Plate 19.**
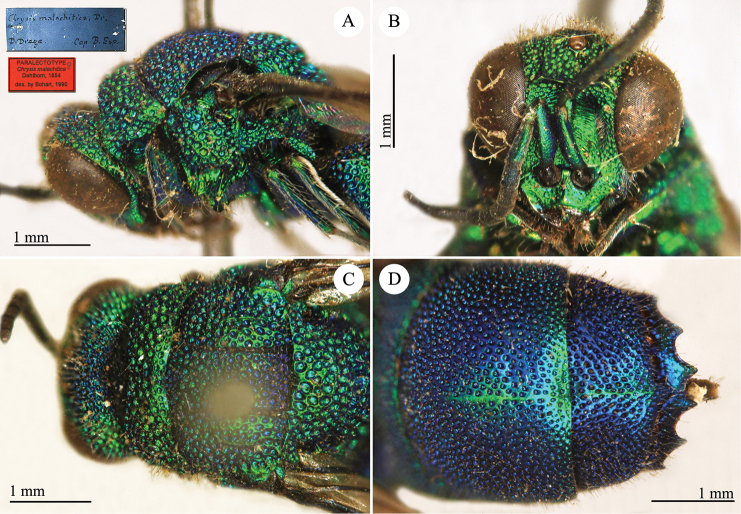
*Chrysis malachitica* Dahlbom, paralectotype. **A** Head and mesosoma, lateral view **B** head, frontal view **C** head and mesosoma, lateral view **D** second and third metasomal tergites, dorsal view.

#### Chrysis megerlei

Taxon classificationAnimaliaHymenopteraChrysididae

Dahlbom, 1854

Plate 20

Chrysis megerlei : Dahlbom 1854: 297.

##### Type locality.

"Habitat ad Veronam, a D. Conti detecta; Mus. Spinolae".

##### Material.

**Holotype** ♂. *Chrysis megerlei* Dlbm. - *Chrysis analis*, Meg.[erle] D. Conti, Verona.

**Catalogue Casolari & Casolari Moreno.** *Chrysis megerlei*, 27, 229, 14, 2 (box 51).

##### Current status.

*Praestochrysis megerlei* (Dahlbom, 1854) (transferred by Kimsey and Bohart 1991: 533).

**Plate 20.**
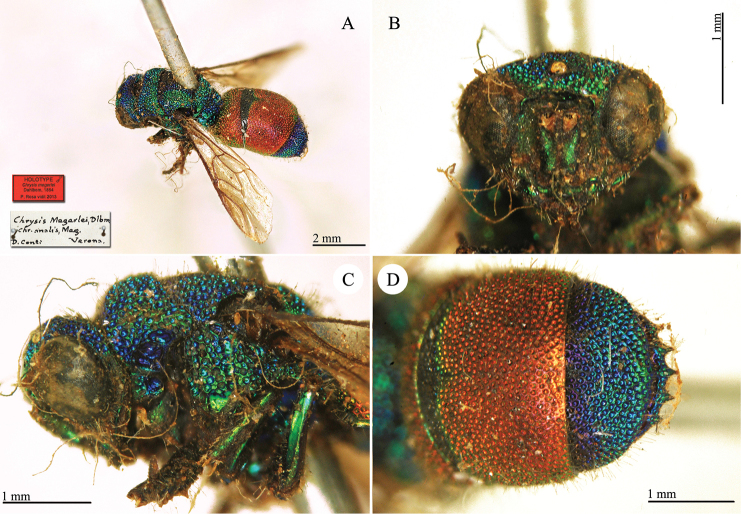
*Chrysis megerlei* Dahlbom, holotype. **A** Habitus, dorso-lateral view **B** head, frontal view **C** head and mesosoma, lateral view **D** second and third metasomal tergites, dorsal view.

#### Chrysis modica

Taxon classificationAnimaliaHymenopteraChrysididae

Dahlbom, 1854

Chrysis modica : Dahlbom 1854: 326 [*nec* 1850: 140].

##### Type locality.

"Habitat in Africa; 3 specimina vidimus; unum in Guinea lectum, a D. Westermann nobis olim donatum; alterum e promontorio bonae spei a D. Spinola ut variet. Chrysidis malachiticae communicatura; tertium e Port Natal a D. J. Wahlberg reportatum, Mus. R. Acad. Scient. Stockholm".

##### Material.

**Paralectotype** 1 ♀. *Chrysis modica*, Dlbm. D. Dreage, Pr. B. Esp.

**Catalogue Casolari & Casolari Moreno.** *Chrysis modica*, 27, 53, 21, 1 (box 51).

##### Remarks.

Dahlbom (1854) described *Chrysis modica* based on three specimens collected in Guinea (ZMUC), Cape of Good Hope (Spinola’s collection) and Port Natal (NHRS). Bohart (in Kimsey and Bohart 1991: 437) selected the latter as lectotype. It belongs to the *Chrysis smaragdula* group.

##### Current status.

*Chrysis mediocris* Dahlbom, 1845 (synonymised by Kimsey and Bohart 1991: 437).

#### Chrysis mucronata

Taxon classificationAnimaliaHymenopteraChrysididae

Dahlbom, 1854

Plate 21

Chrysis mucronata : Dahlbom 1854: 344 *nec* Brullé, 1846.

##### Type locality.

"Habitat in Africa meridionali, ad promontorium bonae spei a Dom. Draege detecta; unicum specimen nobis communicavit Dom. Spinola".

##### Material.

**Holotype** ♀. *Chrysis mucronata* Dlbm – (*Pyria*) Encycl. Sud Africa.

**Catalogue Casolari & Casolari Moreno.** *Chrysis mucronata*, 27, 211, 0, 1 (box 51).

##### Remarks.

It is considered as a synonym of *Chrysis laborans* Costa, 1865. The type of *Chrysis laborans* should be housed in Napoli (Museum of the Ferdinando II University), but it is lost. The last author who examined this type was du Buysson (1905). It belongs to the *Chrysis wahlbergi* group.

##### Current status.

*Chrysis laborans* Costa, 1865 (synonymised by Kimsey and Bohart 1991: 429).

**Plate 21.**
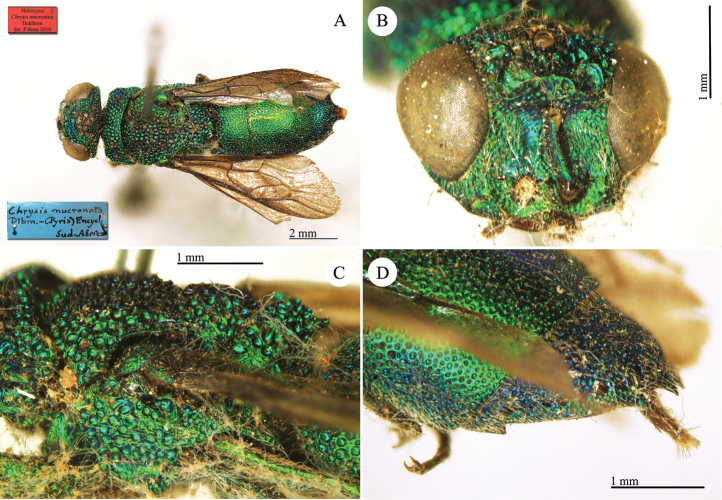
*Chrysis mucronata* Dahlbom, holotype **A** Habitus, dorsal view **B** head, frontal view **C** mesosoma, lateral view **D** second and third metasomal tergites, lateral view.

#### Chrysis pallidicornis

Taxon classificationAnimaliaHymenopteraChrysididae

Spinola, 1838

Plate 22

Chrysis pallidicornis : Spinola 1838: 451.

##### Type locality.

Egypt.

##### Material.

**Lectotype** (here designated) ♀. *Chrysis pallidicornis* m. N. sp., Egitto / 204.

**Paralectotype** 1 ♂, no labels.

**Catalogue Casolari & Casolari Moreno.** *Chrysis pallidicornis*, 1, 23, 0, 2 (box 51).

##### Remarks.

**Plate 22.**
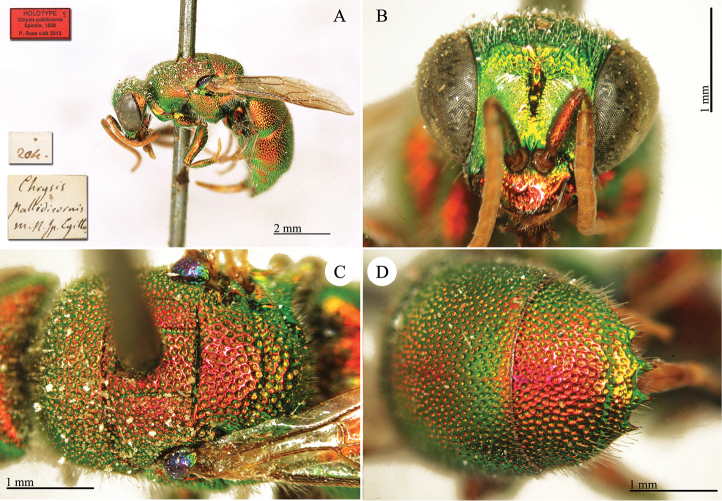
*Chrysis pallidicornis* Spinola, lectotype **A** Habitus, lateral view **B** head, frontal view **C** mesosoma, dorsal view **D** second and third metasomal tergites, dorsal view.

There are two specimens under this name in the Spinola collection: one male and one female. However, they do not exhibit the typical large label at the base of the specimen series. Only the female bears two labels: [204] and [*Chrysis pallidicornis* m. [mihi] n. sp., Egitto] handwritten by Spinola. Linsenmaier (1959) examined both types. Since it is not mentioned in the original description how many specimens the author examined, Linsenmaier considered both as syntypes. We designate the specimen bearing Spinola’s handwritten labels as lectotype to ensure a correct future identification in this species, which belong to a species-rich and taxonomically currently complicated species group (Zimmermann 1959). The selected specimen matches Linsenmaier’s interpretation of the species (Linsenmaier 1959). On Plate 22, the type label is erroneously given as holotype instead of lectotype. It belongs to the *Chrysis pallidicornis* group.

##### Current status.

*Chrysis pallidicornis* Spinola, 1838.

#### Chrysis palliditarsis

Taxon classificationAnimaliaHymenopteraChrysididae

Spinola, 1838

Plate 23

Chrysis palliditarsis : Spinola 1838: 449.

##### Type locality.

Egypt.

##### Material.

**Holotype** ♂. *Chrysis palliditarsis*, Spin.; … *fasciolata*, Klug ?; D. Waltl, Égypte.

**Catalogue Casolari & Casolari Moreno.** *Chrysis palliditarsis*, 1, 23, 95, 1 (box 51).

##### Remarks.

The type is badly damaged, lacking the metasoma. It belongs to the *Chrysis comparata*-*scutellaris* group.

##### Current status.

*Chrysis palliditarsis* Spinola, 1838.

**Plate 23.**
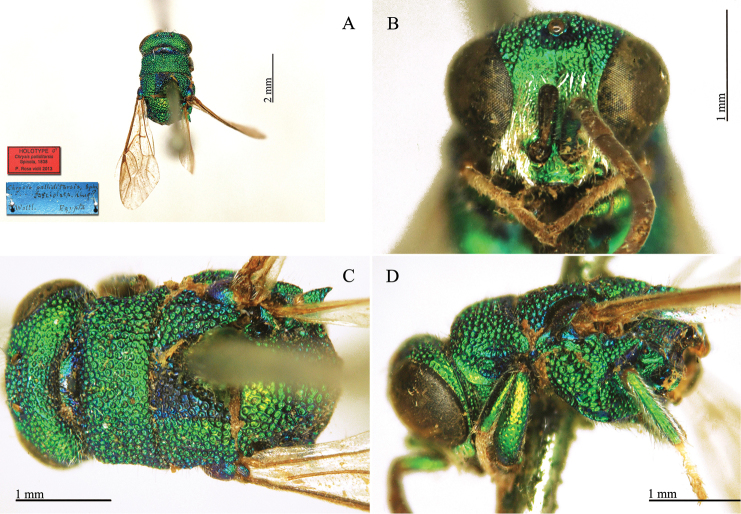
*Chrysis palliditarsis* Spinola, holotype **A** Head and mesosoma, dorsal view **B** head, frontal view **C** head and mesosoma, dorsal view **D** head and mesosoma, lateral view.

#### Chrysis pulchella

Taxon classificationAnimaliaHymenopteraChrysididae

Spinola, 1808

Plate 24

Chrysis pulchella : Spinola 1808: 28.

##### Type locality.

"*Habitat in agro Arquatensi* [Arquata Scrivia], *rara*".

##### Material.

**Lectotype** (here designated) ♂. *Chrysis pulchella* Lepell. Liguria.

**Catalogue Casolari & Casolari Moreno.** *Chrysis pulchella*, 148, 145, 0, 3 (box 51).

##### Remarks.

The type series was based on multiple specimens. When Spinola wrote "*rara*" it meant that he examined few specimens; when he wrote "*rarissima*" it meant that he examined only one specimen. At present there are three specimens in the Spinola collection under the name *Chrysis pulchella*: one male, without a metasoma, acquired via the Serville collection; one male of *Chrysis bicolor* Lepeletier, received by a French entomologist and bearing a rounded numerical label (6298); and a male specimen that is referable to the original description of the species and designated here as lectotype. It is unfortunately badly damaged, missing part of its head, all sternites, the internal urites, and some of its legs. It belongs to the *Chrysis pulchella* group.

##### Current status.

*Chrysis pulchella* Spinola, 1808.

**Plate 24.**
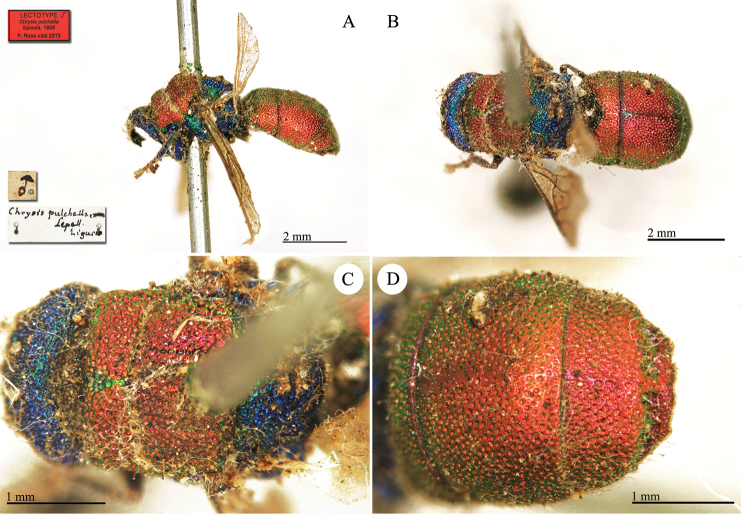
*Chrysis pulchella* Spinola, lectotype **A** Habitus, lateral view **B** habitus, dorsal view **C** head and mesosoma, dorsal view **D** second and third metasomal tergites, dorsal view.

#### Chrysis punctatissima

Taxon classificationAnimaliaHymenopteraChrysididae

Spinola, 1840

Plate 25

Chrysis punctatissima : Spinola 1840: 200 *nec* Villers, 1789.

##### Type locality.

Cayenne, French Guyana.

##### Material.

**Syntypes** 1♂ and 2♀♀. *Chrysis fasciata* Fab. – *Chrysis punctatissima* m.[ihi], olim D. Buquet, Cayenna / *Chrysis carina* ♂ R. M. Bohart det.

**Catalogue Casolari & Casolari Moreno.** *Chrysis punctatissima*, 59, 56, 1, 3 (box 51).

##### Remarks.

Dahlbom (1854: 197) treated this species as a synonym of *Chrysis fasciata* Fabricius; for likely this reason, we find written on Spinola’s label: "*Chrysis fasciata* Fab - *Chrysis punctatissima* mihi".

##### Current status.

*Neochrysis carina* (Brullé, 1846) (synonymised by Mocsáry 1889: 339; *Neochrysis carina* is the first available name; transferred by Kimsey 1985: 276).

**Plate 25.**
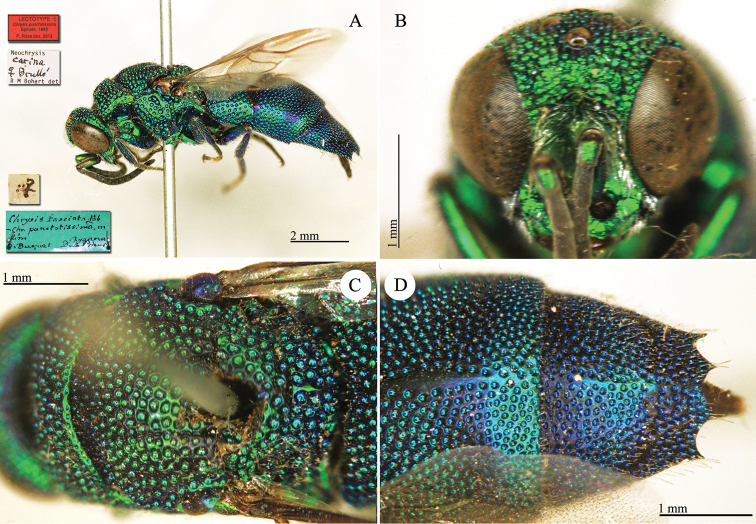
*Chrysis punctatissima* Spinola, lectotype **A** Habitus, lateral view **B** head, frontal view **C** mesosoma, dorsal view **D** second and third metasomal tergites, dorsal view.

#### Chrysis purpurata

Taxon classificationAnimaliaHymenopteraChrysididae

Fabricius, 1787

Plate 26A

Chrysis purpurata : Fabricius 1787. ICZN Opinion 1906: 195.

##### Material.

**Neotype** ♀. *Euchroeus purpureus* Latr.. – *purpuratus*, (*Chrysis*) Fab. Coll. Latr., Fr. mérid. // *Chrysis purpurata* F. 1787 **Neotypus** M. Pavesi & F. Strumia det. 1998.

**Catalogue Casolari & Casolari Moreno.** *Euchraeus purpureus*, 149, 99, 51, 4 (box 52).

##### Remarks.

Kimsey (1988: 272) designated the lectotype of *Chrysis purpurata* at ZMUC, based on one specimen labelled *Chrysis purpurata* but belonging to another species: *Chrysis iris* Christ, 1791. The consequence was that the generic name *Euchroeus* Latreille, 1809 became a junior synonym of *Chrysis* Linnaeus, 1761 (Pavesi and Strumia 1997) and the generic name *Euchroeus* was replaced by the first available name: *Brugmoia* Radoszkowski, 1877. The species formerly known as *Euchroeus purpuratus* auctorum was replaced by the name *Brugmoia quadrata* (Shuckard, 1837). The Commission on the ICZN approved the documentations provided by Pavesi and Strumia (1997) as valid and suppressed the lectotype designated by Kimsey and conserved the generic name *Euchroeus* Latreille and the specific name *purpurata* Fabricius. Pavesi and Strumia (ICZN 1998) designated the neotype of *Chrysis purpurata* in the Spinola collection.

##### Current status.

*Euchroeus purpuratus* (Fabricius, 1787) (transferred by Latreille 1809).

**Plate 26.**
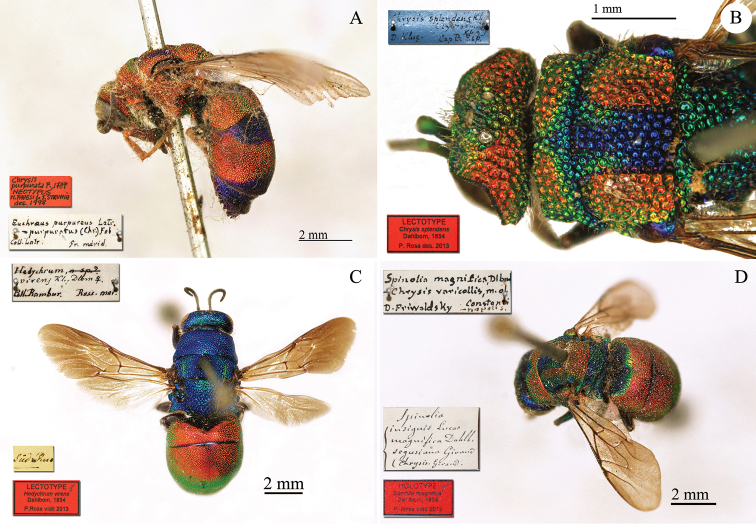
**A** *Chrysis purpurata* Fabricius, neotype, habitus, lateral view **B** *Chrysis splendens* Dahlbom, lectotype, head and mesosoma, dorsal view **C** *Hedychrum virens* Dahlbom, lectotype, habitus, dorsal view **D** *Spinolia magnifica* Dahlbom, holotype, habitus, dorsal view.

#### Chrysis ramburi

Taxon classificationAnimaliaHymenopteraChrysididae

Dahlbom, 1854

Plate 27

Chrysis ramburi : Dahlbom 1854: 249.

##### Type locality.

"Habitat in Europa meridionali rarius; marem unicum in Hispania a D. Rambur detectum et feminam unicam in Lombardia a D. Christophori lectam communicavit D. Spinola".

##### Material.

**Lectotype** (here designated) ♂. *Chrysis Ramburi*, Spin. n. sp.?. D. Rambur, Espagne, Sierra Nevada.

**Paralectotype** ♀. *Chrysis Ramburi*, Spin. n. sp.?. D. Rambur, Espagne, Sierra Nevada.

**Catalogue Casolari & Casolari Moreno.** *Chrysis ramburi*, 1, 101, 74, 2 (box 51).

##### Remarks.

The type series includes two specimens: one male from Spain and one female from Lombardy (Italy). Both syntypes are housed in the Spinola collection. They match the original description, but they belong to two different species. The male belongs to *Chrysis ramburi* Dahlbom, whereas the female to *Chrysis chrysostigma* Mocsáry, 1889. Dahlbom (1854) described them as male and female of the same species based on the colour of the last visible tergite. The differences given in the text between the two taxa were supposed to be sexual dimorphic characteristics.

*Chrysis ramburi* in Europe is present only on the Iberian Peninsula and occasionally in south France, whereas it is more frequent in North Africa, particularly in Morocco. *Chrysis chrysostigma*, on the other hand, is widely distributed in southern Europe from France to the Czech Republic, and it is quite common in Italy. The two species were already considered separate taxa by various authors (e.g. Linsenmaier 1987: 151; Tyrner 2007: 48). Kimsey and Bohart (1991: 455) placed *Chrysis chrysostigma* in synonymy of *Chrysis ramburi*. Móczár (1965: 172) designated a lectotype of *Chrysis chrysostigma* at HNHM. We therefore designate a lectotype of *Chrysis ramburi* Dahlbom to fix the current interpretation of the species and to avoid future misidentifications based on the fact that Dahlbom described two different species under one and the same name. The selected lectotype is based on the male specimen with a thin layer of mould. It belongs to the *Chrysis comparata*-*scutellaris* group.

##### Current status.

*Chrysis ramburi* Dahlbom, 1854.

**Plate 27.**
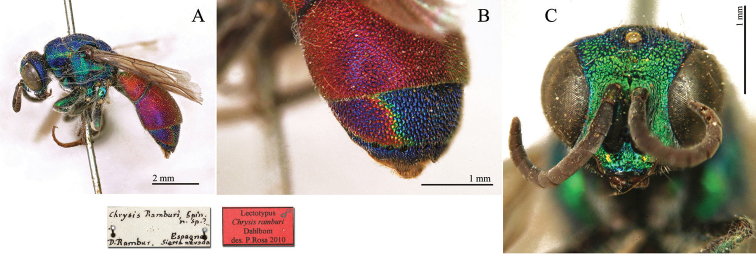
*Chrysis ramburi* Dahlbom, lectotype **A** Habitus, lateral view **B** second and third metasomal tergites, dorso-lateral view **C** head, frontal view.

#### Chrysis refulgens

Taxon classificationAnimaliaHymenopteraChrysididae

Spinola, 1806

Plate 28

Chrysis refulgens : Spinola 1806: 8.

##### Type locality.

"Habitat prope Genuam [Genoa], haud infrequens".

##### Material.

**Lectotype** (here designated) ♀. *Chrysis refulgens*, Spin. 36. *scutellata*, Panz. Genes [= Genoa].

**Catalogue Casolari & Casolari Moreno.** *Chrysis refulgens*, 1, 110, 0, 2 (box 50).

##### Remarks.

Spinola (1806) described *Chrysis refulgens* based on a number of specimens (*haud infrequens*) collected in Liguria (Genoa). In his collection, there are two specimens collected at Genoa, but only the single one female specimen of these belongs to this species; the male specimen belongs to *Chrysis graelsii* Guérin, 1846, which therefore has to be excluded from the syntype series. To avoid future misinterpretation of the specimens in the Spinola collection, we designate the above female matching the description provided by Spinola as the lectotype of *Chrysis refulgens* Spinola. It is missing the right antenna and the left flagellum. Kimsey and Bohart (1991: 495) cited another type at MNHN. It belongs to the *Chrysis radians* group.

##### Current status.

*Chrysura refulgens* (Spinola, 1806) (transferred by Kimsey and Bohart 1991: 495).

**Plate 28.**
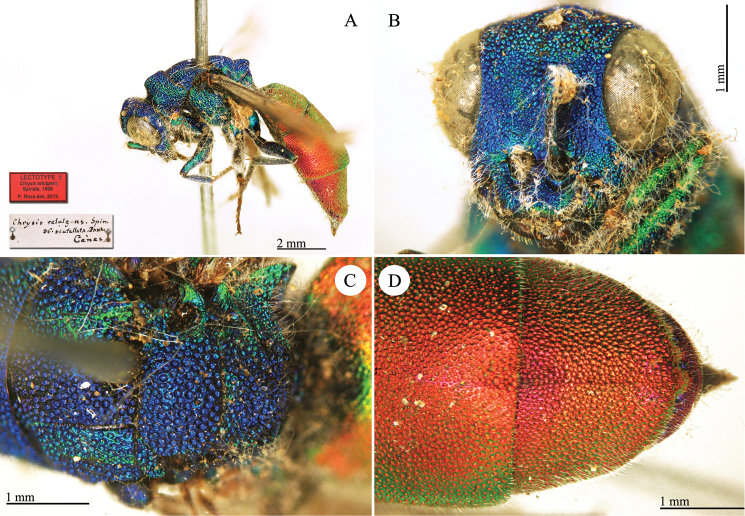
*Chrysis refulgens* Spinola, lectotype **A** Habitus, lateral view **B** head, frontal view **C** mesosoma, dorsal view **D** second and third metasomal tergites, dorsal view.

#### Chrysis reichei

Taxon classificationAnimaliaHymenopteraChrysididae

Dahlbom, 1854

Plate 29

Chrysis reichei : Dahlbom 1854: 218.

##### Type locality.

"Habitat in Africa meridionali ad Caput bonae spei, Mus. D. Spinolae".

##### Material.

**Holotype** ♀. *Chrysis reichei*, Spin. D. Reiche Coromandal.

**Catalogue Casolari & Casolari Moreno.** *Chrysis reichei*, 1, 55, 75, 1 (box 51).

##### Remarks.

Spinola (1838: 448) described *Pyria reichei* (currently *Chrysis lincea* Fabricius, 1775) based on one specimen collected by Reiche at Cape of Good Hope, in South Africa. Dahlbom (1854: 218) described a new species based on another specimen received from Spinola under the name *Chrysis reichei* and collected at Coromandel in the province of Minas Gerais in Brazil. This species is clearly different from *Pyria reichei* Spinola. Dahlbom (1854) redescribed the species because the specimen received earlier did not match the original description. However, he actually described a new species, confused the type locality Coromandel, Brazil, with Coromandal, which is in South Africa, Mpumalanga State. The holotype of *Pyria reichei* Spinola is currently considered lost. It belongs to the *ignita* group.

##### Current status.

*Chrysis brasiliensis* Brullé, 1846 (synonymised by Kimsey and Bohart 1991: 391).

**Plate 29.**
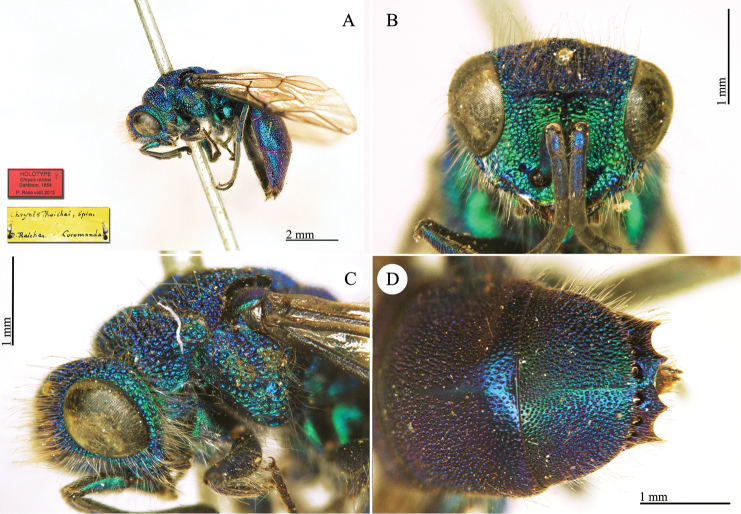
*Chrysis reichei* Dahlbom, holotype **A** Habitus, lateral view **B** head, frontal view **C** head and mesosoma, lateral view **D** second and third metasomal tergites, dorsal view.

#### Chrysis rutilans

Taxon classificationAnimaliaHymenopteraChrysididae

Dahlbom, 1854

Chrysis rutilans Dahlbom: 1854: 260 *nec* Olivier, 1790.

##### Type locality.

"Habitat in Lusitania, Italia, Gallia, Turcia ad Constantinopol. Feminam, in Tirolia a CI. Prof. Apex, detectam, in Museo Berolinensi anno 1847 vidi.".

##### Material.

**Syntype** 1 ♂. *Chrysis rutilans*, Oliv. Coll. Latr., Fr. mérid.

**Catalogue Casolari & Casolari Moreno.** *Chrysis rutilans*, 199, 99, 51, 1 (box 51).

**Syntype** (sex unknwon; 1 ♂ according the description): *Chrysis bicolor*, Dlbm D. Friwaldsky, Graecia.

**Catalogue Casolari & Casolari Moreno.** *Chrysis bicolor*, 27, 109, 30, 1 (box 50).

##### Remarks.

In the Spinola collection there are two specimens listed by Dahlbom (1854) under his diagnosis of *Chrysis rutilans*. Dahlbom (1854) gave a description of *Chrysis rutilans* Olivier, assuming that at least one of Spinola’s specimens was a type or was compared by Latreille (Dahlbom 1854) with a type: "*Chrysis rutilans Olivier, teste Latreille olim in litteris ad Spinola*" and "*Chrysis Friwaldskyi Spinola*" both specimens housed in "Mus. Spinolae". The same species had been described one year before by Förster (1953) as *Chrysis chrysoprasina*. It belongs to the *Chrysis comparata*-*scutellaris* group.

##### Current status.

*Chrysis chrysoprasina* Förster, 1853 (synonymised by Mocsáry 1887: 16).

#### Chrysis sicula

Taxon classificationAnimaliaHymenopteraChrysididae

Dahlbom, 1854

Chrysis sicula : Dahlbom 1854: 158 (given as var. *ab* and var. *f*).

##### Type locality.

Sicily.

##### Material.

**Holotype** (?) ♀. *Chrysis elegans*, Lepell. et Dlbm. var. *a*, ♀ - *β*. id. var. *ab*. Dlbm. - *Chrysis sicula*, mihi olim D. Grohmann. Sicilia.

**Catalogue Casolari & Casolari Moreno.** *Chrysis elegans*, 164, 204, 34, 2 (box 50).

##### Remarks.

Dahlbom (1854) refered to three specimens housed in the Spinola collection named *Chrysis sicula*. The male specimen is no longer present in the collection, and it was not listed by Spinola himself, who listed only two female specimens: *Chrysis elegans* var. *a* and "*β*" var. *ab*. However, there is no perfect match between Dahlbom (1854) descriptions and Spinola’s label, since the form "*β*" referred to a male (described with green mesosoma), whereas in the Spinola collection the label "*β*" refers to a female. We interpret *Chrysis sicula* as a **new synonym** of *Chrysis elegans* Lepeletier, 1806. It belongs to the *Chrysis elegans* group.

##### Current status.

*Chrysis elegans* Lepeletier Lepeletier, 1806.

#### Chrysis singularis

Taxon classificationAnimaliaHymenopteraChrysididae

Spinola, 1838

Plate 30

Chrysis singularis : Spinola 1838: 452.

##### Type locality.

Egypt.

##### Material.

**Holotype** ♀. *Spintharis singularis* (*Chrysis*) Spin. D. Waltl, Égypte.

**Catalogue Casolari & Casolari Moreno.** *Spintharis singularis*, 1, 23, 91, 1 (box 52).

##### Current status.

*Euchroeus singularis* (Spinola, 1838) (transferred by Linsenmaier 1959: 71).

**Plate 30.**
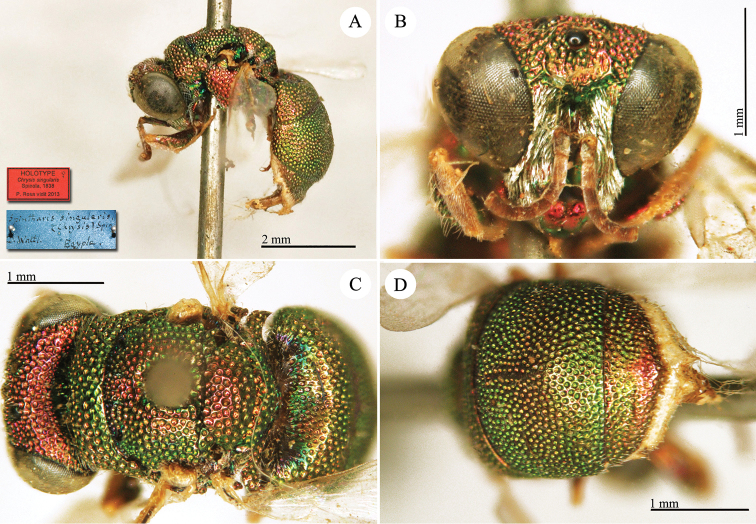
*Chrysis singularis* Spinola, holotype **A** Habitus, lateral view **B** head, frontal view **C** mesosoma, dorsal view **D** second and third metasomal tergites, dorsal view.

#### Chrysis smaragdula

Taxon classificationAnimaliaHymenopteraChrysididae

Lepeletier & Serville, 1825

Plate 31

Chrysis smaragdula : Lepeletier and Serville 1825: 494 *nec* Fabricius, 1775.

##### Type locality.

French Guyana (introduced or locality in error).

##### Material.

**Holotype** ♀. *Chrysis smaragdula*, Fab. Lepel.; Affrica, America, 125 // 6278.

**Catalogue Casolari & Casolari Moreno.** *Chrysis smaradgula*, 148, 0, 0, 1 (box 52).

##### Remarks.

It belongs to the *Chrysis smaradgula* group.

##### Current status.

*Chrysis stilboides* Spinola, 1838 (synonymised by Kimsey and Bohart 1991: 466).

**Plate 31.**
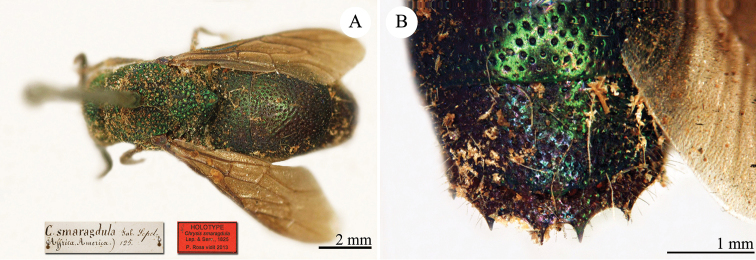
*Chrysis smaragdula* Lepeletier & Serville, holotype **A** Habitus, dorsal view **B** second and third metasomal tergites, dorsal view.

#### Chrysis spinigera

Taxon classificationAnimaliaHymenopteraChrysididae

Spinola, 1840

Plate 32

Chrysis spinigera : Spinola 1840: 201.

##### Type locality.

Cayenne (French Guyana).

##### Material.

**Holotype** ♀. *Chrysis spinigera*, Spin. D. Buquet, Cayenne.

**Catalogue Casolari & Casolari Moreno.** *Chrysis spinigera*, 1, 56, 1, 1 (box 51).

##### Current status.

*Exochrysis spinigera* (Spinola, 1840) (transferred by Kimsey 1985: 271).

**Plate 32.**
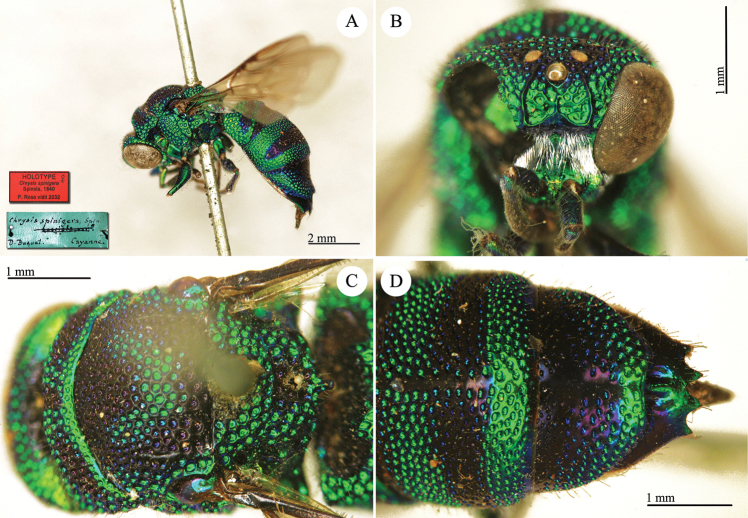
*Chrysis spinigera* Spinola, holotype **A** Habitus, lateral view **B** head, frontal view **C** mesosoma, dorsal view **D** second and third metasomal tergites, dorsal view.

#### Chrysis splendens

Taxon classificationAnimaliaHymenopteraChrysididae

Dahlbom, 1854

Plate 26B

Chrysis splendens : Dahlbom 1854: 312.

##### Type locality.

"Habitat in Africa meridionali ad promontorium bonae spei, Mus. D. Spinola".

##### Material.

**Lectotype** (here designated) ♂. *Chrysis splendens*, Kl. (*Pyrosomus*) Klug D. Klug, Cap. B. Esp.

**Catalogue Casolari & Casolari Moreno.** *Chrysis splendens*, 132, 53, 49, 1 (box 51).

##### Remarks.

Dahlbom (1854) described *Chrysis splendens* based on at least two specimens found in the Spinola collection and listed as var. *a* and var. *b*. Only one specimen seems to be preserved. This specimen is seriously damaged, missing its metasoma (except for the first tergite), its compound eyes, the flagellomeres of the right antenna, and the right foreleg. Therefore, it is impossibile to assign this specimen to one of the two variations described by Dahlbom. However, the remaining part of the head and the mesosoma are species-diagnostic of this beautiful African species. Bohart (in Kimsey and Bohart 1991: 465) designated the male as lectotype in the Spinola collection, but the only specimen left in the collection is without any label. We do not consider this a valid lectotype designation and set it aside. We here designate the above specimen as lectotype of *Chrysis splendens*, because it is housed in the author’s collection (Recommendation 74D of the Code) and Bohart (1991) already listed it in this collection. It belongs to the *Chrysis splendens* group.

##### Current status.

*Chrysis splendens* Dahlbom, 1854.

#### Chrysis succinctula

Taxon classificationAnimaliaHymenopteraChrysididae

Dahlbom, 1854

Plate 33

Chrysis succinctula : Dahlbom 1854: 179.

##### Type locality.

"Habitat in Europa media et meridionali ad Berolinum, D. Klug ad Genuam D. Spinola; ad Milanum, D. Christophori."

##### Material.

**Lectotype** (here designated) ♀. *Chrysis succinctula*, Sp. n. sp. cum var. - *Chrysis assimilis*, De Crist. Genes [Genova] et Milan.

**Paralectotype** 1 ♀. idem.

**Catalogue Casolari & Casolari Moreno.** *Chrysis succinctula*, 1, 110/160, 0, 3 (box 51).

##### Remarks.

The description of *Chrysis succinctula* is based on at least three specimens: two Italian specimens collected at Genoa and Milan (listed under the unpublished names *Chrysis succinctula* and *Chrysis assimilis* De Cristofori) housed in the Spinola collection and the third one from the MNHU (Klug), without locality. We were not able to find the specimen in MNHU. It was presumably destroyed or merged with samples of other species and now cannot be identified as syntype any more. In the Spinola collection, there are two syntypes and a third specimen collected in Corsica and belonging to the species *Chrysis pseudogribodoi* Linsenmaier, most likely added in the column after the death of Spinola. In any case, it does not belong to the type series. The two syntypes do not match the current interpretation of the species given by Linsenmaier (1959: 115), since they belong to the species *Chrysis germari* Wesmael, 1839. The description and the drawings given by Dahlbom agree with this new synonymy. Dahlbom described *succinctula* in his third "*Phalanx*" (*Chrysides ano unidentate*) and placed in this group only two species: *Chrysis succinctula* and *Chrysis leachii* Shuchard, 1837. Both *Chrysis germari* and *Chrysis leachii* have the last tergite with a visible tooth or emargination. Even Dahlbom’s description of *Chrysis succinctula* does not exclude synonymy with *Chrysis germari*: "*Pronotum 1. cyaneum 1. violascens, antice fascia aut fasciola viridi-aurea; dorsulum saturatius aureum; scutellum 1. concolor 1. viride l. cyaneum*". Dahlbom (1854: 137) considered as *Chrysis germari* the only dimorphic male of this species, which is without teeth on the anal margin. He placed the male of *Chrysis germari* in his first "*Phalanx*". The description was based on one specimen received by Zetterstedt and still housed in his collection in Lund ("*Habitat in Europa meridionali rar.; in Croatia a D. Germar detecta* [the type]; *in insula Melita maris mediterranei a D. Antonio Schembri inventa, teste D. Zetterstedt qui specimen Melitense mihi dono dedit*"). Dahlbom’s interpretation of this species as *Chrysis germari* was confirmed by other authors (e.g. Eversmann 1857: 555). The only doubt relates to the third syntype; this specimen (not found in MNHU) with "*scutellum cyaneum*" could be related to any other species of the *succincta* group. However, the drawings to Dahlboms description (plate IX; figs. 101a, b, c) are clear and depict *Chrysis succinctula* with the main features of *Chrysis germari*.

We select from the above specimens the only specimen in good condition as a lectotype, since the second syntype is severly damaged and its metasoma is glued to the mesosoma. The lectotype is missing the last two flagellomeres of the right antenna.  The lectotype designation is given to prevent a future incorrect designation of the lectotype based on the third specimen not belonging to the syntype series; this specimen has a similar colour to *Chrysis succinctula* *sensu* Linsenmaier and might create confusion. We therefore propose *Chrysis succinctula* Dahlbom, 1854 as a **new synonym** of *Chrysis germari* Wesmael, 1839.

The species identified as *Chrysis succinctula* by Linsenmaier has to receive a new name. After having studied all possible synonyms of *Chrysis succincta* and related taxa, the first available name for this species is *Chrysis tristicula* Linsenmaier, 1959. Based on this research, we can summarize the results as follows:

- *Chrysis obtusiventris* Förster, 1853. Name included by Förster only in the key. The short description is enough to consider it as a valid name. Mocsáry (1889: 312) and Dalla Torre (1892: 99) placed it in doubt as a junior synonym of *Chrysis succincta*. It was later included it in the synonymic list of *Chrysis succincta* by Kimsey and Bohart (1991: 467). Since Förster (1853) did not give a complete description of this species, the type is lost, the locality is unknown, and no author used this name as a valid species, we suggest the suppression of this name. In a different paper, we will refer the case to the Commission on the ICZN.
- *Chrysis tarsata* Dahlbom, 1854. Two syntypes collected in Berlin, examined and housed at MNHU. The species is synonym of *Chrysis succincta* Linnaeus, 1767 *sensu* auctorum.
- *Chrysis minutula* Schenck, 1871. Mocsáry (1889: 312) placed *Chrysis minutula* in synonymy of *Chrysis succincta* and recognized that the type was a male and not a female. Dalla Torre (1892: 99) and Kimsey and Bohart (1991: 467) followed Mocsáry interpretation. However, the description of this species is clear and refers to a small male of *Chrysis germari* Wesmael. Schenck considered *Chrysis minutula* close to *Chrysis leachii*, which is characterized by a different thorax colour (mesonotum and scutellum contrasting with the posterior margin of the prothorax and the metanotum) compared with *Chrysis succincta*.
- *Chrysis aeneipes* Tournier, 1879. The species was described based on two syntypes (both males) collected at Peney (Geneva) on the 19^th^ July 1876 and 25^th^ of June 1878. The two syntypes have been examined and are still preserved in MHNG. They belong to *Chrysis germari* Wesmael. *Chrysis aeneipes* was already synonymized by Mocsáry (1889: 314). Kimsey and Bohart (1991: 468) placed it in synonym of *Chrysis succincta*.
- *Chrysis frivaldszkyi* Mocsáry, 1882. *Chrysis frivaldszkyi* was always considered as a valid species after Linsenmaier (1959). Kimsey and Bohart (1991:468) placed it in synonym of *Chrysis succincta*, but it was later revaluated by Strumia (1995: 5) and subsequent authors (e.g. Linsenmaier 1997, Rosa 2005). The lectotype of *Chrysis frivaldszkyi* was examined and it matches Linsenmaier’s (1959) interpretation. In the literature, this taxon is written in different ways: *Chrysis friwaldszkyi* (Berland and Bernard 1938), *Chrysis frivaldskyi* (Linsenmaier 1959, 1968; Strumia 1995), *Chrysis frivaldszkii* (Kimsey and Bohart 1991). The original spelling is *Chrysis frivaldszkyi*.
- Chrysis bicolor var. abeillei Frey-Gessner, 1887. Kimsey and Bohart (1991: 468) placed *Chrysis abeillei* as a synonym of *Chrysis succincta*. We could not find the type, but according to the description, the species is more closely related to *Chrysis bicolor* and *Chrysis illigeri*.
- Chrysis succincta var. sparsepunctata du Buysson (in André), 1892. This specimen is closely related to *Chrysis frivaldszkyi*, as correctly interpreted by Linsenmaier (1959). The type was examined and it is housed in ISEA-PAS.
- Chrysis succincta var. alicantina Mercet, 1904. *Chrysis alicantina* is a valid Iberian endemic species, correctly identified by Linsenmaier (1968, 1997), morphologically and chromatically different from *Chrysis succincta* and similar to *Chrysis chrysoscutella* Linsenmaier and *Chrysis germari* Wesmael.
- Chrysis succincta var. ignifacies Mercet, 1904. This is a valid Iberian endemic species, closely related to *Chrysis germari* Wesmael, but easily recognizible for the colour, the double punctuation on the metasoma, the length of the malar spaces and other morphological characteristics. *Chrysis ignifacialis* Linsenmaier, 1959 is an unnecessary replacement name for *Chrysis ignifacies* Mercet.
- Chrysis succincta var. hirsuta Trautmann, 1926 (*nec* Gerstaecker, 1869). The type of Chrysis succincta var. hirsuta is lost; however, based on the description and the collecting place it belongs to *Chrysis lucida* Linsenmaier, 1951.
- Chrysis succincta var. asiatica Trautmann, 1926 (*nec* Radoszkowski, 1889). The type is lost. Balthasar (1953: 286) replaced the name *asiatica* Trautmann with the new name *ferghanensis*. Based on the original description and the key given by Balthasar, the species resembles *Chrysis frivaldszkyi* and *Chrysis kesleri* Radoszkowski, described from the same area.
- Chrysis succincta var. germanica Trautmann, 1926. The type is lost but the description excludes any possible synonym with *Chrysis succincta*. Chrysis succincta var. germanica has evident teeth and a unique body colouration. In Northern Europe and Scandinavia, the only a similar species present is *Chrysis westerlundi*.
- Chrysis succincta var. pulcherrima Trautmann, 1926 (*nec* Lepeletier, 1806). Type lost, Balthasar (1953: 286) replaced the name *pulcherrima* with *perelegans*. Based on the description, it is clearly referrable to the female of *Chrysis albanica* Trautmann, 1927.
- Chrysis succincta var. virideocincta Trautmann, 1927 (based on the type of Chrysis succincta ab. virideocincta Hellén, 1919, unavailable). It is a synonym of *Chrysis bicolor* Lepeletier, 1806. The syntypes have been examined and Paukkunen et al. (2014) designated the lectotype.
- Chrysis succincta var. noskiewiczi Trautmann, 1927. The type is lost. The short description is related to a colour variation of *Chrysis frivaldszkyi*.
- Chrysis succincta var. transsylvanica Kiss-Endre, 1927. The type is housed in MNHM and was already placed in synonymy of *Chrysis albanica* by Móczár (1965).
- Chrysis succincta var. pannonica Hoffmann, 1935. The examination of the type material in NHMW confirmed that it is a synonym of *Chrysis frivaldszkyi* (Rosa 2009).
- Chrysis succincta var. decorata Hoffmann, 1937. Type not found. The description is clear and the species is phenotypically close to *Chrysis bicolor* or *Chrysis illigeri* bearing four visible teeth on the last visible tergite.
- Chrysis succincta var. komareki Balthasar, 1953. This species was already placed in synonymy of *frivaldszkyi* by Linsenmaier (1959). Kimsey and Bohart (1991) placed it in synonymy of *succincta* together with *frivaldszkyi*.
- Chrysis succincta f. pumilio Balthasar, 1953. It not even clear from the short description whether or not *pumilio* belongs to the *Chrysis succincta* or *Chrysis leachii* group. It could be conspecific with *Chrysis cypruscula* Linsenmaier, 1959.
- *Chrysis tristicula* Linsenmaier, 1959, **stat. n.** is a very variable species, due to its large distribution in the Mediterrean region. The southern Spanish and Moroccan specimens show a darker colouration of the metasoma. It varies from violet to bluish on the margin of the second and third tergites. In some cases, also the margin of the first tergite can be darker to violet or bluish. This particular colouration of the metasoma is not present in the French, Italian and Swiss specimens, even if a darker to violet reflection can be found on the anal margin of the third tergite, after the pit row. However, some African specimens show the colour of the metasoma without violet or bluish reflections (Linsenmaier 1999). The only morphological distinctive characteristics given by Linsenmaier to identify this species is the punctuation of the body "a little" finer and on the metasoma "a little" denser (*Pkt ein wenig feiner, auf Abd auch etwas dichter*) than *Chrysis succincta*. This characteristic is observed not only in the African specimens, but also in the Spanish ones. The Alpine specimens show a different punctuation, with sparse punctures decreasing in diameter on the second tergite, from the base to the apical margin, with shining intervals. However, the colour and the punctuation on the metasoma vary more or less gradually from the Alps to South Spain, but all the main morphological characteristics, from the length of the flagellomeres (with F-III longer than F-II) to the genital capsula, remain the same in all the examined specimens from the SW Mediterranean countries.

##### Current status.

*Chrysis germari* Wesmael, 1839.

**Plate 33.**
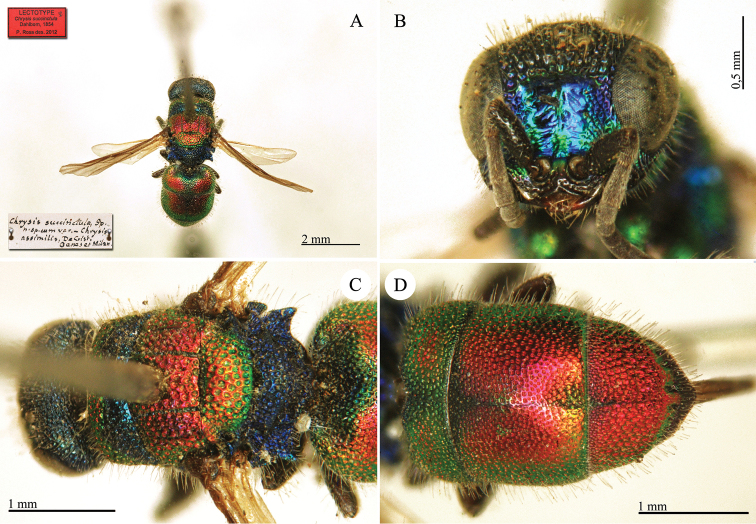
*Chrysis succinctula* Spinola, lectotype **A** Habitus, dorsal view **B** head, frontal view **C** mesosoma, dorsal view **D** metasoma, dorsal view.

#### Chrysis truncatella

Taxon classificationAnimaliaHymenopteraChrysididae

Dahlbom, 1854

Plate 34

Chrysis truncatella : Dahlbom 1854: 195.

##### Type locality.

"Habitat in Brasilia, Mus. Spinolae; qui specimen e Collectione Latreillei acceptum benigne communicavit".

##### Material.

**Holotype** ♀. *Chrysis truncatella*, Spin. inédit Coll. Latr. M. Buquet. Brésil.

**Catalogue Casolari & Casolari Moreno.** *Chrysis truncatella*, 1, 34, 1, 1 (box 51).

##### Current status.

*Caenochrysis parvula* (Fabricius, 1804) (synonymised and tranferred by Kimsey and Bohart 1991: 303).

**Plate 34.**
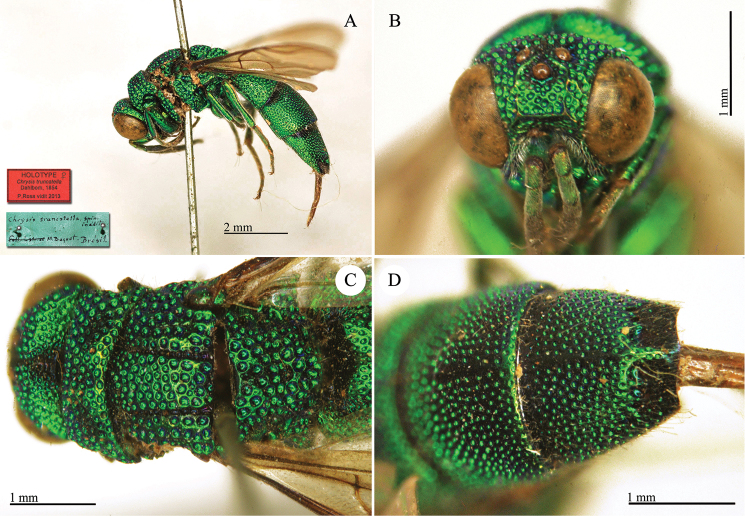
*Chrysis truncatella* Dahlbom, holotype **A** Habitus, lateral view **B** head, frontal view **C** mesosoma, dorsal view **D** second and third metasomal tergites, dorsal view.

#### Chrysis varicornis

Taxon classificationAnimaliaHymenopteraChrysididae

Spinola, 1838

Plate 35

Chrysis varicornis : Spinola 1838: 449.

##### Type locality.

Egypt.

##### Material.

**Holotype** ♂. *Chrysis varicornis*, Spin. M. Waltl, Égypte.

**Catalogue Casolari & Casolari Moreno.** *Chrysis varicornis*, 1, 23, 95, 2 (box 50).

##### Remarks.

Two specimens are found under the name *Chrysis varicornis* in the Spinola collection. One does not belong to the type series bearing the label: "*Espagne, M. Rambur*". The second specimen is likely the type but does not carry a label. However, the main label states: "*Chrysis varicornis, Spin. / M. Waltl. Egypte*". It matches the current interpretation of the species. It belongs to the *Chrysis radians* group.

##### Current status.

*Chrysura varicornis* (Spinola, 1838) (transferred by Kimsey and Bohart 1991: 497).

**Plate 35.**
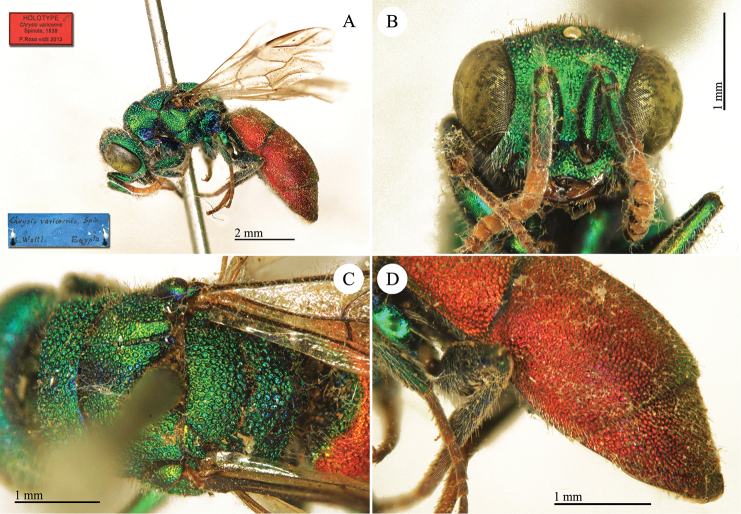
*Chrysis varicornis* Spinola, holotype **A** Habitus, lateral view **B** head, frontal view **C** mesosoma, dorsal view **D** metasoma, lateral view.

#### Chrysis versicolor

Taxon classificationAnimaliaHymenopteraChrysididae

Spinola, 1808

Plate 36

Chrysis versicolor : Spinola 1808: 241.

##### Type locality.

"Habitat passim in Liguria, haud infrequens".

##### Material.

**Lectotype** (here designated) ♀. *Chrysis versicolor*, Spin. Ins. Lig. Typus. *β* var *e* Coll. Latr. Genes [Genoa].

**Catalogue Casolari & Casolari Moreno.** *Chrysis versicolor*, 1, 110, 0, 2 (box 51).

##### Remarks.

Only two specimens are found unter the name *Chrysis versicolor* in the Spinola collection. One is not a syntype, since it bears a rounded label (6295) and was acquired with other specimens of the Latreille collection. The second specimen is labelled "Type", and it was collected at Genoa, as written on the main label. Spinola’s description is surely based on a syntype series ("*haud infrequens*"), therefore we here designate as lectotype the last syntype left in the collection, even though the specimen is badly damaged. It lacks the metasoma, the right flagellum and almost all of the left flagellum; only the right mesoleg is complete. However, many diagnostic characteristics are still visible and even the sex is identifiable.

##### Current status.

*Spintharina versicolor* (Spinola, 1808) (transferred by Kimsey and Bohart 1991: 558).

**Plate 36.**
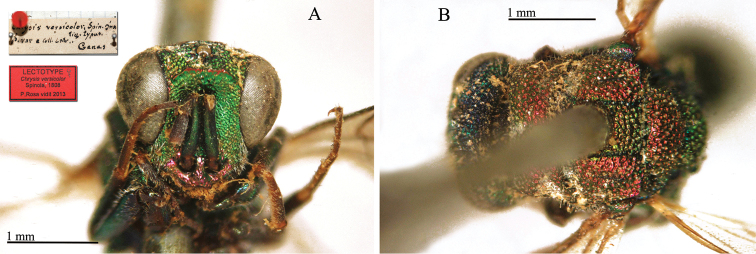
*Chrysis versicolor* Spinola, lectotype **A** Head, frontal view **B** mesosoma, dorsal view.

#### Elampus gayi

Taxon classificationAnimaliaHymenopteraChrysididae

Spinola, 1851

Plate 37

Elampus gayi : Spinola 1851: 413.

##### Type locality.

Chile. "De Santa Rosa y de la Ligua".

##### Material.

**Lectotype** (here designated) ♀. *Elampus Gayi*, Spin. / D. Gay, Chili. // **Paralectotype** 1♀. *Elampus Gayi* Spinola, det. L. D. French.

**Catalogue Casolari & Casolari Moreno.** *Elampus gayi*, 1, 52, 32, 1 (box 50).

##### Remarks.

French labelled the specimen as paralectotype, but the designation was not published. The species was described based on at least two males (*sexo dudoso*) collected at Santa Rosa and Ligua. The second syntype is housed in MNHN (du Buysson 1898: 519). Du Buysson completed the description of this species, even though the specimen was missing the head and part of metasoma. Later, du Buysson (1899: 160) removed this specimen from the catalogue of the types housed in the MNHN. Since the syntype in MNHN is badly damaged and it was not considered as a type by du Buysson (1899), we here designate the syntype of *Elampus gayi* as lectotype in the Spinola collection, according to the Recommendation 74D of the Code. The specimen is in good condition, even though it is missing the last three flagellomeres of the left antenna and five of the right antenna.

##### Current status.

*Elampus gayi* Spinola, 1851.

**Plate 37.**
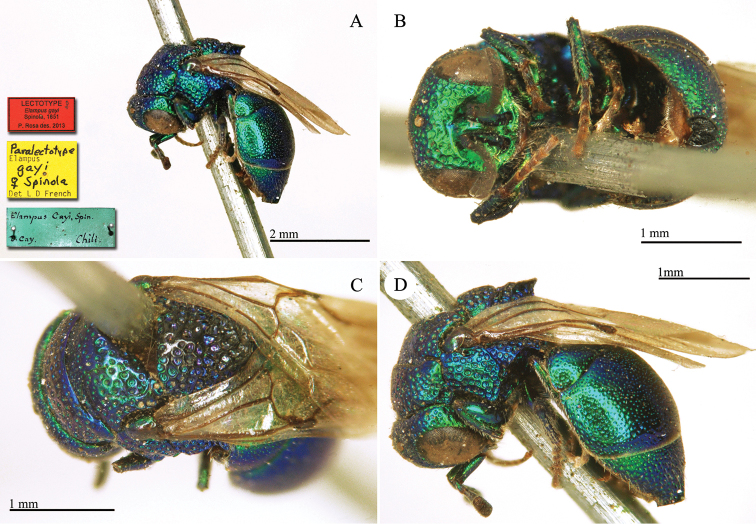
*Elampus gayi* Spinola, lectotype **A** Habitus, lateral view **B** head, and anal margin of the last tergite, frontal view **C** mesosoma, dorsal view **D** habitus, lateral view.

#### Euchroeus candens

Taxon classificationAnimaliaHymenopteraChrysididae

Dahlbom, 1854

Plate 38

Euchroeus candens : Dahlbom 1854: 371 *nec* Germar, 1817.

##### Type locality.

"Habitat in Africa ad promontorium bonae spei; Mus. Dom. Spinola".

##### Material.

**Holotype** ♀. *Euchroeus candens*, Kl. / D. Klug, Cap. B. Esp.

**Catalogue Casolari & Casolari Moreno.** Euchraeus (sic) candens, 132, 53, 49, 1 (box 52).

##### Remarks.

In Kimsey and Bohart (1991: 297), under the name *Brugmoia* [= *Euchroeus*] *torrida* (Mocsáry, 1889). The generic name *Euchroeus* Latreille was conserved by the International Commission on Zoological Nomenclature (ICZN, Opinion 1906). The name *candens* was considered as a junior secondary homonym of *Chrysura candens* Germar, 1817 (currently *Chrysura candens*) by Mocsáry (1889). Since the two respective species have been considered as belonging to different genera after 1889, the name *Euchroeus candens* must be considered as a valid name (Madl and Rosa 2012: 84).

##### Current status.

*Euchroeus candens* Dahlbom, 1854.

**Plate 38.**
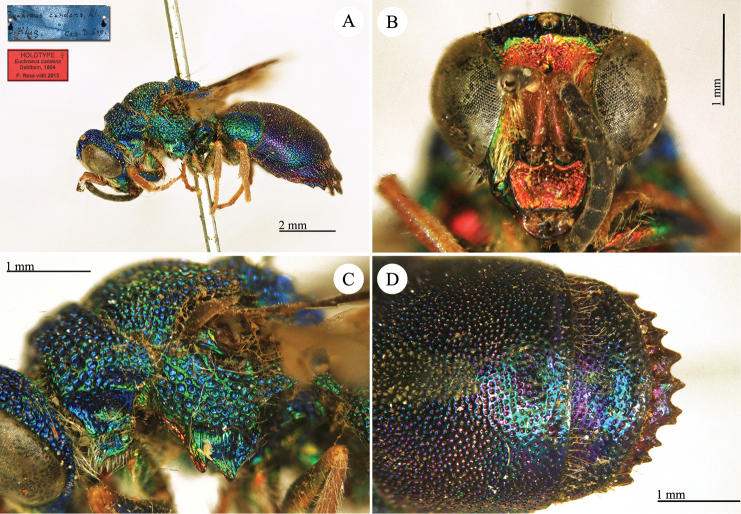
*Euchroeus candens* Dahlbom, holotype **A** Habitus, lateral view **B** head, frontal view **C** mesosoma, lateral view **D** second and third metasomal tergites, dorsal view.

#### Euchroeus coerulans

Taxon classificationAnimaliaHymenopteraChrysididae

Dahlbom, 1854

Euchroeus coerulans : Dahlbom 1854: 372 *nec* Fabricius, 1804.

##### Type locality.

"Habitat in Africa ad promontorium bonae spei, Mus. Dom. Spinola".

##### Material.

**Holotype** (sex unknown): *Euchroeus coerulans*, Kl. // D. Klug, Cap. B. Esp.

**Catalogue Casolari & Casolari Moreno.** *Euchraeus coerulans*, 132, 53, 49, 1 (box 52).

##### Remarks.

The type is badly damaged, having no head and metasoma, (except first tergite, which is still present).

##### Current status.

*Euchroeus candens* Dahlbom, 1854 (synonymised by Mocsáry 1889: 600).

#### Hedychrum brasilianum

Taxon classificationAnimaliaHymenopteraChrysididae

Dahlbom, 1854

Plate 39

Hedychrum brasilianum : Dahlbom 1854: 59.

##### Type locality.

"Habitat in Brasilia: D. Buquet, Mus. Spinolae".

##### Material.

**Holotype** ♂. *Hedychrum brasilianum* Spin. D. Klug. Brasil // **Holotype** *Hedychrum brasilianum* ♂ Dahlbom det. L. D. French.

**Catalogue Casolari & Casolari Moreno.** *Hedychrum brasilianum*, 1, 34, 1, 1 (box 50).

##### Remarks.

The type is in bad condition. It lacks the head, forelegs, right mesoleg, and the tarsi of the right metaleg. The ventral surface of the metasoma is missing due to a dermestid attack.

##### Current status.

*Hedychrum brasilianum* Dahlbom, 1854.

**Plate 39.**
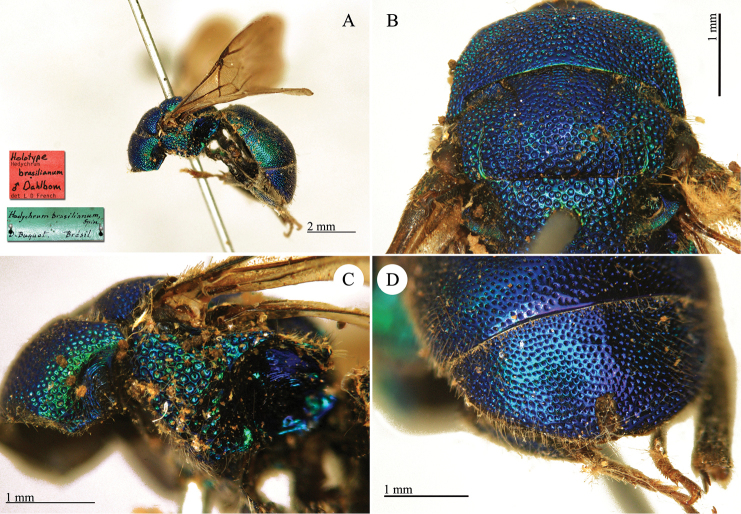
*Hedychrum brasilianum* Dahlbom, holotype **A** Mesosoma and metasoma, lateral view **B** mesosoma, dorsal view **C** mesosoma, lateral view **D** third metasomal tergite, dorsal view.

#### Hedychrum caerulescens

Taxon classificationAnimaliaHymenopteraChrysididae

Lepeletier, 1806

Hedychrum caerulescens : Lepeletier 1806: 122.

##### Type locality.

"Mâle et femelle. Donné par M. Walkenaer qui l’a trouvée aux environs de Paris".

##### Material.

**Lectotype** (here designated) ♂. *Hedychrum caeruleus* (*Chrysis*) Deg. [De Geer] – (*Omalus*) Dhlbm. *Hedychrum caerulescens* Lepel. 122 coll. St. Fargeau, Parisiae.

**Catalogue Casolari & Casolari Moreno.** *Hedychrum caeruleus*, 36, 185, 53, 1 (box 50).

##### Remarks.

Lepeletier (1806) described *Hedychrum caerulescens* based on two specimens, one male and one female. One of these two syntypes was acquired by Spinola and belongs to the species *Pseudomalus violaceus* (Scopoli, 1763); the second syntype is housed at MNHN. Du Buysson (1898: 563) listed one specimen in Lepeletier’s collection *Hedychrum coerulescens* = *Ellampus caeruleus* (De Geer), but it is not listed as a type. We examined the second syntype (currently in the general collection MNHN box 8). It belongs to the same species, *Pseudomalus violaceus* (Scopoli), even though it is placed under the name of *Omalus aeneus* (Fabricius).

We designate as lectotype of *Hedychrum caerulescens* Lepeletier the syntype male. The reason is to fix the synonym with *Pseudomalus violaceus* (Scopoli). The specimen is damaged, lacking its fore wings and left hind wing, and it is partially covered by mould.

##### Current status.

*Pseudomalus violaceus* (Scopoli, 1763) (synonymised by Dalla Torre 1892: 19; transferred by Kimsey and Bohart 1991: 270).

#### Hedychrum chloroideum

Taxon classificationAnimaliaHymenopteraChrysididae

Dahlbom, 1854

Hedychrum chloroideum : Dahlbom 1854: 66 (given as var. *b*).

##### Type locality.

"Habitat in Europa media et meridionali mensibus Majo - Julio passim; Turcia: D. Drewsen; Graecia: D. Loew; Austria: D. D. De Christophori, Kollàr et Megerle von Mühlfeld; Silesia: D. Zeller".

##### Material.

**Lectotype** (here designated) ♂. *Hedychrum chloroideum* // (*Chrysis*) Ziegl. // D. De Cristofori, Autriche.

**Paralectotypes** 3 ♂♂. idem.

**Catalogue Casolari & Casolari Moreno.** *Hedychrum chloroideum*, 84, 9, 19, 4 (box 50).

##### Remarks.

The specimens of *Hedychrum chloroideum* collected in Austria by Ziegler and listed as syntypes by Dahlbom (1854: 66) are still housed in the Spinola collection. The syntype series given by Dahlbom includes specimens collected in Turkey (coll. Drewsen, ZMUC), Greece (coll. Loew), Austria (coll. De Christophori, Kollár, Megerle, MRSN), and Silesia (coll. Zeller, LZM). The original description is based only on males, characterized by the green colour, sometimes light green to blue-green. The name *chloroideum* is derived from this particular colouration. The female is easily recognizable by the shape and the colouration of its body. It has an elongated metasoma and red-purple colour on head and on the dorsal part of the mesosom. The metasoma, the propodeum, and ventral surface are a contrasting blue colour. In Dahlbom’s time, the female was known as *Hedychrum fervidum* (Fabricius). Males and females were considered as different species because of the remarkable sexual dimorphism and dichroism. *Holopyga chloroidea* (Dahlbom, 1854) as well as *Holopyga curvata* (Förster, 1853) (name with priority) had been considered as a valid species for a very long time. The hypothesis given by Trautmann (1922: 321) that *Holopyga curvata* (= *Holopyga chloroidea*) could be the male of *Holopyga fervida* was not immediately accepted (e.g. Invrea, 1923: 13). Once the synonym was accepted, all the authors agreed on this fact, except Linsenmaier. Linsenmaier (1959, 1968, 1969) considered *Holopyga chloroidea* as a separate subspecies of *Holopyga fervida* distributed in Asia Minor, Syria, Palestine, and Cyprus. His interpretation, even though not in contrast with the original distribution of *Holopyga chloroideum* as outlined by Dahlbom, can be a source of taxonomical instability. This is because the oriental form of *Holopyga fervida*, exhibiting a coarser punctuation, is currently referred to as Holopyga fervida ssp. buyssoni Mercet, 1902. To retain nomeclatural stability, we designate a lectotype of *Hedychrum chloroideum* Dahlbom that clearly does not refer to this subspecies. We select the specimen from the above three mentioned ones that is only partially damaged, lacking the right flagellum, tibia and tarsi of the left foreleg, femur, tibia and tarsi of the mid and left metaleg.

Kimsey (1986: 108) designated a lectotype of *Hedychrum chloroideum* Dahlbom at MNHN, but the designation was based on a female collected in France ("env. de Paris") and found in the Lepeletier collection. The original description is based only on males and no syntype was collected in France or was housed in Lepeletier’s collection. This specimen is not a syntype and therefore it cannot be considered as a lectotype, according to the Art. 74.2.

##### Current status.

*Holopyga fervida* (Fabricius, 1781) (synonymised by Trautmann 1922: 321).

#### Hedychrum coelestinum

Taxon classificationAnimaliaHymenopteraChrysididae

Spinola, 1838

Hedychrum coelestinum : Spinola 1838: 454.

##### Type locality.

"Egypte".

##### Material.

**Holotype** (?) ♀. *Hedychrum coelestinum* Kl. D.D. Waltl Égypte et Klug, Cap. B. Esp. // **Paralectotype** *Hedychrum coelestinum* ♀ Spinola det. L. D. French.

**Catalogue Casolari & Casolari Moreno.** *Hedychrum coelestinum*, 132, 23/53, 95, 2 (box 50).

##### Remarks.

In the Spinola collection, two specimens from Egypt (D. Waltl) and South Africa (Cap B. [onne] Esp. [érance]" (D. Klug)) referring to this species are present. Only one specimen belongs to the original type series. However, it is currently impossible to state which one, since both specimens lack locality labels. At present, we cannot identify the specimen that had been collected in Egypt. Both specimens were examined by Dahlbom (1854: 60) and they are found under the name "*Hedychrum cœlestinum* Kl.", a species never described by Klug. Dahlbom (1854) erroneusly assigned this species to Klug and not to Spinola, even though he knew Spinola’s (1838) paper. Furthermore, Dahlbom (1854) named this species "*caelestinum*", which we consider an incorrect subsequent spelling (Madl and Rosa 2012: 96). The two specimens were labelled as lectotype and paralectotype by L.D. French, but the lectotype designation has not been published. The two females have different colours: one is greenish and the second is a deep blue. The blue one was labelled by French as paralectotype, but the colour matches Spinola’s description ("*La couleur du corps est d’un bleu plus intense*") and we suppose that this one could be the type. In Dahlbom’s collection in LZM, there is another specimen labelled as type by a former curator, not by Dahlbom himself, and that refers to the specimen listed as "*caelestinum* Kl." (Dahlbom 1854: 60).

##### Current status.

*Hedychrum coelestinum* Spinola, 1838.

#### Hedychrum difficile

Taxon classificationAnimaliaHymenopteraChrysididae

Spinola, 1851

Plate 40

Hedychrum difficile : Spinola 1851: 410.

##### Type locality.

Chile.

##### Material.

**Lectotype** (here designated) ♀. *Hedychrum difficile*, Spin. D. Gay, Chili. // **Paralectotype** *Hedychrum difficile* ♂ Spinola det. L. D. French.

**Paralectotype** (sex unknown): idem // **Paralectotype** 1 ♂. *Hedychrum difficile* Spinola, det. L. D. French.

**Catalogue Casolari & Casolari Moreno.** *Hedychrum difficile*, 1, 52, 32, 2 (box 50).

##### Remarks.

Spinola (1851: 411) described *Hedychrum difficile* on various female specimens, and at least one specimen considered as a possible male ("*Macho dudoso*", "*Uno solo de los individuis cojidos por M. Gay está en este caso*."). In the Spinola collection there are two specimens collected in Chile by Gay matching the original description and bearing French’s paralectotype labels. The lectotype designation has not been published, however. French (1985: 622) wrote that he examined the female holotype in MNHN; this information was later reported in Kimsey and Bohart (1991: 174). *Hedychrum difficile* is a common species in Chile and the specimen found at MNHN (box number 16 of the general collection) is a syntype as well. Du Buysson (1899: 161, sub *Hedychridium*) listed the specimen in MNHN without any type status.

We designate a lectotype of *Hedychrum difficile*. It is a female (not male, as reported on the label) in perfect condition; the second specimen, the paralectotype, is badly damaged, being without metasoma, legs and two wings. We prefer to designate the specimen in MRSN because it is based on a specimen housed in the collection of the describing author (Recommendation 74D of the Code).

##### Current status.

*Exallopyga difficile* (Spinola, 1851) (transferred by French 1985: 622).

**Plate 40.**
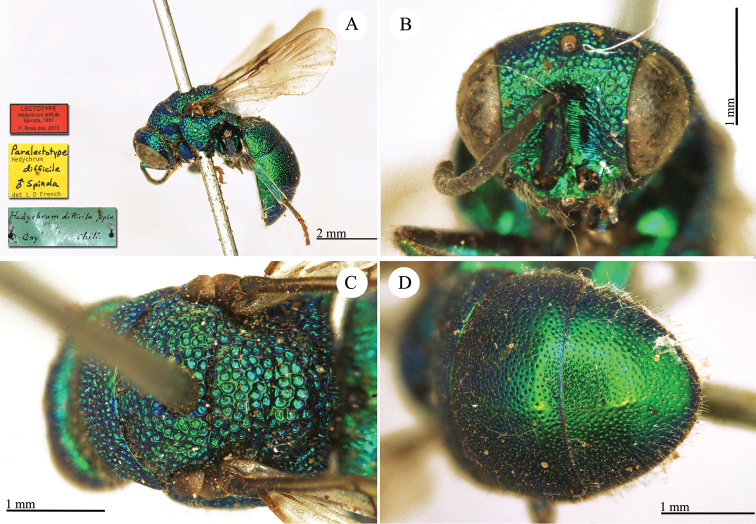
*Hedychrum difficile* Spinola, lectotype **A** Habitus, lateral view **B** head, frontal view **C** mesosoma, dorsal view **D** second and third metasomal tergites, dorsal view.

#### Hedychrum minutum var. duponti

Taxon classificationAnimaliaHymenopteraChrysididae

Dahlbom, 1854

Hedychrum minutum var. duponti : Dahlbom 1854: 83. (given as *Hedychrum minutum* var. *c*).

##### Type locality.

"Exemplar *varietatis c* e Mexico a D. Dupont reportatum in Museo Excell. Spinolae (*Hedychrum Duponti* Spin." nominatum) examinavi".

##### Material.

**Holotype** (sex unknown): *Hedychrum Duponti* Spin. / *Hedychrum Minutum* var. Dhlbm / D. Dupont. Mexico.

**Catalogue Casolari & Casolari Moreno.** *Hedychrum duponti*, 1, 154, 23, 1 (box 50).

##### Remarks.

The type is closely related to *Hedychridium krajniki* Balthasar, 1946. The type locality could be in error.

##### Current status.

*Hedychridium duponti* Dahlbom, 1854.

#### Hedychrum incrassatum

Taxon classificationAnimaliaHymenopteraChrysididae

Dahlbom, 1854

Plate 41

Hedychrum incrassatum : Dahlbom 1854: 73.

##### Type locality.

"Habitat in Sicilia: D. Chiliani (sic), Mus. D. Spinolae".

##### Material.

**Holotype** ♂. *Hedychrum incrassatum* / Spin. - inedit; D. Ghiliani, Sicile. // **Holotype** *Hedychrum incrassatum* ♂ Spinola det. L. D. French.

**Catalogue Casolari & Casolari Moreno.** *Hedychrum incrassatum*, 1, 204, 33, 1 (box 50).

##### Current status.

*Hedychridium incrassatum* (Dahlbom, 1854) (transferred by du Buysson (in André), 1891: 188).

**Plate 41.**
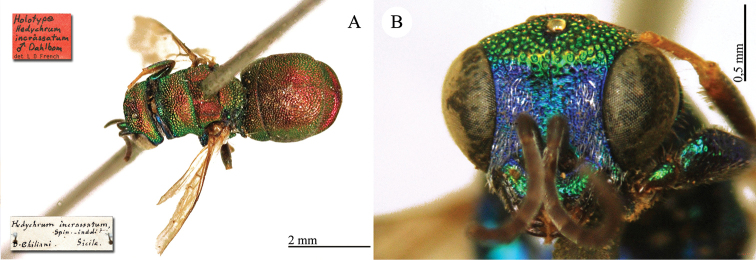
*Hedychrum incrassatum* Dahlbom, holotype **A** Habitus, dorsal view **B** head, frontal view.

#### Hedychrum rutilans

Taxon classificationAnimaliaHymenopteraChrysididae

Dahlbom, 1854

Hedychrum rutilans : Dahlbom 1854: 76.

##### Type locality.

"Habitat in Anglia, Germania, Gallia, Hispania, passim".

##### Material.

**Syntypes** 2 ♂♂, ♀. *Hedychrum rutilans*, Meg. var. c, Dhlbm. - *Hedychrum regium*, Lep. Liguria.

**Catalogue Casolari & Casolari Moreno.** *Hedychrum rutilans*, 186, 145, 0, 3 (box 50).

##### Remarks.

Dahlbom (1854) described *Hedychrum rutilans* based on a syntype series subdivided into three varieties (var. *a*, var. *b*, var. *c*). The syntypes listed by Dahlbom are housed in the following museums: *Hedychrum rutilans* var. *a*: NHMW (*teste* Kollar), MNHU (*teste* Klug), ZMUC (*teste* Drewsen); *Hedychrum rutilans* var. *b*: MNHU (*teste* Klug); *Hedychrum rutilans* var. *c*: MRSN (Spinola coll., "*Hedychrum regium* Pellet. secund. Spin. in litt." e "*Hedychrum intermedium* Mus. Spinolae"). These syntypes are still housed in the listed museums. Morgan (1984: 10) designated the lectotype on a specimen collected by Zeller and housed in the Dahlbom Collection at LZM. This specimen is labelled: "*Hedychrum rutilans* Megerl. Dahl. var. *a*". According to the Art. 74.2 of the Code this specimen is not a syntype, therefore it loses its status of lectotype.

The name *Hedychrum rutilans* is a matter of conflict between entomologists. The history of the names *Hedychrum rutilans* and *Hedychrum intermedium* is long and complicated. Linsenmaier (1959, 1968, 1997a, 1997b, 1999) used the name *Hedychrum intermedium* Dahlbom, 1845, instead of *Hedychrum rutilans* Dahlbom, 1854, as many other authors did in the past. In various European collections, specimens belonging to this species are still found under the name *Hedychrum intermedium*. Many entomologists, in fact, still follow Linsenmaier’s interpretation. Linsemaier never accepted the synonymy proposed by Morgan (1984: 8) and that was accepted by Kimsey and Bohart (1991). Morgan (1984), in fact, discovered that the holotype of *Hedychrum intermedium* belongs to the genus *Holopyga*. Unfortunately, Morgan did not provide any further information on the species nor in which museum he examined this type. After an extensive tour in the European museums, we found out that the type of *Hedychrum intermedium* is housed in LZM. Kimsey and Bohart (1991: 232) wrote that they examined this holotype at MNHN, and later Linsenmaier (1997a) argued that Dufour’s specimens are housed at MNHN. After studying all relevant type material at MNHN and after having conducted an extensive literature survey, we are confident in writing that not one specimen labelled "*Holopyga intermedia Gall. Dufour*","*Holopyga intermedia*" or "*Hedychrum intermedium*" is housed at MNHN. In the "General collection" in MNHN there are two specimens labelled "*Hedychrum rutilans*" and "*Coll. Dufour 1834*". These two specimens had not been studied by Morgan, yet they were listed by du Buysson (1898: 521) and mentioned as possible "types" by Linsenmaier (1997a), since their labels match the original data cited by Dahlbom (1845). Based on the erroneous information given by Kimsey and Bohart (1991) on the type depository, Linsenmaier (1997a) did not accept Morgan’s interpretation and stated that labeles must have been exchanged. Moreover, Linsenmaier argued that it was not possible that Dahlbom, who described the genus *Holopyga* in the same paper, would have confused it with *Hedychrum*.

At the beginning of our studies, we agreed with Linsenmaier and we also noticed that no other European *Hedychrum* has the described colour "♂ *thorax antice viridis postice cyaneus*"; only the male of *Holopyga ignicollis* *sensu* Linsenmaier (= *Holopyga aureomaculata* Abeille) shows a similar colouration. We concluded that Morgan probably confused the type of *Hedychrum intermedium* with the type of another mysterious species described in the same work by Dahlbom on Dufour material collected in France: *Holopyga nitidula*. In this sense, the examination of the Dahlbom collection in LZM was fundamental. The specimen cited by Morgan is indeed the type of *Hedychrum intermedium*. This confirmation is not only based on the precise labels, already cited by Morgan, but also on the morphological and chromatic characteristics given by Dahlbom. This specimen is a male of *Holopyga fervida* (Fabricius, 1781) with colouration similar to Holopyga fervida var. taorminensis Trautmann: pronotum and mesonotum light bluish-greenish, in contrast with the rest of the mesosoma. But the most important characteristic is the punctuation on the mesosoma: ‘*pronotum et dorsulum nitida sparse punctata*’. No *Hedychrum* species has this peculiar punctuation, but *Holopyga fervida* has it.

It is not strange that Dahlbom in 1845 identified the male of *Holopyga fervida* as *Hedychrum*. In fact, Dahlbom in 1854 described again the males of *Holopyga fervida* as *Hedychrum chloroideum*, based on specimens entirely green or bluish-green, without any contrasts in the colouration of the mesosoma.

It seems that Linsenmaier was influenced by Richards (1935: 158). This important author received the type of *Hedychrum intermedium* and *Hedychrum rutilans* by Kemner (Lund): "*Through the kindness of Dr. N. A. Kemner, we have examined the type of* Hedychrum rutilans *Dahlbom, 1854. It is a female bearing two labels (1)" Z. Mer."or"L. Mer."and (2)*" Hedychrum rutilans. *Megerl. Secund. M.B. Dhbm. var. a."The specimen agrees with Dahlbom’s description of his var. a. (i.e. the typical form of the species) and also with the modern interpretation of his name (e.g. Trautmann, 1928). Dr. Kemner also sent what is almost certainly the type of* Hedychrum intermedium *Dahlbom, 1845. This species was described in 1845 from France but in 1854 Dahlbom dropped the name* intermedium*; his var. c. of* Hedychrum rutilans *agrees with the earlier described intermedium. The probable type of* intermedium *is a male bearing two labels: (1)*" Hedychrum rutilans *Dhlbm. var. c."and (2) " Hab.? Fontainebleau Collect. Barbut."This specimen agrees with the original description of* intermedium. *It is a male of one of the greenish forms of* Hedychrum rutilans. *It may be described as follows: - Green; slight trace of copper on central lobe of mesonotum. Postscutellum and propodeum blue. Abdomen green, disc of second tergite and whole of third, copper-tinged. Legs blue. Venter of abdomen black. In my opinion, therefore, the species should be known as* Hedychrum intermedium *Dahlbom, 1845*".

Nevertheless, Richards did not realize that none of the examined specimens was truly a type. In particular, *Hedychrum intermedium* did not match the original type, since it was collected at Fontainebleau by Barbut and not by Dufour. This should be the reason different authors, including Linsenmaier, considered *Hedychrum intermedium* had priority over the name *Hedychrum rutilans*.

In conclusion, we formally propose here the new synonymy: *Hedychrum intermedium* Dahlbom, 1845 = *Holopyga fervida* (Fabricius, 1781). The valid name for one of the most common European species is therefore *Hedychrum rutilans* Dahlbom, 1854, as already stated by Morgan (1984).

##### Current status.

*Hedychrum rutilans* Dahlbom, 1854.

#### Hedychrum virens

Taxon classificationAnimaliaHymenopteraChrysididae

Dahlbom, 1854

Plate 26C

Hedychrum virens : Dahlbom 1854: 74.

##### Type locality.

"Habitat in Rossia meridionali et Lusitania forte rarissime".

##### Material.

**Lectotype** (here designated) ♂. *Hedychrum virens* Kl. Dlbm ♀. Coll. Rambur. Russ. mer. // Sud Russ.

**Catalogue Casolari & Casolari Moreno.** *Hedychrum virens*, 135, 199, 74, 1 (box 50).

##### Remarks.

Dahlbom (1854: 74) described *Hedychrum virens* based on two specimens, one male and one female: the male was collected in Portugal (Lusitania) and housed in MNHU; the female was collected in Southern Russia and housed in the Spinola collection. We found that the specimen in the Spinola collection is a male and not a female. We designate this specimen as lectotype of *Hedychrum virens* since it matches the current interpretation of the species. It is in perfect condition and it is prepared with open wings. It is possible that the Iberian population could be considered as a separate subspecies. The species shows a peculiar distribution, and in Western Europe is found only on the Iberian Peninsula. The rest of its known distribution extends from Greece over the Middle East to central Asia. There are some old records from Italy collected in the 19th century in various museums (MRSN, MHNG, MCZ).

##### Current status.

*Hedychrum virens* (Dahlbom, 1854).

#### Holopyga janthina

Taxon classificationAnimaliaHymenopteraChrysididae

Dahlbom, 1854

Plate 42

Holopyga janthina : Dahlbom 1854: 50.

##### Type locality.

"Habitat in Africa, ad Promontorium Bonae Spei a D. Westermann detecta et ibidem a D. Draege revisa. Museis D. D. Westermann et Spinola".

##### Material.

**Lectotype** (here designated) ♀. *Hedychrum janthinum* (*Holopyga*) Dahlb. - Dr D. Draege. Cap. B. Esp. // **Lectotype** *Holopyga janthina* ♀ Dahlbom det. L. D. French.

**Catalogue Casolari & Casolari Moreno.** *Hedychrum janthinum*, 27, 53, 21, 1 (box 50).

##### Remarks.

Dahlbom (1854: 50) described *Holopyga janthina* based on a series of specimens received by Westermann and Draege. These syntypes are now housed in MRSN and ZMUC. French pinned a lectotype label, but the designation has not been published. We designate a lectotype of *Holopyga janthina* Dahlbom, using the same syntype that was selected by French, to fix the current interpretation of the species. The head of the lectotype is broken and glued on a white label; the right antenna is without the last six flagellomeres, the left one is without the last seven flagellomeres, the right foreleg is missing the tibia and tarsi; the right mesoleg and the forelegs are without the last tarsi.

##### Current status.

*Holopyga janthina* Dahlbom, 1854.

**Plate 42.**
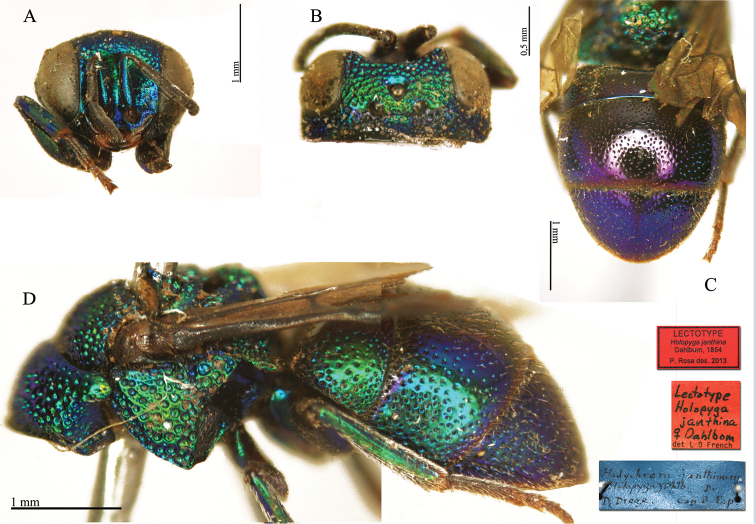
*Holopyga janthina* Dahlbom, lectotype **A** Head, frontal view **B** head, dorsal view **C** metasoma, dorsal view **D** mesosoma and metasoma, lateral view.

#### Holopyga luzulina

Taxon classificationAnimaliaHymenopteraChrysididae

Dahlbom, 1854

Plate 43

Holopyga luzulina : Dahlbom 1854: 49.

##### Type locality.

"Habitat in Brasilia, D. D. Milde et Spinola, qui specimina benevole communicarunt".

##### Material.

**Lectotype** (here designated) ♂. *Hedychrum lazulinum* (*Holopyga*) Dahlb. - M.B. Klug. D. Klug. Brasil // **Lectotype** ♂. *Holopyga lazulina* Dahlbom, det. L. D. French.

**Catalogue Casolari & Casolari Moreno.** *Hedychrum lazulinum*, 27, 34, 49, 2 (box 50).

##### Remarks.

Dahlbom (1854: 49) described *Holopyga luzulina* based on two specimens received from Milde and Spinola. French labelled the specimen in the Spinola collection as lectotype, but the designation has not been published. Since we could not find the second syntype in MNHU, we designate the lectotype of *Holopyga luzulina* Dahlbom to fix the current interpretation of the species. After Dahlbom, many South American *Holopyga* have been described, and there is the possibility that the other syntype belongs to a different species, as found in other cases (e.g. *Holopyga dohrni* Dahlbom, 1854, with different syntypes from U.S.A. and Cuba). The type is missing tibia and tarsi of the right foreleg and left mesoleg and also the femora, tibiae, and tarsi of the left metaleg; a thin mould layer covers the right side of the body. The second specimen placed under the name *Holopyga luzulina* is a male of *Hedychrum rutilans* Dahlbom, 1854, most likely placed in this position in recent times.

##### Current status.

*Holopyga luzulina* Dahlbom, 1854.

**Plate 43.**
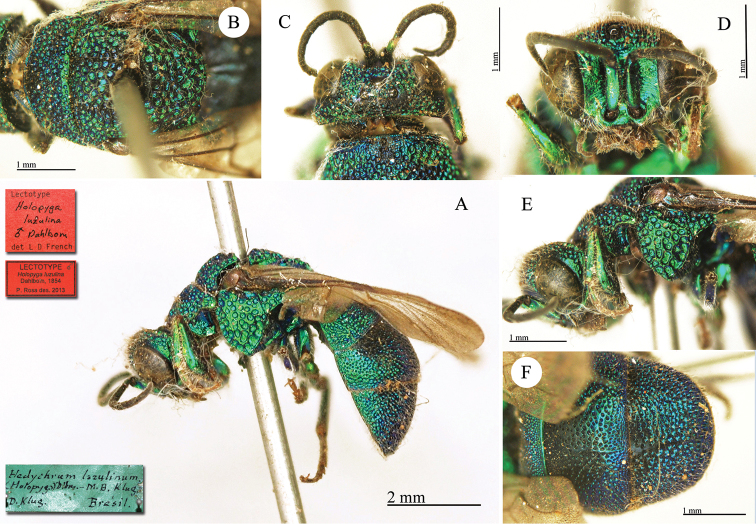
*Holopyga luzulina* Dahlbom, lectotype **A** Habitus, dorsal view **B** Mesosoma, dorsal view **C** head, dorsal view **D** head, frontal view **E** head and mesosoma lateral view **F** second and third metasomal tergites, dorsal view.

#### Holopyga ovata

Taxon classificationAnimaliaHymenopteraChrysididae

Dahlbom, 1854

Holopyga ovata : Dahlbom 1854: 51.

##### Type locality.

"In Insulis Archipelagi: D. Loew; Italia: D. Spinola; Austria: D. Kollàr; Prussia: D. D. Dohrn et Lüben; Svecia: Scania (ad Esperöd d. 12. Augusti 1838), – Ostro-Gothia (in monte Omberg d. 22. Juli 1835 in copula cum var. c!) et Gottlandia (in pratis Gothem d. 18. Juli 1841) mihi obvia".

##### Material.

**Syntype** 1 ♀. *Hedychrum ovatum* (*Chrysis*) Pallas – (*Holopyga*) Dahlb. var. B – *Hedychrum regium* olim Lombardia.

**Catalogue Casolari & Casolari Moreno.** *Hedychrum ovatum*, 199, 144, 0, 1 (box 50).

##### Remarks.

The syntype is badly damaged; the head and metasoma are glued on a separated label pinned in box 52.

##### Current status.

*Holopyga generosa* (Förster, 1853) (synonymised by Linsenmaier 1987: 135).

#### Parnopes denticulatus

Taxon classificationAnimaliaHymenopteraChrysididae

Spinola, 1838

Plate 44

Parnopes denticulatus : Spinola 1838: 455.

##### Type locality.

Egypt.

##### Material.

**Holotype** ♂. *Parnopes denticulatus* Spin. / D. Waltl Égypte.

**Catalogue Casolari & Casolari Moreno.** *Parnopes denticulatus*, 1, 23, 0, 1 (box 52).

##### Remarks.

The type is badly damaged, without the dorsal and frontal part of the head; only the occipital part as far as the mandible complex, the clypeus and antennae are left.

##### Current status.

*Cephaloparnops denticulatus* (Spinola, 1838) (transferred by Kimsey and Bohart 1991: 578).

**Plate 44.**
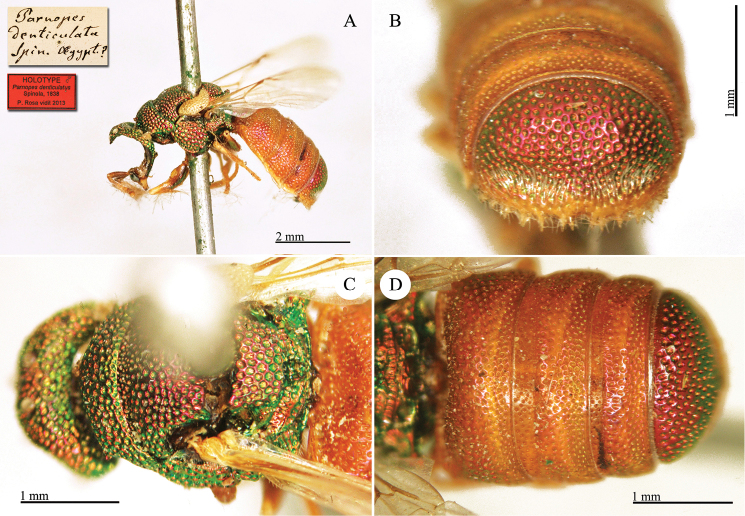
*Parnopes denticulatus* Spinola, holotype **A** Habitus, lateral view **B** fourth metasomal tergite, posterior view **C** mesosoma, dorsal view **D** metasoma, dorsal view.

#### Parnopes fischeri

Taxon classificationAnimaliaHymenopteraChrysididae

Spinola, 1838

Plate 45

Parnopes fischeri : Spinola 1838: 455.

##### Type locality.

Egypt.

##### Material.

**Holotype** ♀. *Parnopes Fischeri*, Spin.; Égypte ?

**Catalogue Casolari & Casolari Moreno.** *Parnopes fischeri*, 1, 23, 91, 1 (box 52).

##### Current status.

*Parnopes fischeri* Spinola, 1838.

**Plate 45.**
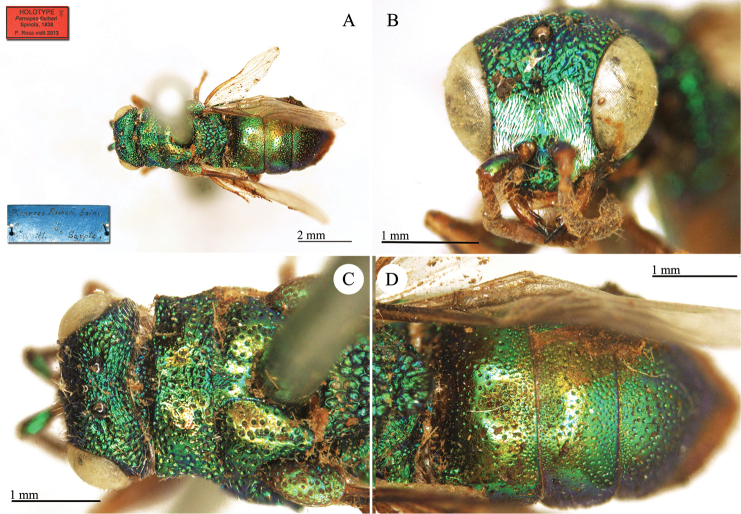
*Parnopes fischeri* Spinola, holotype **A** Habitus, dorsal view **B** head, frontal view **C** head and mesosoma, dorsal view **D** metasoma, dorsal view.

#### Pyria stilboides

Taxon classificationAnimaliaHymenopteraChrysididae

Spinola, 1838

Plate 46

Pyria stilboides : Spinola 1838: 446.

##### Type locality.

Egypt.

##### Material.

**Holotype** ♀. *Chrysis nobilis*, Kl. *Pyria producta*, m. ol.[im] D. Waltl, Egyptus.

**Catalogue Casolari & Casolari Moreno.** *Chrysis nobilis*, 132, 23, 0, 2 (box 51).

##### Remarks.

Spinola (1838) described *Pyria stilboides* based on one female. The are two specimens of this species in the Spinola collection; one female, perfectly conserved, which we consider the holotype, and a second specimen, badly damaged, which we exclude from the type series. The names written on the label (and in Casolari & Casolari Moreno) refer to Dahlbom’s monograph (1854: 347), in which *Pyria stilboides* is considered a synonym of *Chrysis nobilis*. The second specimen, according to Spinola’s label and Dahlbom’s work, was named *Chrysis producta*.

##### Current status.

*Chrysis stilboides* (Spinola, 1838) (tranferred by du Buysson (in André) 1896: 649).

**Plate 46.**
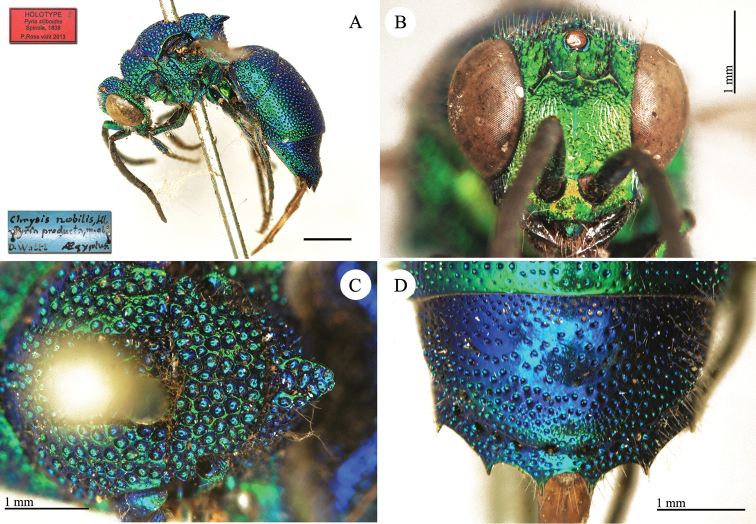
*Pyria stilboides* Spinola, holotype **A** Habitus, lateral view **B** head, frontal view **C** mesosoma, dorsal view **D** third metasomal tergite, dorsal view.

#### Spinolia magnifica

Taxon classificationAnimaliaHymenopteraChrysididae

Dahlbom, 1854

Plate 26D

Spinolia magnifica : Dahlbom 1854: 363 *nec* Brullé, 1846.

##### Type locality.

"Habitat in Turcia, ad Constantinopolum a Dom. Friwaldsky detecta, Mus. Dom. Spinola, qui unicum specimen communicavit".

##### Material.

**Holotype** ♂. *Spinolia magnifica* Dlbm. – *Chrysis varicollis*, m.[ihi] ol.[im] D. Friwaldsky, Constantinopolis.

**Catalogue Casolari & Casolari Moreno.** *Spinolia magnifica*, 27, 69, 30, 1 (box 51).

##### Current status.

*Spinolia lamprosoma* (Förster, 1853) (synonymised by Mocsáry 1887: 16).

### Types not housed in the Spinola collection

The types of the following thirteen species were thought to be part of the Spinola collection (Dahlbom 1854; Abeille 1877; Kimsey and Bohart 1991), but could not be found there. The type of *Pyria canaliculata* is in MNHN; the type of *Euchroeus festivus* Spinola is destroyed by dermestid attack, only the pin without specimen remains.

#### Chrysis aurifascia

Taxon classificationAnimaliaHymenopteraChrysididae

Brullé, 1846

Chrysis aurifascia : Brullé 1846: 40.

##### Remarks.

Holotype unknown. The type locality is South Africa, Cape of Good Hope (Serville coll.). According to Kimsey and Bohart (1991: 387), the holotype should be housed in the Spinola collection, but we could not find any evidence for this assumption. The type should be searched for at MNHN.

##### Current status.

*Chrysis aurifascia* Brullé, 1846.

#### Chrysis aurifrons

Taxon classificationAnimaliaHymenopteraChrysididae

Dahlbom, 1854

Chrysis aurifrons : Dahlbom 1854: 122.

##### Remarks.

Dahlbom (1854: 122) described *Chrysis aurifrons* based on two specimens from the MNHU (found under the name *Chrysis aurifrons* Klug) and the Spinola collection (found under the name Chrysis integra var. chrysocephala). The type locality is "*Italia, Mus. Spinolae*" and "*Asia minori teste D. Loew*". We could not find a syntype neither in the Spinola collection and nor at MNHU. Bohart (in Kimsey and Bohart 1991: 491) designated the lectotype based on one specimen from Italy housed in LZM. In Dahlbom’s collection (LZM), there is one specimen labelled as *Chrysis aurifrons* by Dahlbom, but it is not a syntype and it does not bear any lectotype label by Bohart.

##### Current status.

*Chrysis ignifrons* Brullé, 1833 (synonymized by Mocsáry 1889: 263).

#### Chrysis coronata

Taxon classificationAnimaliaHymenopteraChrysididae

Spinola, 1808

Chrysis coronata : Spinola 1808: 30.

##### Remarks.

*Chrysis coronata* was erroneously placed in synonymy with *Chrysis viridula* Linnaeus, 1761 (Kimsey and Bohart 1991: 477). In fact, the description of *Chrysis coronata* is clear and is incompatible with the coloration and morphology of *Chrysis viridula*: "*Magnitudo praecedentis. Antennae nigrae. Caput viride, maculâ caerulaeâ vericali. Thorax viridis. Abdomen, segmentis duobus anterioribus aureis, 3° toto viridi, margine apostico triemarginato. Corpus subtùs caeruleum. Pedes concolores, tarsis pallidis. Alae fuscae*". Spinola compares *coronata* with *splendidula*: "*Noli confundere cum* Chrys. Splendidula *Rossi, quae differt thorace quadrifasciato, fasciis alternatim viridibus et caeruleis, segmento tertio quadrispinoso, caeruleo, spinulis productis acutis, staturâ tandem in plerisque speciminibus dimidio minore*." Based on this description, *Chrysis coronata* could be *Chrysis rutilans* Olivier or *Chrysis continentalis* Linsenmaier. Since the holotype is lost, we suggest *coronata* should be considered as a **nomen dubium.** According to Mocsáry (1889: 445), *Chrysis coronata* is the male of *Chrysis integra* Fabricius. Till now, *Chrysis integra* has never been found in Italy and the species description of *Chrysis coronata* is incompatible with *Chrysis integra*. *Chrysis coronata* is described as shorter in its dimensions by Spinola (*Long. 3. Lin. Lat. 1. ½ Lin.*), shorter than those of *integra*, which should exceed 5 lin., using the same measurement system used by Spinola.

##### Current status.

*Chrysis coronata* Spinola, 1808, nomen dubium.

#### Chrysis crassimargo

Taxon classificationAnimaliaHymenopteraChrysididae

Spinola, 1843

Chrysis crassimargo : Spinola 1843: 127.

##### Remarks.

Spinola (1843) did not cite the type depository of the specimen collected by Ghiliani. Abeille (1879: 65) wrote that *Chrysis crassimargo* is the female of *Chrysis emarginatula*, but he did not examine the type in the Spinola collection, as he stated in other cases.

##### Current status.

*Chrysis emarginatula* Spinola, 1808 (synonymized by Mocsáry 1889: 231).

#### Chrysis distinguenda

Taxon classificationAnimaliaHymenopteraChrysididae

Spinola, 1838

Chrysis distinguenda : Spinola 1838: 450.

##### Remarks.

The type is not present in the Spinola collection. Dahlbom (1854: 282) examined the type in the Spinola collection, when it was still there, and wrote that it is entirely blue-green: "*quae species est toto corpore cyaneo-viridis*". In Egypt, only few species are known with four anal teeth and blue-green colouration. One of them is *Chrysis blanchardi* Lucas, 1849, described from Algeria, distributed from the Iberian Peninsula via North Africa to Palestine. It could match Spinola’s description. Linsenmaier overlooked this name until his last work (1999: 211), in which he placed *Chrysis distinguenda* in the *Chrysis ignita* group. However, *Chrysis distinguenda* could be a small blue species as found in various species groups (e.g., *Chrysis cerastes*, *Chrysis ignita*, *Chrysis scutellaris*-*viridissima*, *Chrysis pallidicornis*). Therefore, we consider *Chrysis distinguenda* as **nomen dubium.**

##### Current status.

*Chrysis distinguenda* Spinola, 1838, **nomen dubium.**

#### Chrysis fasciata

Taxon classificationAnimaliaHymenopteraChrysididae

Spinola, 1806

Chrysis fasciata : Spinola 1806: 14 *nec* Olivier, 1790.

##### Remarks.

*Chrysis fasciata* Spinola 1806 is a primary homonym of *Chrysis fasciata* Olivier, 1790. The first available name for this species is *Pseudospinolia uniformis* (Dahlbom, 1854). The type depository is unkown.

##### Current status.

*Pseudospinolia uniformis* (Dahlbom, 1854).

#### Chrysis hybrida

Taxon classificationAnimaliaHymenopteraChrysididae

Lepeletier, 1806

Chrysis hybrida : Lepeletier 1806: 127.

##### Remarks.

Abeille (1879: 85) examined one specimen in the Spinola collection that he considered as a type: "*j’ai vu à Turin, dans les cartons de Spinola un type envoyé par Lepelletier luimême et identique avec mes exemplaires*". Unfortunately, we did not find any specimen of *Chrysis hybrida* in the Spinola collection.

##### Current status.

*Chrysura hybrida* (Lepeletier, 1806) (transferred by Kimsey and Bohart 1991: 490).

#### Chrysis simplex

Taxon classificationAnimaliaHymenopteraChrysididae

Dahlbom, 1854

Chrysis simplex : Dahlbom 1854. Hymenoptera Europaea etc., 2: 128.

##### Remarks.

Dahlbom (1854: 128) described *Chrysis simplex* based on two specimens, one coming from MNHU, and still housed there, and a second one from the Spinola collection (Chrysis integra var. maior). This specimen is no longer present in the Spinola collection.

##### Current status.

*Chrysura simplex* (Dahlbom, 1854) (transferred by Kimsey and Bohart 1991: 496).

#### Chrysis splendidula

Taxon classificationAnimaliaHymenopteraChrysididae

Rossi, 1790

Chrysis splendidula : Rossi 1790: 76.

##### Remarks.

Abeille (1877: 6) wrote that the type of *Chrysis splendidula* is housed in the Spinola collection. This was likely erroneous information, since the syntypes are expected to be housed in MNHU, together with other Rossi’s types. And we also found no evidence for the type in the Spinola collection.

##### Current status.

*Chrysis splendidula* Rossi, 1790.

#### Euchroeus quadratus var. festivus

Taxon classificationAnimaliaHymenopteraChrysididae

Dahlbom, 1854

Euchroeus festivus : Dahlbom 1854: 374 (given as *Euchroeus quadratus* var. *b*).

##### Type locality.

"Aegypto, Dom. Waltl, Mus. Dom. Spinola".

##### Type

(destroyed!): *Euchroeus quadratus*, Kl.; D. Waltl, Aegyptus.

**Catalogue Casolari & Casolari Moreno.** *Euchraeus quadratus*, 132, 23, 95, 0 (box 52).

##### Remarks.

The type is destroyed. Only the pin and the label are left. Euchroeus quadratus var. festivus refers to Euchroeus purpuratus var. consularis du Buysson (in André), 1896 (not mentioned in Kimsey and Bohart 1991), which is the only taxon related to *Euchroeus purpuratus* distributed in North Africa (Linsenmaier 1999: 99). *Euchroeus festivus* has priority over *Euchroeus consularis*, but it was not used after 1899, contrary to *consularis*, which is the name currently in use.

##### Current status.

Euchroeus purpuratus ssp. consularis du Buysson (in André), 1896.

#### Hedychrum cyaneum

Taxon classificationAnimaliaHymenopteraChrysididae

Brullé, 1846

Hedychrum cyaneum : Brullé 1846: 52.

##### Remarks.

Holotype ♂.

##### Type locality.

South Africa, Cape of Good Hope (Serville coll.) According to Kimsey and Bohart (1991: 213), the holotype should be housed in the Spinola collection, but it is not there any more. The type should also be searched for at MNHN.

##### Current status.

*Hedychrum cyaneum* Brullé, 1846.

#### Pyria canaliculata

Taxon classificationAnimaliaHymenopteraChrysididae

Brullé, 1846

Pyria canaliculata : Brullé 1846: 20.

##### Remarks.

Holotype ♀.

##### Type locality.

Semegal (Serville coll.). According to Kimsey and Bohart (1991: 393), the holotype should be housed in the Spinola collection, but the type is housed in MNHN, as reported by du Buysson (1897: 570) "Type de l’auteur, ♀" in Brullé’s collection.

##### Current status.

*Chrysis canaliculata* (Brullé 1846) (transferred by Mocsáry 1889: 587).

#### Sphex ignita

Taxon classificationAnimaliaHymenopteraChrysididae

Linnaeus, 1758

Sphex ignita : Linnaeus 1758: 571.

##### Label.

*Chrysis ignita* Lin., et Dab. ♀. ♂.; typus; passim.

**Catalogue Casolari & Casolari Moreno.** *Chrysis ignita*, 157, 0, 0, 4 (box 51).

##### Remarks.

Someone placed a red rounded label, which means "type", near four specimens. None of the four specimens is a type; the true syntypes (lecto- and paralectotype) are housed in LSL. Very likely Spinola wrote "*typus*" on the label to identify the typical form of *ignita* and not a variation.

##### Current status.

*Chrysis ignita* (Linnaeus, 1758) (transferred by Linnaeus 1761: 414).

### Notes on other specimens in the Spinola collection

The following specimens are housed in the Spinola collection and could be types of species described by Dahlbom, Klug, Lepeletier, Spinola, and Wesmael, yet it is difficult or impossible to confirm their type status.

#### Chrysis aurichalca

Taxon classificationAnimaliaHymenopteraChrysididae

Lepeletier, 1806

Chrysis aurichalca : Lepeletier 1806: 127.

##### Label

[♀]: *Chrysis aurichalca* Lep. 127; Europa, Provenza, *Caerulipes* Fab // 6293 (currently *Chrysura cuprea* (Rossi, 1790)).

**Catalogue Casolari & Casolari Moreno.** *Chrysis aurichalca*, 148, 177, 0, 3 (box 50).

##### Remarks.

The specimen with numerical rounded label (6293) could be the holotype of *Chrysis aurichalca* Lepeletier, which arrived in the Spinola collection with the chrysidids of Latreille. The type is not housed in MNHN (du Buysson 1899).

##### Current status.

*Chrysura cuprea* (Rossi, 1790) (transferred by Kimsey and Bohart 1991: 487).

#### Chrysis bicolor

Taxon classificationAnimaliaHymenopteraChrysididae

Lepeletier, 1806

Plate 47

Chrysis bicolor : Lepeletier 1806: 127.

##### Label

[♀]: *Chrysis bicolor*, Lepel. 127; Europa P. / 6288.

##### Material.

**Neotype** (here designated) ♀. France Var St. Laurent d. Verdon [Saint-Laurent-du-Verdon, Alpes-de-Haute-Provence department, France] 23.6.74 Coll. Linsenmaier / [leg.] Perraudin / Chrysis L. bicolor ♀ Lep. det. Linsenmaier 1998 / ex Doubletten LM collection / NML_ENT GBIF_Chr00020564 / **Neotypus** *Chrysis bicolor* Lepeletier, 1806 P. Rosa des. 2013.

##### Remarks.

Morgan (1984: 9) designated the lectotype of *Chrysis bicolor* in MNHN based on a male without a metasoma, writing: "*sufficient characters being present on the thorax and head to fix its identity*". According to du Buysson (1899) there are no types of *Chrysis bicolor* Lepeletier, 1806 in the Lepeletier collection in MNHN. We were able to examine the presumed type series studied by Morgan. The specimen selected as lectotype was previously labelled as "Type" by a former curator, but it must be excluded from the type series, because Lepeletier placed the specimens under the name *Chrysis humeralis*, a species never described. The specimens identified by Lepeletier as *Chrysis humeralis* can be found in the catalogue of du Buysson (1898: 564) in synonymy of Chrysis succincta var. bicolor. This series of three specimens includes two different species. One specimen, bearing the name *humeralis*, is a male of *Chrysis gribodoi* Abeille; a second specimen is a female of *Chrysis illigeri* Wesmael and bears the label "no type status det. Morgan 1981"; the third specimen was selected by Morgan as the male lectotype of *bicolor*. According to a label pinned by Niehuis in 1998, the specimen selected by Morgan is a female and not a male. Either way, this specimen does not match the current interpretation of the species and belongs to *Chrysis illigeri* Wesmael, 1839 (= *Chrysis helleni* Linsenmaier, 1959 in Morgan’s keys). There is no evidence to show that the selected specimen is a syntype. Lepeletier (1806) very likely described *Chrysis bicolor* based on a single specimen ("*Mâle. Je ne sais de quell pays elle est.*"), which must be considered as a holotype by monotypy. Du Buysson (1809, 1899) did not include it in the type series. Since the specimen designated by Morgan as lectotype had been identified by Lepeletier as *Chrysis humeralis*, and not *Chrysis bicolor*, and as this specimens does not correspond to the desciption of *Chrysis bicolor* given by Lepeletier, we do not consider it as syntype and therefore as a lectotype (Art. 74.2 of the Code).

Since many authors do not separate *Chrysis bicolor* from *Chrysis illigeri* (e.g. Kimsey and Bohart 1991: 389; Kunz 1994: 104, etc.), we think that a neotype designation of *Chrysis bicolor* Lepeletier is needed. Moreover, the taxonomic position of the species belonging to the *Chrysis succincta* group is not clear. For example, Kimsey and Bohart (1991) placed *Chrysis illigeri* in synonymy of *Chrysis bicolor* and considered *Chrysis helleni* Linsenmaier as a valid species. But Linsenmaier himself (1997a) placed *Chrysis helleni* in synonymy with *Chrysis illigeri*.

We designate a **neotype** of *Chrysis bicolor* Lepeletier, 1806 using a specimen housed in the Linsenmaier collection at NMLS. It is a female and it bears the following labels: *Chrysis bicolor*: France Var St. Laurent d. Verdon [Saint-Laurent-du-Verdon, Alpes-de-Haute-Provence department, France] 23.6.74 Coll. Linsenmaier / [leg.] Perraudin / Chrysis L. bicolor ♀ Lep. det. Linsenmaier 1998 / ex Doubletten LM collection / NML_ENT GBIF_Chr00020564 / **Neotypus** *Chrysis bicolor* Lepeletier, 1806 P. Rosa des. 2013 (Plate 46).

The neotype matches the modern interpretation of the species according to Linsenmaier (1959: 113, figs. 350, 502, 503; 1997b: 90, fig. 66). *Chrysis bicolor* can be separated from the similar *Chrysis illigeri* by the different shapes of the black spots on the second sternite, the lengths of the malar space, and the shapes of the metanotum in lateral view, as well as various other characters.

##### Current status.

*Chrysis bicolor* Lepeletier, 1806.

**Plate 47.**
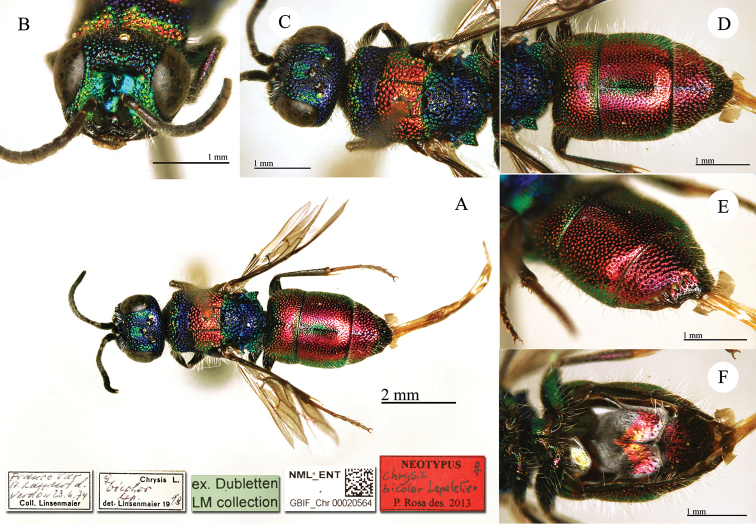
*Chrysis bicolor* Lepeletier, neotype **A** Habitus, dorsal view **B** head, frontal view **C** head and mesosoma, dosal view **D** metasoma, dorsal view **E** metasoma, dorso-lateral view **F** metasoma, ventral view.

#### Chrysis incerta

Taxon classificationAnimaliaHymenopteraChrysididae

Dahlbom, 1854

Chrysis incerta : Dahlbom 1854: 346.

##### Label

[♂]: *Chrysis nobilis*, Kl.; *Pyria producta*, m. ol.; D. Waltl, Egyptus.

**Catalogue Casolari & Casolari Moreno.** *Chrysis nobilis*, 132, 23, 0, 2 (box 51).

##### Remarks.

Along with *Chrysis nobilis* (= *Chrysis stilboides*), there is a second specimen corresponding to the description of *Chrysis incerta* Dahlbom, 1854 in the Spinolia collection. Even though it does not bear labels with locality data (the original description states: "Cajennae, Dom. *Buquet*; Mus. Dom. *Spinola*"), this specimen could be the type of *Chrysis incerta*. Kimsey and Bohart (1991) listed syntype males presumably housed in MRSN and placed *Chrysis incerta* as synonym of *Chrysis stilboides* (Spinola, 1838).

##### Current status.

*Chrysis stilboides* (Spinola, 1838) (synonymized by Kimsey and Bohart (1991: 466).

#### Chrysis semicincta

Taxon classificationAnimaliaHymenopteraChrysididae

Lepeletier, 1806

Chrysis semicincta : Lepeletier 1806: 127.

##### Label

[♀]: "*Chrysis semicincta, Lepell. / Poecilochroa Klug / D. Cantener Provence / D. Ghiliani, Espagne*".

**Catalogue Casolari & Casolari Moreno.** *Chrysis semicincta*, 148, 177, 10, 1 (box 51).

##### Remarks.

The type is not housed in the Lepeletier collection at MNHN (du Buysson 1898) and Kimsey and Bohart (1991: 461) listed it as being part of the Spinola collection. There is only one specimen, without a label, housed in MRSN under the name *Chrysis semicincta*. Unfortunately, there is no evidence that this is the true type of this species. Previously, at least two specimens examined by Dahlbom (1854: 242) were housed in the Spinola collection, as written on the main label: "*Chrysis semicincta, Lepell. / Poecilochroa Klug / D.*[Donavit] *Cantener Provence / D.*[Donavit] *Ghiliani, Espagne*". The type locality of *Chrysis semicincta* is unknown: "*Je ne sais de quel pays elle est*". The locality of the specimen left in the collection is unknown, too, since no locality label is pinned with the specimen. We know that part of the Lepeletier collection, and therefore some types, had been primarily bought by Cantener (Passerin d’Entréves 1980) and later bought by Spinola (letter 00576). It is possible that Spinola bought the specimen from Cantener and wrote "Provence", the locality given by the insect dealer. We observed that the specimen had been prepared with open wings, as the one drawn on the colour plate by Lepeletier (1806). Lepeletier (1806) described *Chrysis semicincta* from a male, but Lepeletier was often unable to identify the gender of cuckoo wasps correctly, so this could be a useless piece of information as in the case of *Hedychrum nitidum*.

##### Current status.

*Chrysis semicincta* Lepeletier, 1806.

#### Chrysis succincta

Taxon classificationAnimaliaHymenopteraChrysididae

Linnaeus, 1767

Plate 48A–G, I

Chrysis succincta : Linnaeus 1767: 947.

##### Material.

**Neotype** (here designated) ♂. Bromberg [currently Bydgoszcz, Kuyavian-Pomeranian Voivodeship, Poland] 24.V.20 leg. dr. Meyer Coll. Linsenmaier / *Chrysis* L. *succincta* L. Linsenmaier det. 59 / ex synoptic collection / NML_ENT GBIF_Chr00021185.

##### Remarks.

The description of *Chrysis succincta* given by Linnaeus (1767) is very short, but concise and precise. Linnaeus described it with "*abdomine aureo subtridentato*". The specimen, or the specimens, examined by Linnaeus were females belonging to the species now identified as *Chrysis illigeri* Wesmael or *Chrysis bicolor* Lepeletier. The females of these species have four teeth on the anal margin, but the two median teeth are very close, at first sight with a low magnifying glass may appear merged into a single tooth, therefore displaying a "subtridentato" appearance.

Today, the name *Chrysis succincta* Linnaeus is erroneously attributed to a species with the anal margin of the third tergite simple, rounded, sub-oval, and toothless. This misinterpretation has already been pointed out by Niehuis (in Mandery and Niehuis 2000: 51). *Chrysis illigeri* and *Chrysis bicolor* are distributed in all Europe and they are quite frequent or common in central and northern Europe, whereas *Chrysis succincta* *sensu* Linsenmaier 1959 is a central European species, whose range appears to be restricted to Germany and Poland, although it is possible that its distribution went further north in Linnaeus’ time. Unfortunately the type of *Chrysis succincta* must be considered lost; it is not housed in LSL, NHRS, or LMU.

Jurine (1807: 295) was the first author to identify a specimen with a complete anal margin bearing the name *Chrysis succincta*: "*Je n’ai pu reconnaitre ni dents, ni échancrures au dernier segment du ventre*". Later, Wesmael (1839: 176, 177) followed Jurine’s interpretation. Wesmael described *Chrysis illigeri* with: "*ano utrinque emarginato, in medio bidentato*" in contrast with his interpretation of *Chrysis succincta*: "*ano utrinque oblique subemarginato, in medio obtuso*". After this paper, all the main authors considered *succincta* as a species with a toothless anal margin.

Linsenmaier (1959) described the *Chrysis succincta* species group based on the misidentified *succincta*, and later Kimsey and Bohart (1991) described the *Chrysis succincta* *sensu* stricto subgroup and the *Chrysis succincta leachii* subgroup. Moreover, most of the species belonging to the *Chrysis succincta* species group have been described as variations, forms, or subspecies of *Chrysis succincta*, or they have been considered, sooner or later, as synonyms of *Chrysis succincta*. For this reason, nowadays, *Chrysis succincta* is erroneously listed as occurring in all the European countries, in northern Africa and eastwards to central Asia. The easiest way to solve the problem would be to suppress the name *Chrysis succincta*. Since it is improper to ask for the suppression of a name given by Linnaeus, we designate a neotype based on one specimen identified as *Chrysis succincta* by Linsenmaier, the only author who gave a detailed description of the species in the modern sense. The selected specimen is a male housed in the Linsenmaier collection (NML), and bearing the following labels: Bromberg [currently Bydgoszcz, Kuyavian-Pomeranian Voivodeship, Poland] 24.V.20 leg. dr. Meyer Coll. Linsenmaier / *Chrysis* L. *succincta* L. Linsenmaier det. 59 / ex synoptic collection / NML_ENT GBIF_Chr00021185. The neotype matches Linsenmaier’s description of this species (1959: 114, Figs 340, 490). The decision to designate this neotype in the Linsenmaier collection was done after consultation with other specialists (Arens, Paukkunen, Pavesi, Soon, Wiśniowski) as recommended by the Code (Recommendation 75B0. Redescription of *Chrysis succincta* Linnaeus, 1767).

##### Male.

Length: 6.8 mm.

*Colour*. Head: face metallic greenish with bronze to reddish reflections on lateral sides of scapal basin, TFC, clypeus, scapus, pedicel and F-I; rest of flagellum blackish without metallic reflections; vertex greenish, area between ocelli darker with bluish to blackish intervals between the punctures; occiput greenish to bluish. Mesosoma: pronotum greenish, anterior margin with golden reflections, posterior margin bluish; mesonotum greenish to golden, not evidently in contrast with the colour of pronotum and scutellum, as in the male of *Chrysis bicolor* Lepeletier; scutellum greenish, metanotum and propodeum greenish to bluish; mesopleuron greenis with golden reflections; femur and tibia greenish with golden reflections, more evident on tibia; tarsi testaceous. Metasoma: anteriorly greenish becoming gradually reddish posteriorly, anal margin with violet reflections; sternites and laterotergite reddish, with two large black spots on S-II.

*Head*. Scapal basin limited on the upper part by a sort of ring; it covers the entire face between the compound eyes, it is densely and finely punctuated except along the transversal median line, where the punctuation is characterized by longitudinal wrinkles. Frons with large and irregular punctures between the limit of the scapal basin and TFC; TFC not well delineated and vaguely M-shaped; punctures between TFC and mid-ocellus aligned with interspaces directed towards mid-ocellus; punctuation on ocellar area denser and with smaller punctures than on the rest of vertex. Genal carina well developed starting from the base of the mandible. Malar space 1 MOD long. Subantennal space 0.7 MOD. Mandible brown without subapical tooth, metallic greenish proximally. Relative lengths of P / F-I / F-II / F-III: 1 / 1.4 / 0.7 / 0.8. Short vestiture, hairs about 1 MOD long, longer under the genae.

*Mesosoma*. Pronotum with deep and large antero-median depression, ending 1 MOD before the posterior margin; punctuation double with irregular deep, dense and large punctures, without intervals, but with few small and superficial dots between the large punctures. Similar punctuation on the rest of the mesosoma, on mesonotum with deeper and larger punctures; on scutellum with large punctures on the anterior half. Propodeal tooth sharp and pointing outward; mesopleuron with scrobal and episternal sulcus evident. Long (about 1 MOD long) and erected hairs on mesosoma and legs.

*Metasoma*. T-II and T-III with double punctuation, on T-II the diameter of the larger punctures is slightly decreasing towards the posterior margin; preapical pits large and deep; apical margin of T-III simple, slightly arched, without visible teeth or concavities. Black spots on S-II large and elongated, almost in touch at their base and exceeding the middle of the sternite along the lateral margin. Hairs short (less than 1 MOD), longer at the base of T-I.

**Plate 48.**
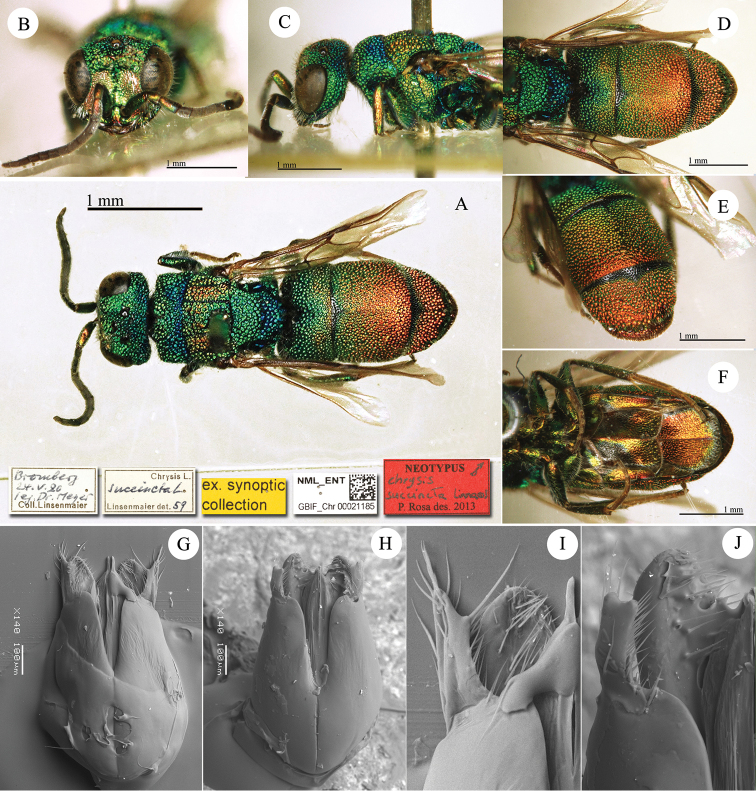
**A–G, I** *Chrysis succincta* Linnaeus, neotype **H, J** *Chrysis tristicula* Linsenmaier **A** Habitus, dorsal view **B** head, frontal view **C** head and mesosoma, lateral view **D** metasoma, dorsal view **E** metasoma, dorso-lateral view **F** metasoma, ventral view **G, H** genitalia **I, J** gonostyle.

*Genital capsula* (Plate 48G, I). In dorsal view, gonocoxa with internal profile gently rounded, with short gonostyle; apex of the gonostyle simple with small subapical lobe bearing a long bristle.

##### Diagnosis.

*Chrysis succincta* mostly resemble *Chrysis frivaldszkyi* Mocsáry and *Chrysis tristicula* Linsenmaier (= *succinctula* *sensu* Linsenmaier) in respect of the shape of the anal margin, the general habitus and colour, especially of the females. It can be easily separated from the male of *Chrysis frivaldszkyi* by the distinctively different shape of its genital capsula (Rosa 2005: figs 19a, b, c) and by the different body colour; the females of *Chrysis frivaldszkyi* and *Chrysis tristicula* are very difficult to separate from the female of *Chrysis succincta* based on morphological characteristics. However, their distribution in Europe is non-overlapping, with *Chrysis succincta* being distributed in Germany, Poland, and the Baltic countries, whereas *Chrysis frivaldskzyi* is distributed in the SE Europe, from Italy to Dalmatia, Austria, Hungary, Czech Republic, Bulgaria, Greece, Ukraine, and eastwards to Middle East. *Chrysis tristicula* is distributed in SW Europe (Italy, Switzerland, France, Iberian Peninsula) and North Africa (Morocco, Tunisia, Egypt?). Males of *Chrysis tristicula* can be separated based on the shape of their genital capsula when seen in dosal view: it has a different stout gonostyle, with two aligned apical lobes (Plate 48H, J). Males of *Chrysis tristicula* also show a different colouration, having head and thorax blue with few light blue to greenish reflections, and red flame anterior drawing on pronotum, mesonotum, anterior angles of metanotum and metasoma, as in *Chrysis illigeri* Wesmael, 1839; the shape of the black spots on the second sternite can gradually vary within the European down to the African specimens, but are always more separated (about 2 MOD) than in *succincta*.

*Chrysis semistriata* Linsenmaier is very similar to *Chrysis tristicula*, but it seems to be restricted to Sardinia and Corsica, and it was considered as an endemic Sardinian species (Rosa 2005). It shows small chromatic and morphological differences to *Chrysis tristicula*. It belongs to the *Chrysis succincta* group.

##### Current status.

*Chrysis succincta* Linnaeus, 1767.

#### Chrysis westermanni

Taxon classificationAnimaliaHymenopteraChrysididae

Spinola, 1838

Chrysis westermanni : Spinola 1838: 454.

##### Type locality.

Guinea.

##### Type

(sex unknown): lost.

##### Label.

*Chrysis amethystra* (sic!) Fab.; *Chrysis Westermanni*, m. olim; Coll. Latr., Ind. [ie] or. [ientali] / Ind. [ie] or. [ientali].

**Catalogue Casolari & Casolari Moreno.** *Chrysis amethystra* (sic), 59, 128, 51, 3 (box 51).

##### Remarks.

Three specimens are found under the main label "*Chrysis amethystra* Fab., *Chrysis Westermanni*" in the Spinola collection. At least two of these specimens were examined by Dahlbom (1854: 229) and placed in synonymy of *Chrysis amethystina* Fabricius. In Kimsey and Bohart (1991: 567), *Chrysis amethystina* is placed in synonymy of *Stilbum cyanurum* (Forster, 1771). The name *Chrysis westermanni* Spinola is not mentioned by Kimsey and Bohart (1991). In fact, the name *Chrysis westermanni* Spinola has been forgotten by all subsequent authors working on cuckoo wasps. Its description is hidden within the description of *Chrysis singularis* Spinola, 1838, but even if short, it is valid: "*J’ai dit aussi que plusieurs espèces du même genre avaient un bourrelet sur le troisième segment; sa présence est assez rare: on en voit des rudiments dans quelques espèces exotiques, telles que les* Chr. sex-dentata, fasciata, *et dans une troidième inédite de la Guinée*, Chrysis Westermanni, *du nom du naturaliste qui l’a recueillie; elles font le passage à la suivante, où le bourrelet est très-apparent*".

Dahlbom (1854: 232) described *Chrysis westermanni* based on one specimen collected by Westermann in Guinea, presumably the same specimen studied some years before by Spinola. In fact, it is possible that Spinola examined Westermann’s specimen and sent it back to the owner; they were in contact and exchanged material. A few years later, Dahlbom received the chrysidid collection of Westermann (Dahlbom 1854: vi) and described *Chrysis westermanni* presumably based on the same specimen studied by Spinola. Now this type is housed in ZMUC.

The name *Chrysis westermanni* Dahlbom, 1854 is therefore a junior homonym of *westermanni* Spinola, 1838. However, the name *Chrysis westermanni* Spinola was never used. Therefore, to ensure the stability of the system, we will ask to the Commission on the ICZN to suppress the name *Chrysis westermanni* Spinola.

#### Elampus spina

Taxon classificationAnimaliaHymenopteraChrysididae

Dahlbom, 1854

Elampus spina : Dahlbom 1854: 41 *nec* Lepeletier, 1806.

##### Label

[♀]: *Elampus spina*, Lep. - *Panzeri* var.; Coll. Latr. France / *Elampus* sp. ♀ det. L.D. French.

**Catalogue Casolari & Casolari Moreno.** *Elampus spina*, 148, 97, 51, 5 (box 50).

##### Remarks.

Dahlbom (1854) described *Elampus spina* based on a specimen collected by Latreille and housed in the Spinola collection. Abeille (1878: 1, 2) examined one specimen which he considered as the type and he replaced the name *spina* Dahlbom with *superbus* Abeille ("*nomen à changer*"). The holotype described by Dahlbom is now housed in his collection in LZM. The female specimen housed in the Spinola collection cannot be considered as a type. *Elampus spina* is a secondary junior homonym of *Elampus spina* (Lepeletier) and a junior synonym of *Elampus bidens* (Förster, 1853). The synonym was already recognized by various authors: Mocsáry (1889: 73), Dalla Torre (1892: 10), etc.

##### Current status.

*Elampus bidens* (Förster, 1853) (synonymised by Mocsáry 1889: 73).

#### Ellampus affinis

Taxon classificationAnimaliaHymenopteraChrysididae

Wesmael, 1839

Ellampus affinis : Wesmael 1839: 172.

##### Label

[Sex unknown]: *Elampus affinis* Wesm. / *Omalus aeneus* Dhlbm. / 3.... Genes / 6. D. [Donavit] Wesmael, Belgique.

**Catalogue Casolari & Casolari Moreno.** *Elampus affinis*, 267, 110/39, 96, 2 (box 50).

##### Remarks.

Based on Spinola’s label, one specimen of this species in the Spinola collection was collected in Belgium and donated by Wesmael to Spinola. It is badly damaged by dermestids and it lacks the metasoma and legs. Howerer, it is possible to identify it as *Omalus aeneus* (Fabricius, 1787). Leclerq (1988: 6) listed the holotype of *Ellampus affinis* at IRSN in Wesmael’s collection. Rosa (2009: 217) found another possible syntype in Gribodo/s collection in MSNG. The second specimen belongs to *Holopyga ignicollis* Dahlbom and comes from Latreille’s or Serville's collection.

##### Current status.

*Omalus aeneus* (Fabricius, 1787) (synonymised by Dahlbom 1854: 36).

#### Hedychrum alterum

Taxon classificationAnimaliaHymenopteraChrysididae

Lepeletier, 1806

Hedychrum alterum : Lepeletier 1806: 122.

##### Label

[♂♀]: *Hedychrum alterum*, Lepel. 122; Europa. P. / 6268 and 6269.

**Catalogue Casolari & Casolari Moreno.** *Hedychrum alterum*, 148, 85, 0, 5 (box 50).

##### Remarks.

*Hedychrum alterum* was traditionally considered as a synonym of *Hedychrum nobile* (Scopoli). No specimen labelled as *Hedychrum alterum* was found in Lepeletier’s collection at MNHN (du Buysson 1898, 1899). Therefore, there is the possibility that the specimens in the Spinola collection are syntypes; the male in this collection belongs to the species *Hedychrum gerstaeckeri* Chevrier, 1869, whereas the female belongs to *Hedychrum rutilans* Dahlbom, 1854. du Buysson (1895: 18) in his "Catalogue méthodique des Chrysidides de France" listed *Hedychrum alterum* as a synonym of *Hedychrum rutilans*, without having considered the priority of the name *Hedychrum alterum* over *Hedychrum rutilans*. The short description of *Hedychrum alterum* does not allow identification of this species and part of the description is doubtful ("*Tête et corcelet verts*") because the colour of head and pronotum and mesonotum in *Hedychrum nobile* has no contrasts. Confidence in that Lepeletier described at least one specimen of *Hedychrum rutilans* comes from the colour drawing (Lepeletier 1806: pl. 6: fig. 8). In this drawing, it is clear that pronotum and mesonotum are green, contrasting with the rest of the mesosoma, which is blue. Therefore, based on the drawing and the diagnosis given by du Buysson (1899), we can assume the synonym *Hedychrum rutilans* Dahlbom, 1854 = *Hedychrum alterum* Lepeletier, 1806.

*Hedychrum alterum* was misinterpreted by Dahlbom (1854: 79), who placed it in synonymy with *Hedychrum lucidulum* (Fabricius, 1775) [currently *Hedychrum nobile* (Scopoli, 1763)] without having examined the type. He simply listed "*Hedychrum alterum* Dufour in litt.". Based on this work, all the other authors, from Dalla Torre (1892: 34) to Kimsey and Bohart (1991: 217) placed *Hedychrum alterum* in synonymy with *Hedychrum nobile*.

Since the name *Hedychrum rutilans* is currently in prevailing usage, we propose the reversal of precedence in accordance with the Art. 23.9 of the Code. In fact, the prevailing usage must be maintained when the two conditions are both met: the senior synonym has not been used as a valid name after 1899 and the junior synonym has been used for this species as a valid name in at least 25 works published by at least 10 authors in the immediately preceding 50 years and encompassing a span of not less than 10 years. The name *Hedychrum alterum* Lepeletier was placed in synonymy with *Hedychrum nobile* by Mocsáry (1889: 172) and has never been used again as a valid species name. In contrast, more than then authors have used the name *Hedychrum rutilans* as a valid species name during the last 50 years in dozens of publications. Here are some of the most important papers from different countries: Morgan (1984: 16); Leclerq (1988: 8); Kimsey and Bohart (1991: 219); Kunz (1994: 93); Mingo (1994: 67); Strumia (1995: 3); Mandery and Niehuis (2000: 55); Niehuis (2001: 121); Rosa (2002: 106; 2005: 23; 2006: 150); Schneider (2002: 178); Burger (2003: 7); Peeters et al. (2004: 193); Drozdovskaya (2006: 101); Schljachtenok (2006: 287); Yildirim and Strumia (2006: 965); Kurzenko and Lelej (2007: 1003); Ljubomirov (2007: 530); Tyrner (2007: 47); Kroiss et al. (2008; 2009); Livory et al. (2008: 28); Szczepko and Wiśniowski (2009: 174); Orlovskyte et al. (2010: 146); Schmid-Egger (2011: 35). Since the name *Hedychrum rutilans* is in prevailing usage and both conditions requested by the code for preserving a prevailing species name are met, we consider the name *Hedychrum rutilans* as **nomen protectum** and the senior synonym *Hedychrum alterum* as **nomen oblitum.**

##### Current status.

*Hedychrum alterum* Lepeletier, 1806, **nome oblitum.**

#### Hedychrum aulicum

Taxon classificationAnimaliaHymenopteraChrysididae

Spinola, 1843

Hedychrum aulicum : Spinola 1843: 129.

##### Label

[♂]: *Hedychrum lucidulum*, v.[ar.] d. ♂. - *Hedychrum aulicum*, D. Crist.; D. de Cristofori. Mediolanum.

**Catalogue Casolari & Casolari Moreno.** *Hedychrum lucidulum*, 0, 160, 19, 3 (box 50).

##### Remarks.

Spinola (1843) described *Hedychrum aulicum* based on some males received by De Cristofori from Dalmatia, Sicily, and Spain. Dahlbom (1854: 79) examined only one male and placed it in synonymy with *Hedychrum lucidulum* (= *Hedychrum nobile*). There are still three specimens of this species in the Spinola collection: one bears a round label and it was part of Latreille’s or Serville’s collection; we exlude it from the type series. The second specimen belongs to *Hedychrum niemelai* Linsenmaier and we exclude it from the type series as it is a female. The third specimen is a male and could be considered as a syntype. We cannot identify the species for sure, because it is badly prepared and a new preparation might damage the specimen. The specimen likely belongs to *Hedychrum niemelai*. We suggest considering the name *Hedychrum aulicum* as **nomen oblitum** and *Hedychrum niemelai* as **nomen protectum**, since it is in prevailing usage in the last fifty years and the name *Hedychrum aulicum* has not been used after 1899.

##### Current status.

*Hedychrum aulicum* Spinola, 1843, **nomen oblitum.**

#### Hedychrum bidentulum

Taxon classificationAnimaliaHymenopteraChrysididae

Lepeletier, 1806

Plate 49

Hedychrum bidentulum : Lepeletier 1806: 121.

[♂]: *Hedychrum bidentulum*, Lepel. 121; Europa / *nitidus* Panz. 97.17. (currently *Philoctetes bidentulus*).

##### Material.

**Neotype** (here designated) ♂. *Hedychrum bidentulum*: Machecoul [Loire-Atlantique department, France] (Loire-Inférieur) Dr. Marmottan 7.1901 / *Omalus* Pz. *biaccinctus* Lep. Linsenmaier det. / NML_ENT GBIF_Chr00001055 / **Neotypus** *Hedychrum bidentulum* Lepeletier, 1806 P. Rosa des. 2013.

**Casolari & Casolari Moreno:** Cleptes (sic!) bidentulum, 148, 85, 0, 5 (box 50).

##### Remarks.

Lepeletier (1806) described *Hedychrum bidentulum* based on a male collected in the environs of Paris. There are five specimens under the name *Hedychrum bidentulum* at the MNHN: two of them belong to the species *Pseudomalus auratus* (Linnaeus, 1758) and three specimens are *Pseudomalus pusillus* (Fabricius, 1804) (Buysson 1897: 563). No specimen is labelled as "type"; other specimens found in the historical collections of Bosc and Lucas and identified as *bidentulus* or *bidentatus* belong to the species *Pseudomalus auratus* (Linnaeus); in the general collection (including du Buysson’s collection) and Sichel’s collection there are some specimens of *Hedychrum bidentulum*, collected in different localities. Casolari and Casolari Moreno (1980: 77) placed erroneously *bidentulum* in the genus *Cleptes*. The original label is: "*Hedychrum* [*Hedychrum*] *bidentulum* Lepel. 121". Two of the five specimens identified as *bidentulum* were received by Latreille or Serville and bear a round and numbered label. The one bearing the number 1651 is truly a *Hedychrum bidentulum* and could be the type of *Hedychrum bidentulum*.

Kimsey and Bohart (1991: 245) placed *Hedychrum bidentulum* in synonymy with *Omalus aeneus* (Fabricius, 1787). No European author ever accepted this synonym. Strumia (1995: 4) included *Hedychrum bidentulum* in the genus *Pseudomalus*, but the correct placement is in the genus *Philoctetes* (Niehuis 2001: 121; Rosa 2003: 305; 2005: 126).

Since the holotype of *Hedychrum bidentulum* is currently lost, since this species is widely distributed in Europe and common at some localities, we designate a neotype to fix the interpretation of this species *sensu* Linsenmaier (1959). The specimen is a male housed in the Linsenmaier collection at NML and bears the following labels: *Hedychrum bidentulum*: Machecoul [Loire-Atlantique department, France] (Loire-Inférieur) Dr. Marmottan 7.1901 / *Omalus* Pz. *biaccinctus* Lep. Linsenmaier det. / NML_ENT GBIF_Chr00001055 / **Neotypus** *Hedychrum bidentulum* Lepeletier, 1806 P. Rosa des. 2013.

The neotype matches the interpretation of the species given by European authors and some drawings and photographs can be found, for example, in Kunz (1994: 49, figs 58, 62), Linsenmaier (1997b: 49, fig. 18), and Rosa (2006: tav III: figs 19, 21).

##### Current status.

*Philoctetes bidentulus* (Lepeletier, 1806) (tranferred by Niehuis 2001: 121).

**Plate 49.**
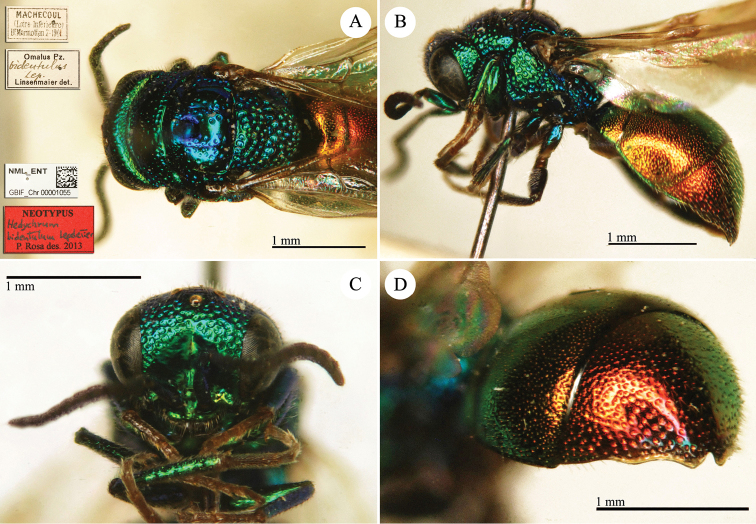
*Hedychrum bidentulum* Lepeletier, neotype. **A** mesosoma, dorsal view **B** habitus, lateral view **C** head, frontal view **D** second and third metasomal tergites, dorso-lateral view.

#### Hedychrum cupreum

Taxon classificationAnimaliaHymenopteraChrysididae

Dahlbom, 1845

Hedychrum cupreum : Dahlbom 1845: 3.

##### Label

[♀]: *Hedychrum cupreum*; Dhlbm. ♀; D.[Donavit] Dahlbom, Scania.

**Catalogue Casolari & Casolari Moreno.** *Hedychrum cupreum*, 27, 187, 20, 2 (box 50).

##### Remarks.

In the Spinola collection, there are two specimens of *Hedychridium cupreum* (Dahlbom, 1845) given by Dahlbom. Dahlbom described this species based on female specimens collected in Sweden. It is possible that the two specimens in the Spinola collection are syntypes sent to Spinola by Dahlbom. Abeille (1879: 39) wrote "*J’ai vu à Turin* [coll. Spinola], *sous le nom primitif de Cupreum, deux types de cette charmante espèce venant de Danemark et envoyés par Dahlbom*". The locality of Denmark given by Abeille represents likely an error since Scania is a Swedish region. These two specimen have been already considered as possible paralectotypes in Paukkunen et al. (2014).

##### Current status.

*Hedychridium cupreum* (Dahlbom, 1845) (transferred by du Buysson (in André) 1896: 746).

#### 1 Hedychrum lucidum

Taxon classificationAnimaliaHymenopteraChrysididae

Lepeletier, 1806

Hedychrum lucidum : Lepeletier 1896: 122.

##### Label

[♀]: *Hedychrum lucidum*, Lepel; 6266.

**Catalogue Casolari & Casolari Moreno.** *Hedychrum ovatum*, 27, 9, 68, 4 (box 50).

##### Remarks.

Lepeletier (1806) described *Hedychrum lucidum* based on one male and one female collected at Meudon and Soissons. There are no syntype specimens in the Lepeletier collection at MNHN (du Buysson 1899). The specimen labelled as "*Hedychrum lucidum* Lepel." and "6266" in the Spinola collection could be a syntype. It is a female specimen belonging to *Holopyga jurinei* Chevrier, 1862, according to the current interpretation of the species given by Linsenmaier (1959).

##### Current status.

*Holopyga lucidum* (Lepeletier, 1896) (transferred by Gogorza 1887: 38).

#### Hedychrum nitidum

Taxon classificationAnimaliaHymenopteraChrysididae

Lepeletier, 1806

Hedychrum nitidum : Lepeletier 1806: 121.

##### Label

[2 ♀♀]: *Hedychrum nitidum*, Lepel. 121; Europa P. / 6265.

**Catalogue Casolari & Casolari Moreno.** *Hedychrum nitidum*, 148, 85, 0, 2 (box 50).

##### Remarks.

Lepeletier (1806) described *Hedychrum nitidum* based on a male, but the description is obviously based on a female. The original colour drawing (pl. 6: 6) clearly shows a specimen with ovipositor tube. Moreover, the colour given in the description likely referres to a female. In the Spinola collection there are two females, possibly syntypes. At MNHN, there are other specimens of this species coming from Lepeletier’s collection, included some syntypes (du Buysson 1898: 563, as a synonym of *Holopyga fervida*, currently in box 11 of the general collection).

##### Current status.

*Holopyga fervida* (Fabricius, 1781) (synonymized by Mocsáry 1889: 131).

#### Hedychrum spina

Taxon classificationAnimaliaHymenopteraChrysididae

Lepeletier, 1806

Hedychrum spina : Lepeletier 1806: 121.

##### Label

[♀]: *Elampus spina* Lep. - *Panzeri* var.; Coll. Latr. France / 6262.

**Catalogue Casolari & Casolari Moreno.** *Elampus panzeri*, 148, 97, 51, 5 (box 50).

##### Remarks.

The type of *Elampus spina* (Lepeletier) could be housed at MNHN or in the Spinola collection. A specimen of this species at MNHN is labelled: *E. Panzeri* F. <handwritten by Lepeletier> / *Hedychrum spina* Lep. type? <handwritten by du Buysson> / Museum Paris Meudon (S. et. O.) Coll. Le Peletier 160-45 <partially printed, locality handwritten by du Buysson>. This specimen was not considered as a type by du Buysson (1898: 563; 1899: 160), because when arrived at MNHN it was labelled only as *Elampus panzeri* by Lepeletier, without locality or identification label. It perfectly matches the original description by Lepeletier and the colour drawing furnished by the author. We would nowadays identify this specimen as *Elampus constrictus* (Förster, 1853) (*sensu* Móczár 1964a). The specimen in the Spinola collection could alternatively represent the type, but even in this case, there is no certainty. It perfectly matches Lepeletier’s description and it belongs to the species *Elampus constrictus* (Förster, 1853) also.

Lepeletier’s description is clear: "*Tête et corcelet d’un vert bleuâtre; yeux et antennes noirs. Corcelet très-prolongé sous l’écusson. Abdomen doré, troisième segment échancré. Pieds d’un vert bleuâtre; tarses pâle. Ailes à peine enfumée. Mâle. De Meudon*". The description is completed by a colour drawing (pl. 6: 2) which shows a specimen with light green head and mesosoma, golden metasoma and small dimensions, being smaller than *Hedychrum nitidum* [= *Holopyga fervida*] and *Hedychrum lucidum* [= *Holopyga lucida*], and approximately as long as *Hedychridium roseum* [= *Hedychridium roseum*]. Description, colouration, and dimensions are different from the species considered *Elampus spina* *sensu* Linsenmaier (1959) and Móczár (1964b). This species is one of the largest *Elampus* species (up to 9 mm) in Europe, with head and mesosoma dark blue to violet and with a flame red metasoma, sometimens dark red to red-violet on the lateral sides. Even the type locality of *Elampus spina* seems to be out from the current distributional limits of *Elampus spina* *sensu* auctorum.

The misinterpretation of the species that the name *Elampus spina* refers to started with Dahlbom (1845: 41): Spinola sent a specimen to Dahlbom identified as *Elampus spina* (or Elampus panzeri var. spina), which he had received from Latreille, and that clearly differs from the specimen now placed in the Spinola collection under the name *spina* Lep. coll. Latreille.

Dahlbom (1854: 42) did not describe *Elampus spina* Lepeletier, but instead he described a new species received by Spinola and named *Elampus spina* Spinola: "*Habitat in Gallia, a D. Latreille detectus, e cujus Collectione Illustr. Spinola accepit individuum quod describendi caussa mihi benevole misit*". Dahlbom's description matches the description of *Elampus bidens* (Förster, 1853): "*Submagnus 2 1/4 lin. decimal. long. violascenti et purpurascenti-aeneus abdomine cyaneo-viridi, segmento 3:tio ante emarginatnram utrinque bisinuato postscutello mucronato*".

Abeille (1878: 1, 2) replaced the name *Elampus spina* Dahlbom with *superbus* ("*nomen à changer*"). Both *Elampus spina* Dahlbom and *Elampus superbus* Abeille are synonyms of *Elampus bidens*.

Based on the type material examined in MNHN, HNHM, and LZM, the original description and drawing, *Elampus spina* (Lepeletier) (*sensu* Linsenmaier 1959 and Móczár 1964a) is *Elampus frivaldszkyi* (Förster, 1853) (= *Elampus productus* Dahlbom, 1854); *Elampus constrictus* (Förster, 1853) is the junior synonym of *Elampus spina* (Lepeletier, 1806). We suggest the neotype designation of *Elampus spina* and a revision of the European species, based on recent findings.

Moreover, we note that *Elampus panzeri* (Fabricius, 1804) is the valid name for *Elampus scutellaris* (Panzer, 1798). A future revision of the European species belonging to the genus *Elampus* is already planned to put in order the complex taxonomical problems of this genus.

Since 1845 the name *spina* has been considered as a noun and not as an adjective; in fact it was used as an invariable name in the genus *Elampus*, *Ellampus*, *Omalus*, and *Notozus* in more than fifty publications. Kimsey and Bohart (1991) introduced the name *Elampus spinus* in accordance with the genus gender, but in conflict with the historical interpretation of the name and the articles 23.5 (applications to spellings) and 33.3.1 of the ICZN (when an incorrect subsequent spelling is in prevailing usage and is attributed to the publication of the original spelling, the subsequent spelling and attribution are to be preserved and the spelling is deemed to be the correct original spelling).

Only three authors followed the interpretation given to the name by Kimsey and Bohart (*Elampus spinus* (Tyrner 2007), Omalus (Elampus) spinus (Arens 2014, Linsenmaier 1999)) while in other publications the name *Elampus spina* remained in use (Liubomirov 2007, Mingo 1994, Rosa 2005a, 2005b, 2006, 2009, Strumia 1995, 2005, Strumia and Yildirim 2007, 2012, Strumia, Gayubo, Gonzalez 2010, Tussac and Zumeta 2004, Vinokurov 2008).

##### Current status.

*Elampus spina* (Lepeletier, 1806) (transferred by Kimsey and Bohart 1991: 171).

#### Holopyga micans

Taxon classificationAnimaliaHymenopteraChrysididae

Klug, 1835

Holopyga micans : Klug 1835: 90.

##### Label

[sex unknown]: *Elampus fulgidicollis*, Klug.

**Catalogue Casolari & Casolari Moreno.** *Elampus fulgidicollis*, 1, 14, 33, 3 (box 50).

##### Remarks.

The specimens housed in the collection and cited by Dahlbom (1854: 55) and Abeille (1879) as types are not syntypes. Dahlbom (1854) received these specimens from Spinola under the name *fulgidicollis*, but the species was already described by Klug under the name *Holopyga micans*. Abeille (1878: 2) erroneusly replaced the name *Holopyga micans* with *cicatrix* "*Nom à changer, M. Lucas ayant dècrit auparavant sous le nom de* Micans *l*’ H. Ovata". Later, Abeille (1879: 28) "*Jai dù changer le nome de cette espèce, M. Lucas ayant décrit, avant Dahlbom, sous le nom d*’Hedychrum micans, *l*’Holopyga gloriosa (ovata), *confondue avec l*’Hedychrum lucidulum. "*J’ai vu, dans le cartons du Museée de Turin, les types de cette charmante espèce que j’ai reçue d’Espagne (Gogorza)*". We did not find the types of *micans* Klug at MNHU, where we found only the types of micans var. viridis Trautmann, 1926 and micans var. aeneus Trautmann, 1926. The case reported by Abeille (1879) is not a case of secondary homonym, since *Hedychrum micans* belongs to the genus *Hedychrum* and not *Holopyga*. Anyway, no author considered the two species congeneric after 1879. Kimsey and Bohart (1991: 254) used *cicatrix* as the first available name *Holopyga micans* (*nec* *Chrysis micans* Olivier, 1790). However, Olivier did not describe any *Elampus micans*, and the two names were never considered conspecific, therefore *micans* Klug is the valid name, as already written by Mingo (1994: 208).

##### Current status.

*Philoctetes micans* (Klug, 1835) (transferred by Kimsey and Bohart 1991: 254).

#### Spintharis chrysonota

Taxon classificationAnimaliaHymenopteraChrysididae

Dahlbom, 1854

Spintharis chrysonota : Dahlbom 1854: 351.

##### Label

[♀]: *Spintharis chrysonota*, Kl.; D. Draege, Cap. B. Esp.

**Catalogue Casolari & Casolari Moreno.** *Spintharis chrysonota*, 132, 53, 21, 3 (box 52).

##### Remarks.

Kimsey and Bohart (1991: 561) examined the specimen in the Spinola collection and considered it as the holotype. But the true holotype (by monotypy) is housed in the Dahlbom collection in LZM. In the Spinola collection, under the name *chrysonota*, there are three specimens: the male of *Spintharis destituta* Dahlbom, 1854, an undescribed species of the same genus, and the female of *Spintharis chrysonota*, with the tip of the metasoma outside the anal margin. The differences listed by Dahlbom describing *chrysonota* and *destituta* are sexual dimorphic characteristics and the two species should be considered as male and female of the same species (Madl and Rosa 2012).

##### Current status.

*Spintharosoma chrysonota* (Dahlbom, 1854) (transferred by Zimmermann 1959: 32).

#### Spintharis destituta

Taxon classificationAnimaliaHymenopteraChrysididae

Dahlbom, 1854

Spintharis destituta : Dahlbom 1854: 352.

##### Label

[♂]: *Spintharis chrysonota*, Kl.; D. Draege, Cap. B. Esp.

**Catalogue Casolari & Casolari Moreno.** *Spintharis chrysonota*, 132, 53, 21, 3 (box 52).

##### Remarks.

As in the previous case, the specimen considered as type by Kimsey and Bohart (1991: 561) cannot be considered as a type, and the true holotype (monotypy) is housed in the Dahlbom collection in LZM. Dahlbom (1854) cited only one specimen in the description of *chrysonota* ("*qui unicum specimen communicavit*") and *destituta* ("*qui specimen communicavit*"); the specimens left in the Spinola collection cannot be considered as syntypes, even though they definitely belong to the same series of specimens received by Spinola from Draege.

##### Current status.

*Spintharosoma chrysonota* (Dahlbom, 1854) (synonymized by Madl and Rosa 2012: 120).

#### Stilbum connectens

Taxon classificationAnimaliaHymenopteraChrysididae

Dahlbom, 1854

Stilbum splendidum var. connectens : Dahlbom 1854: 358 (given as var. *b*).

##### Label

[♀]: *Stilbum connectens*, m. / *calens* vel *splendidum* var. / Coll. Latr., Bengala / *splendidum* var. b. Dlbm.

**Catalogue Casolari & Casolari Moreno.** *Stilbum connectens*, 1, 42, 51, 1 (box 51).

##### Remarks.

*Stilbum connectens* was listed by Dahlbom as a variation, *Stilbum splendidum* var. *b*. It is not an available name and it is only a variation of *Stilbum cyanurum* (Forster).

##### Current status.

*Stilbum cyanurum* (Forster, 1771).

## Conclusions

The study of the type material is fundamental in systematic entomology. For different reasons, the chrysidid types housed in some imporant collections (e.g. Spinola (MRSN), Radoszkowki (ISEA-PAS), Linsenmaier (NMLS)) were not available in "The Chrysidid Wasps of the World" (Kimsey and Bohart 1991). Moreover, many important European revisional and monographic works published in the 20^th^ Century (Trautmann 1927; Berland and Bernard 1938; Balthasar 1953, 1954; Semenov-Tian-Shanskij and Nikol’skaya 1954; Linsenmaier 1959, 1968, 1997b; Moczar 1967; Semenov-Tian-Shanskij 1967; Kunz 1994; Mingo 1994) as well as few more recent ones (e.g. Tyrner 2007; van der Smissen 2010) have been published without comparative studies of the type material. Exceptions were some publications on the British fauna (Morgan 1984), on the Cleptinae (Móczár 1996, 1997a, 1997b, 1998a, 1998b, 1998c, 2000a, 2000b, 2001), a few small revisional articles (e.g. Niehuis 2000), and a regional checklist (Paukkunen et al. 2014). Most of the authors could study only the types found in their museums (e.g. Trautmann 1927 – MNHU; Berland and Bernard 1938 – MNHM; Moczar 1967 – HNHM), others (e.g. Linsenmaier) the types housed in few museums (HNHM, MNHM, NHRS, NHML). Only some authors (e.g. Móczár 1964b, 1965; Day and Fitton 1977; Day 1979; Kimsey 1988; Niehuis 2000; Rosa 2009) gave detailed diagnoses and descriptions of some chrysidid types described in the 18^th^ and 19^th^ centuries.

After receiving the invitation to write the volume on the Chrysididae for the series Fauna d’Italia (Baccetti and Cravedi 2009), we started a project of reviewing the types related to the European fauna. In fact, we found that many original descriptions, mostly given in Latin or French, did not match the current interpretation of the species. After a first analysis carried out on the type material housed in the most important collections (HNHM, ISEA-PAS, LZM, MHNG, MNHN, MNHU, NHMW, NHRS, NMLS, ZIN, ZMUC), we discovered that more than the 15 % of types really do not match the current interpretation of the species for the Italian fauna, and a more extensive research on the Palaearctic Chrysididae was started.

In this paper, ninety-six types belonging to sixty-seven species were found in the Spinola collection, including the types of three species (*Chrysis carinata* Dahlbom, 1854; *Chrysis exsulans* Dahlbom, 1854 and *Chrysis succinctula* Dahlbom, 1845), whose depositories were previously unknown; the types of eight species (*Chrysis analis* Spinola, 1808; *Chrysis bihamata* Spinola, 1838; *Chrysis elegantula* Spinola, 1838; *Chrysis fasciata* Spinola, 1840; *Chrysis magnifica* Dahlbom, 1854; *Chrysis varicornis* Spinola, 1838; *Chrysis versicolor* Spinola, 1808; *Parnopes fischeri* Spinola, 1838) previously placed with doubt in Spinola collection; and the syntypes of eight species (*Chrysis comparata* Lepeletier, 1806; *Chrysis dichroa* Dahlbom, 1854; *Chrysis rutilans* Dahlbom, 1854; *Hedychrum caerulescens* Lepeletier, 1806; *Hedychrum rutilans* Dahlbom, 1854; *Hedychrum virens* Dahlbom, 1854; *Holopyga ovata* Dahlbom, 1854; *Pyria stilboides* Spinola, 1838) previously recorded only in MNHU, MNHN, or LZM. Some nomenclatural and taxonomic changes of these types are as follows: six neotypes and twenty four lectotypes are designated; five previously designated lectotypes are set aside; two species are considered as nomina dubia, two species as nomina oblita, and another two as nomina protecta; three new synonymies are proposed. The photographs of fifty-three types are given for the first time.

This article is the first of a series concerning the study of the Chrysididae types, mainly focused on the Palaearctic fauna. Reviewing the type material is not only essential to find out what is the correct name to list in a catalogue, but also to ensure long-term stability in nomenclature that helps to shift research from boring taxonomic treatments to research on the distribution, biology, and evolution of these fascinating wasps. A major revision of the European Chrysididae is already planned and has begun with the database project of Fauna Europaea (Rosa and Soon 2012).

## Supplementary Material

XML Treatment for Chrysis aequinoctialis

XML Treatment for Chrysis alternans

XML Treatment for Chrysis analis

XML Treatment for Chrysis armena

XML Treatment for Chrysis assimilis

XML Treatment for Chrysis basalis

XML Treatment for Chrysis bihamata

XML Treatment for Chrysis carinata

XML Treatment for Chrysis chilensis

XML Treatment for Chrysis comparata

XML Treatment for Chrysis cuprea

XML Treatment for Chrysis dichroa

XML Treatment for Chrysis distinctissima

XML Treatment for Chrysis distinguenda

XML Treatment for Chrysis dives

XML Treatment for Chrysis elegans

XML Treatment for Chrysis elegantula

XML Treatment for Chrysis emarginatula

XML Treatment for Chrysis episcopalis

XML Treatment for Chrysis exsulans

XML Treatment for Chrysis fasciata

XML Treatment for Chrysis gayi

XML Treatment for Chrysis grohmanni

XML Treatment for Chrysis incrassata

XML Treatment for Chrysis intricans

XML Treatment for Chrysis laeta

XML Treatment for Chrysis malachitica

XML Treatment for Chrysis megerlei

XML Treatment for Chrysis modica

XML Treatment for Chrysis mucronata

XML Treatment for Chrysis pallidicornis

XML Treatment for Chrysis palliditarsis

XML Treatment for Chrysis pulchella

XML Treatment for Chrysis punctatissima

XML Treatment for Chrysis purpurata

XML Treatment for Chrysis ramburi

XML Treatment for Chrysis refulgens

XML Treatment for Chrysis reichei

XML Treatment for Chrysis rutilans

XML Treatment for Chrysis sicula

XML Treatment for Chrysis singularis

XML Treatment for Chrysis smaragdula

XML Treatment for Chrysis spinigera

XML Treatment for Chrysis splendens

XML Treatment for Chrysis succinctula

XML Treatment for Chrysis truncatella

XML Treatment for Chrysis varicornis

XML Treatment for Chrysis versicolor

XML Treatment for Elampus gayi

XML Treatment for Euchroeus candens

XML Treatment for Euchroeus coerulans

XML Treatment for Hedychrum brasilianum

XML Treatment for Hedychrum caerulescens

XML Treatment for Hedychrum chloroideum

XML Treatment for Hedychrum coelestinum

XML Treatment for Hedychrum difficile

XML Treatment for Hedychrum minutum var. duponti

XML Treatment for Hedychrum incrassatum

XML Treatment for Hedychrum rutilans

XML Treatment for Hedychrum virens

XML Treatment for Holopyga janthina

XML Treatment for Holopyga luzulina

XML Treatment for Holopyga ovata

XML Treatment for Parnopes denticulatus

XML Treatment for Parnopes fischeri

XML Treatment for Pyria stilboides

XML Treatment for Spinolia magnifica

XML Treatment for Chrysis aurifascia

XML Treatment for Chrysis aurifrons

XML Treatment for Chrysis coronata

XML Treatment for Chrysis crassimargo

XML Treatment for Chrysis distinguenda

XML Treatment for Chrysis fasciata

XML Treatment for Chrysis hybrida

XML Treatment for Chrysis simplex

XML Treatment for Chrysis splendidula

XML Treatment for Euchroeus quadratus var. festivus

XML Treatment for Hedychrum cyaneum

XML Treatment for Pyria canaliculata

XML Treatment for Sphex ignita

XML Treatment for Chrysis aurichalca

XML Treatment for Chrysis bicolor

XML Treatment for Chrysis incerta

XML Treatment for Chrysis semicincta

XML Treatment for Chrysis succincta

XML Treatment for Chrysis westermanni

XML Treatment for Elampus spina

XML Treatment for Ellampus affinis

XML Treatment for Hedychrum alterum

XML Treatment for Hedychrum aulicum

XML Treatment for Hedychrum bidentulum

XML Treatment for Hedychrum cupreum

XML Treatment for Hedychrum lucidum

XML Treatment for Hedychrum nitidum

XML Treatment for Hedychrum spina

XML Treatment for Holopyga micans

XML Treatment for Spintharis chrysonota

XML Treatment for Spintharis destituta

XML Treatment for Stilbum connectens
